# Supplementary material for: Functionalized 10-Membered Aza- and Oxaenediynes through the Nicholas Reaction
Source: Molecules. 2022 Sep 17;27(18):6071. doi: 10.3390/molecules27186071 (PMC9502870; doi:10.3390/molecules27186071)

Supporting Information

Functionalized 10-Membered Aza- and Oxa-Enediynes through the Nicholas Reaction

Natalia A. Danilkina[1], Ekaterina A. Khmelevskaya[1], Anna G. Lyapunova[1], Alexander S. D’yachenko[1], Alexander S. Bunev[2], Rovshan E. Gasanov[2], Maxim A. Gureev[3,4], and Irina A. Balova[1,*]

[1] Institute of Chemistry, Saint Petersburg State University (SPbU), Universitetskaya nab. 7/9, 199034 Saint Petersburg, Russian Federation

[2] Medicinal Chemistry Center, Tolyatti State University, 445020 Tolyatti, Russian Federation

[3] Laboratory of chemo- and bioinformatics, I. M. Sechenov First Moscow State Medical University, 119991 Moscow, Russian Federation

[4] Department of Computational Biology, Sirius University of Science and Technology, 354349 Sochi, Russian Federation

* Correspondence: i.balova@spbu.ru

Table of Contents

Copies of NMR ^{1}H, ^{13}C, DEPT, 2D NMR S2

Copies of ESI HRMS spectra for key compounds..S69

^{1}H NMR, $CDCl_3$, 400 MHz

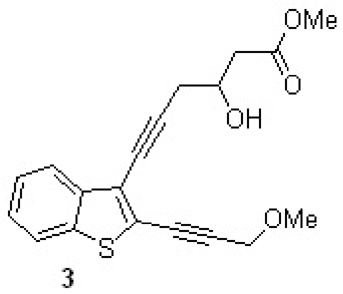

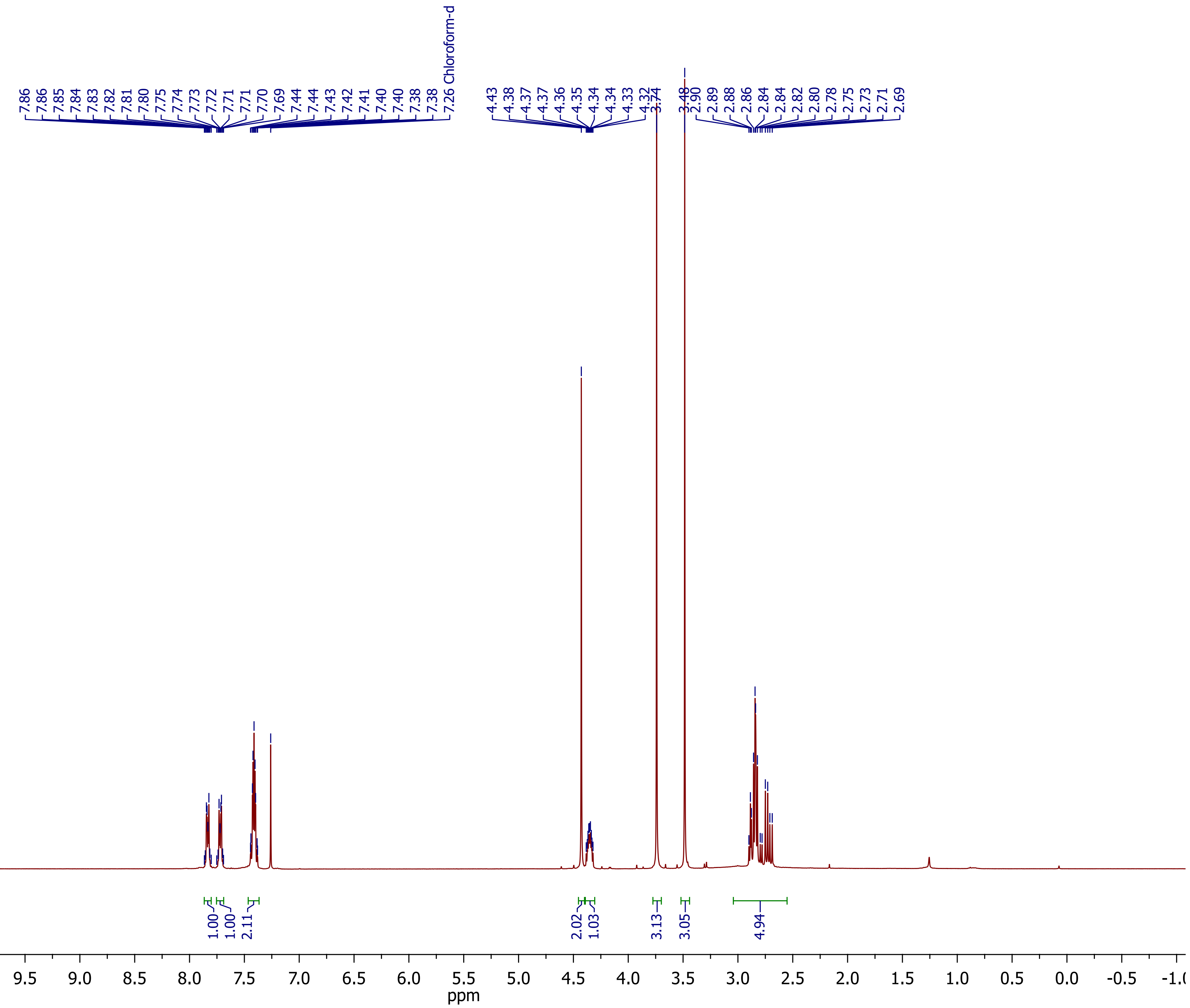

$^{13}C\{^{1}H\}$ NMR, $CDCl_3$, 101 MHz

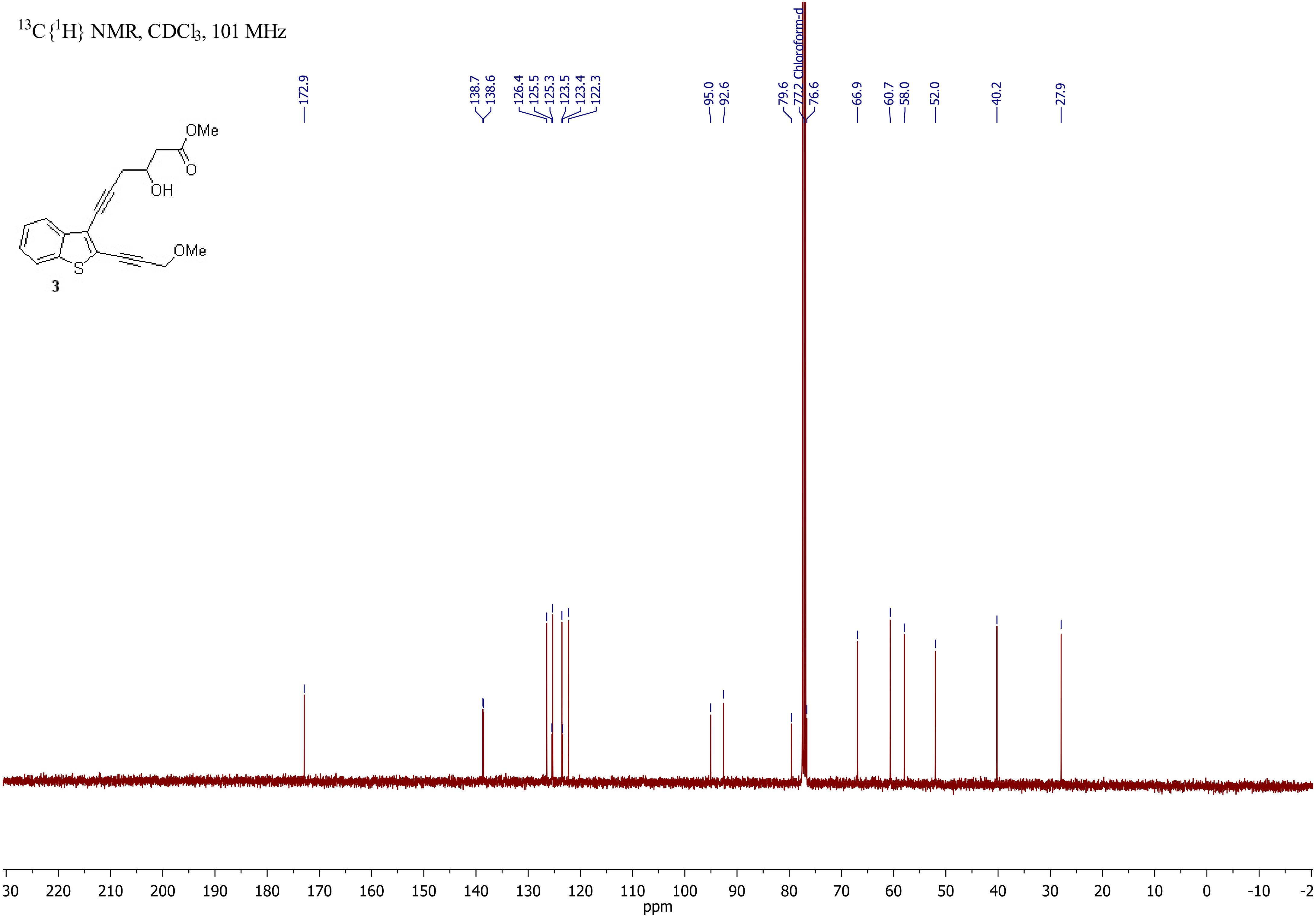

3

DEPT NMR, $CDCl_3$, 101 MHz

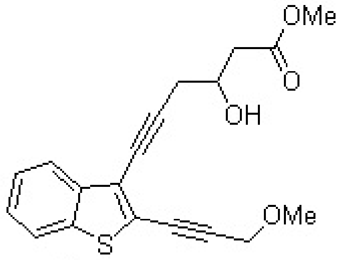

3

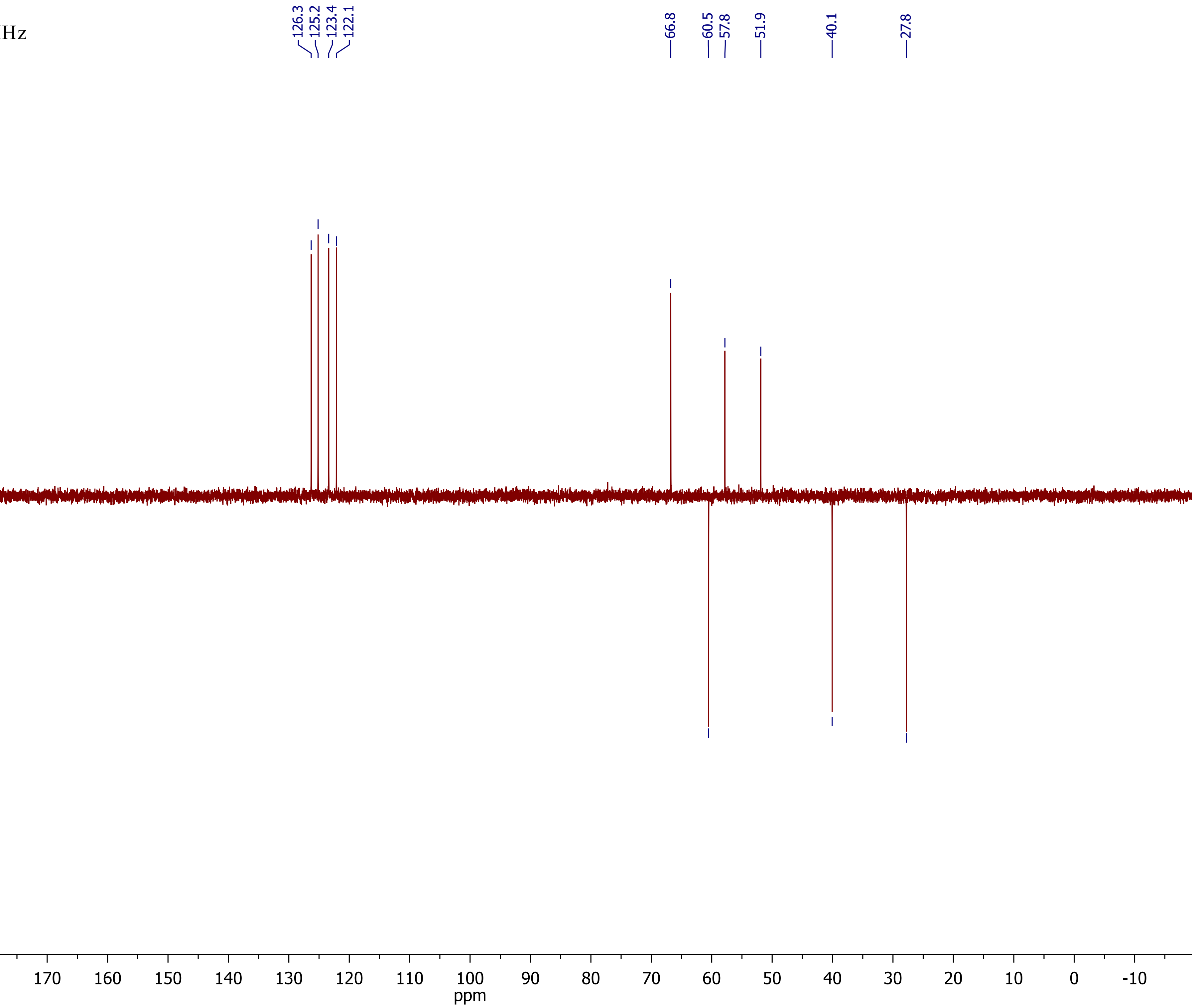

^{1}H NMR, acetone-d_6, 400 MHz

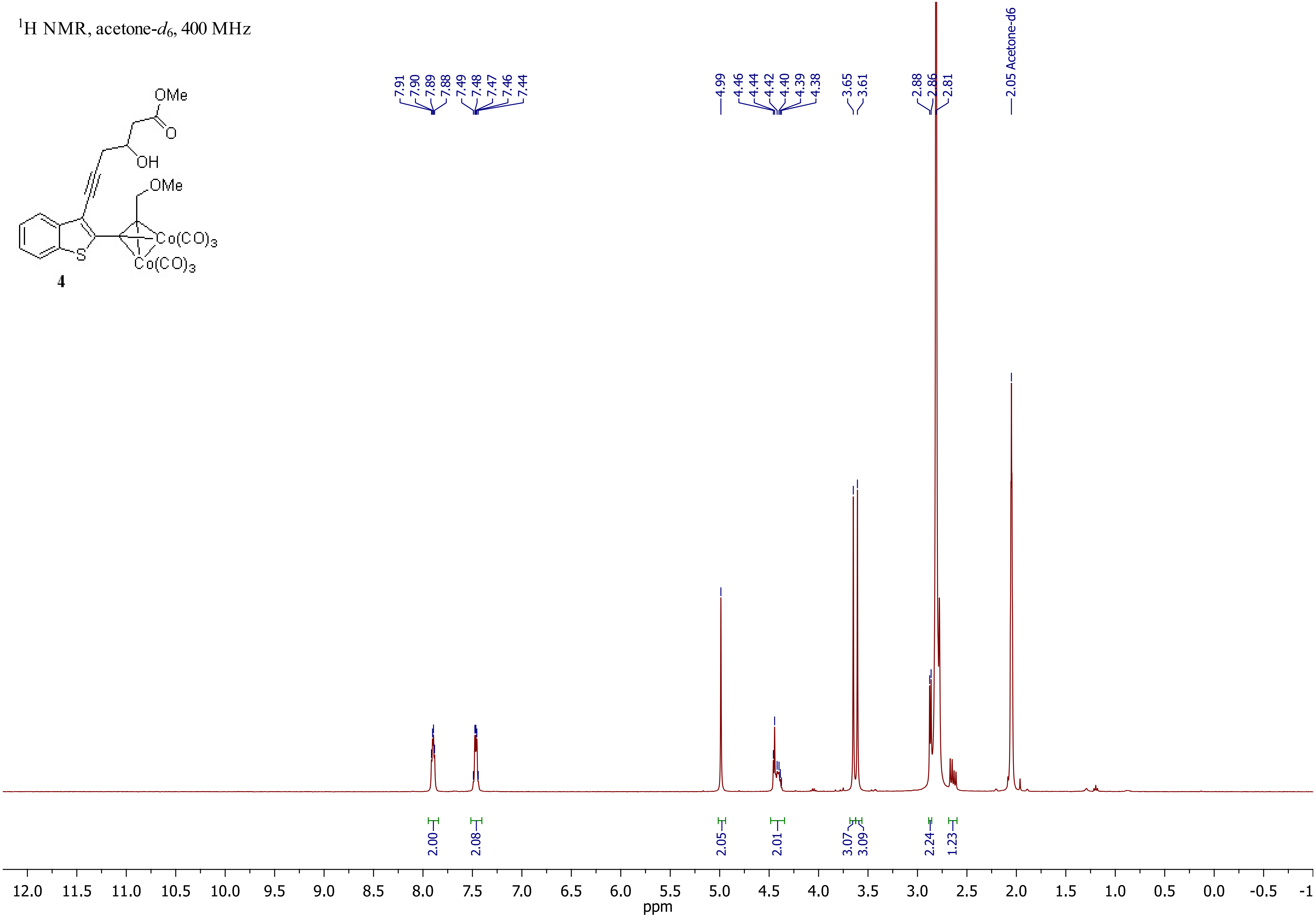

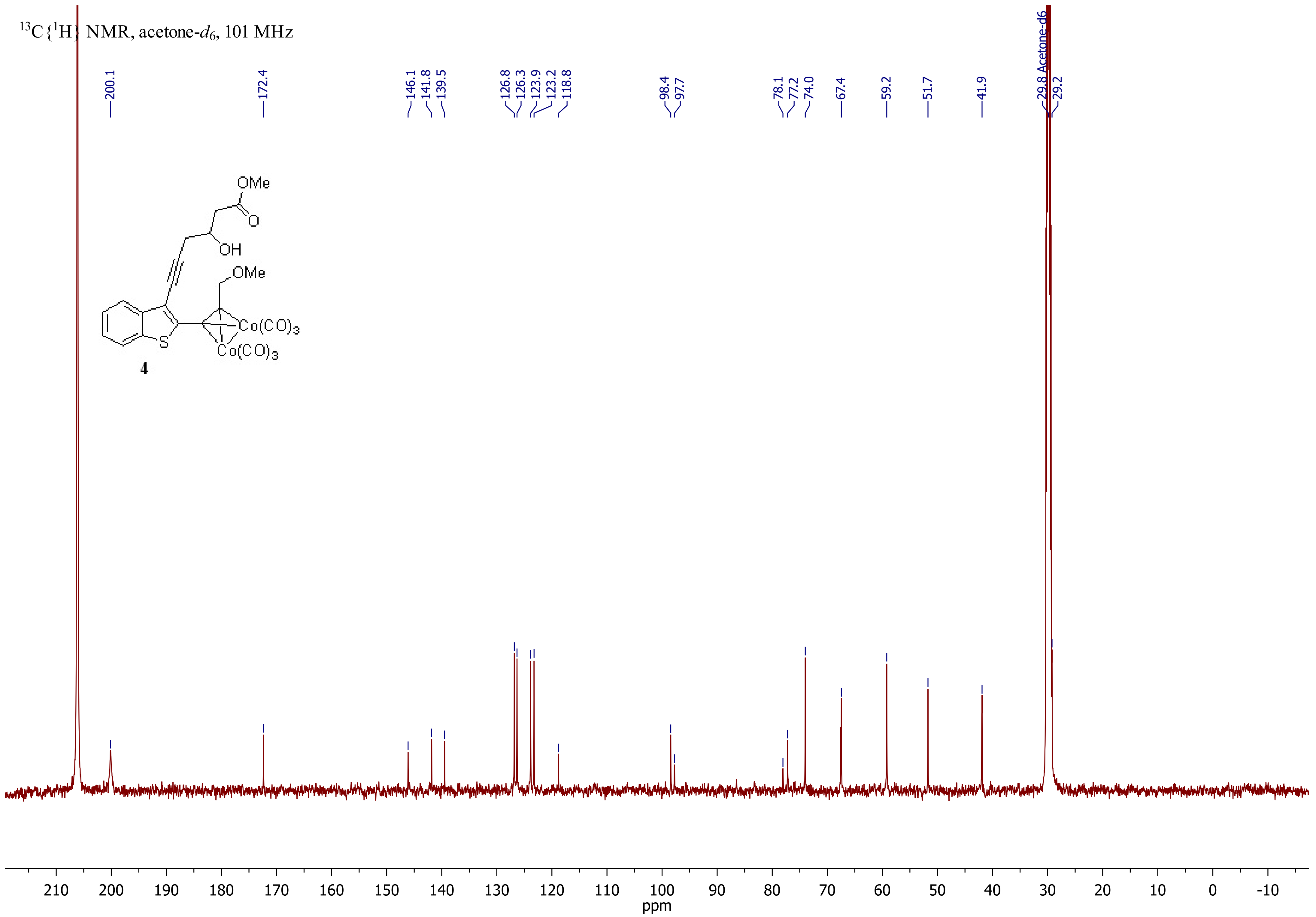

$^{13}C\{^1H\}$ NMR, acetone-d_6, 101 MHz
200.1
172.4
146.1
141.8
139.5
126.8
126.3
123.9
123.2
118.8
98.4
97.7
78.1
77.2
74.0
67.4
59.2
51.7
41.9
29.8 Acetone-d6
29.2
OMe
O
OH
OMe
S
Co(CO)3
Co(CO)3
4
210
200
190
180
170
160
150
140
130
120
110
100
90
80
70
60
50
40
30
20
10
0
-10
ppm

DEPT NMR, acetone-d_6, 101 MHz

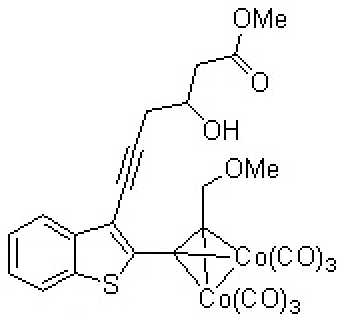

4

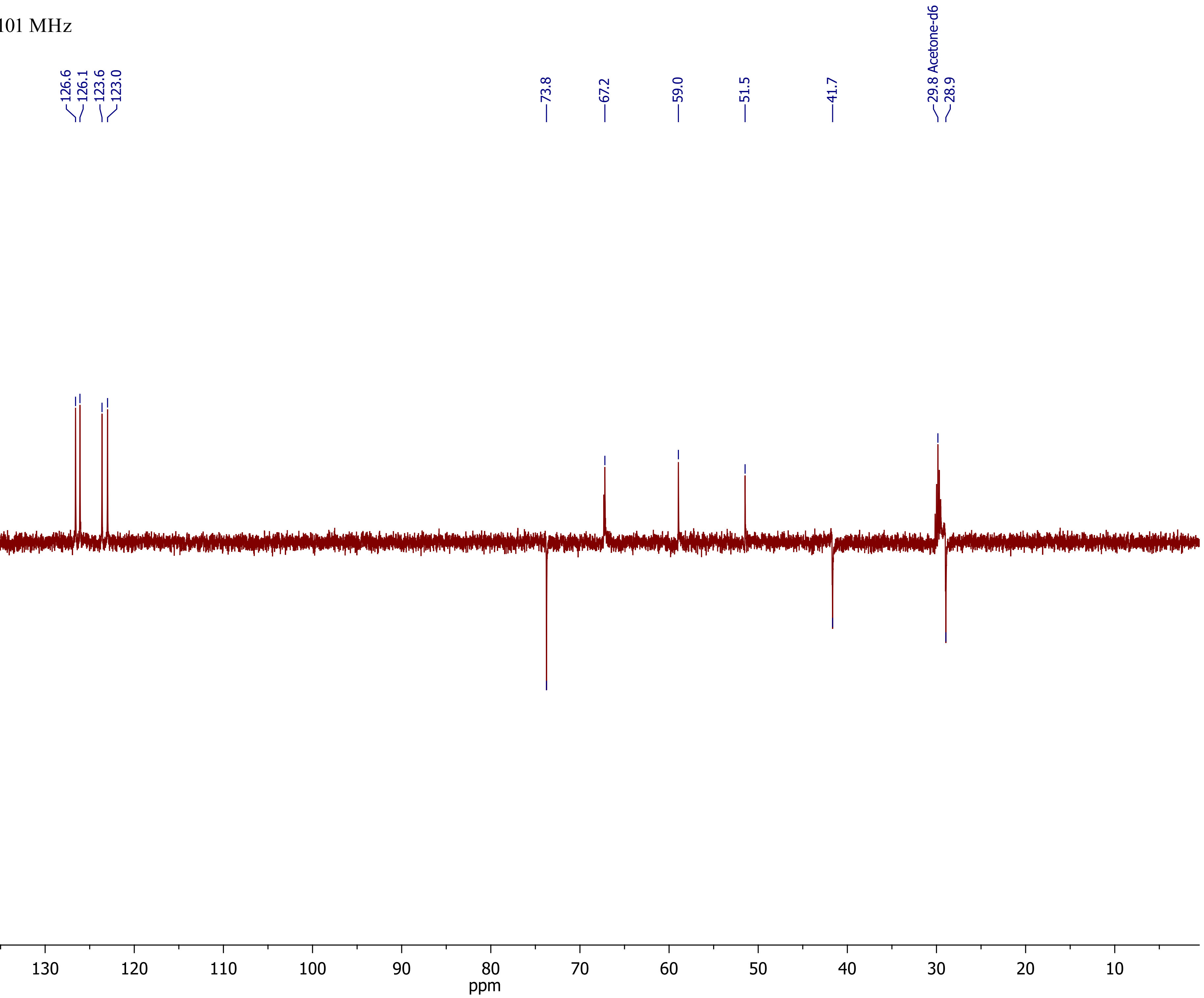

^{1}H NMR, acetone-d_6, 400 MHz

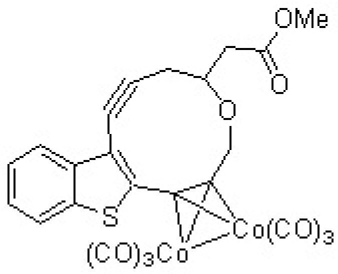

5

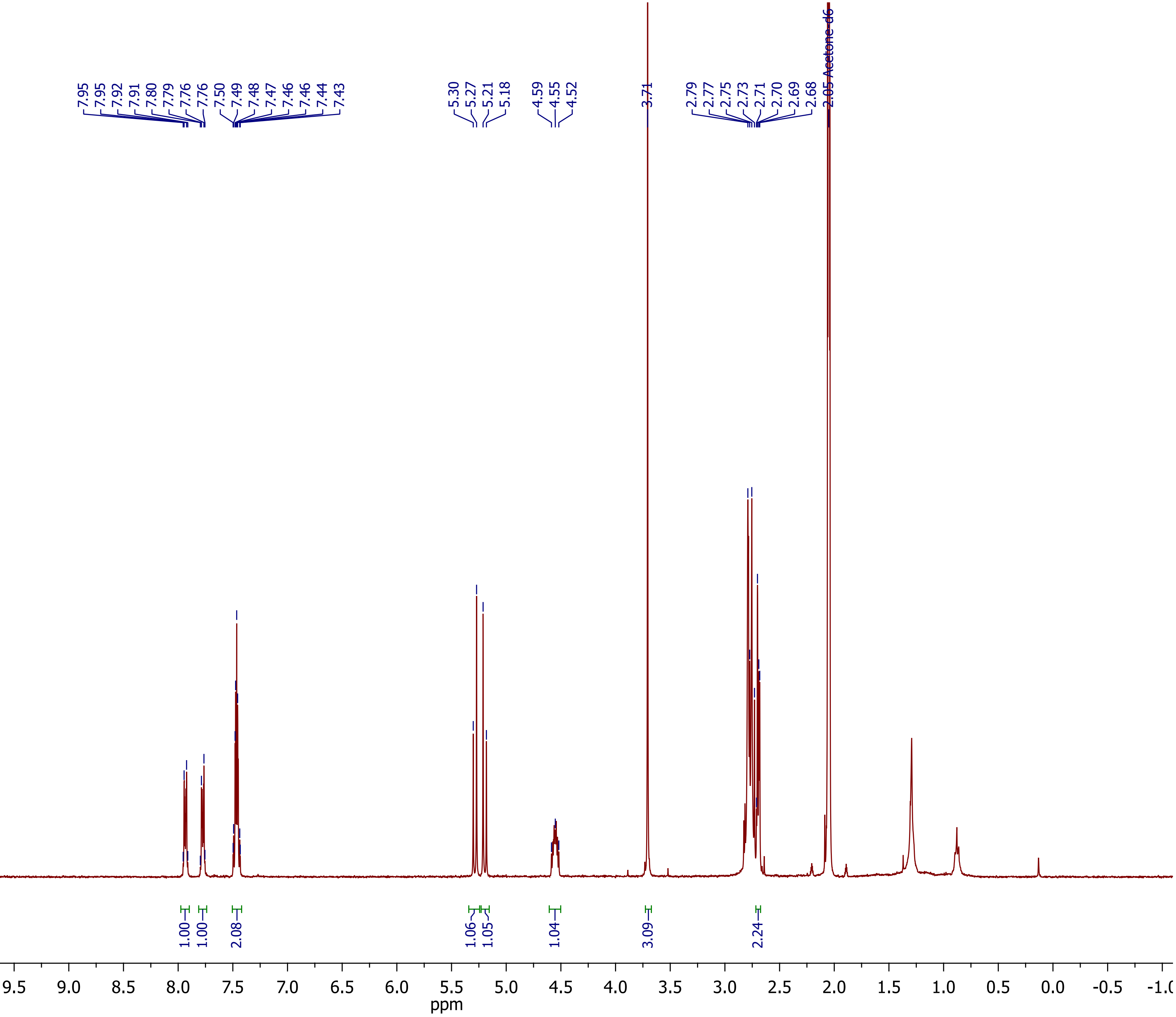

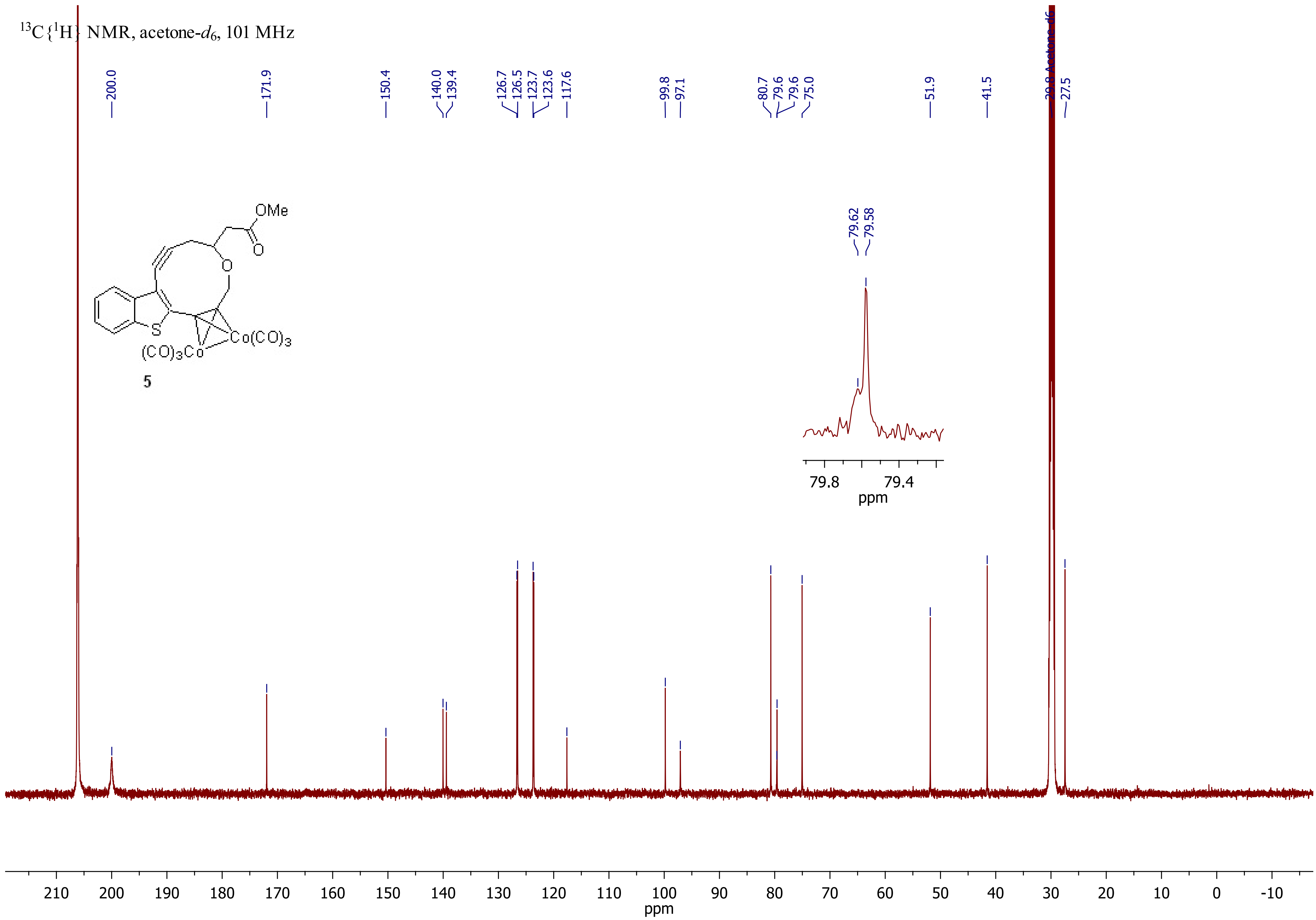

$^{13}C\{^1H\}$ NMR, acetone-d_6, 101 MHz
200.0
171.9
150.4
140.0
139.4
126.7
126.5
123.7
123.6
117.6
99.8
97.1
80.7
79.6
79.6
75.0
51.9
41.5
29.8 Acetone-d6
27.5
OMe
O
O
S
Co(CO)3
(CO)3Co
5
79.62
79.58
79.8
79.4
ppm
210
200
190
180
170
160
150
140
130
120
110
100
90
80
70
60
50
40
30
20
10
0
-10
ppm

DEPT NMR, acetone-d_6, 101 MHz

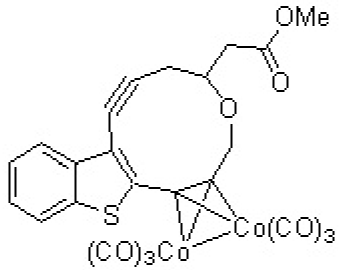

5

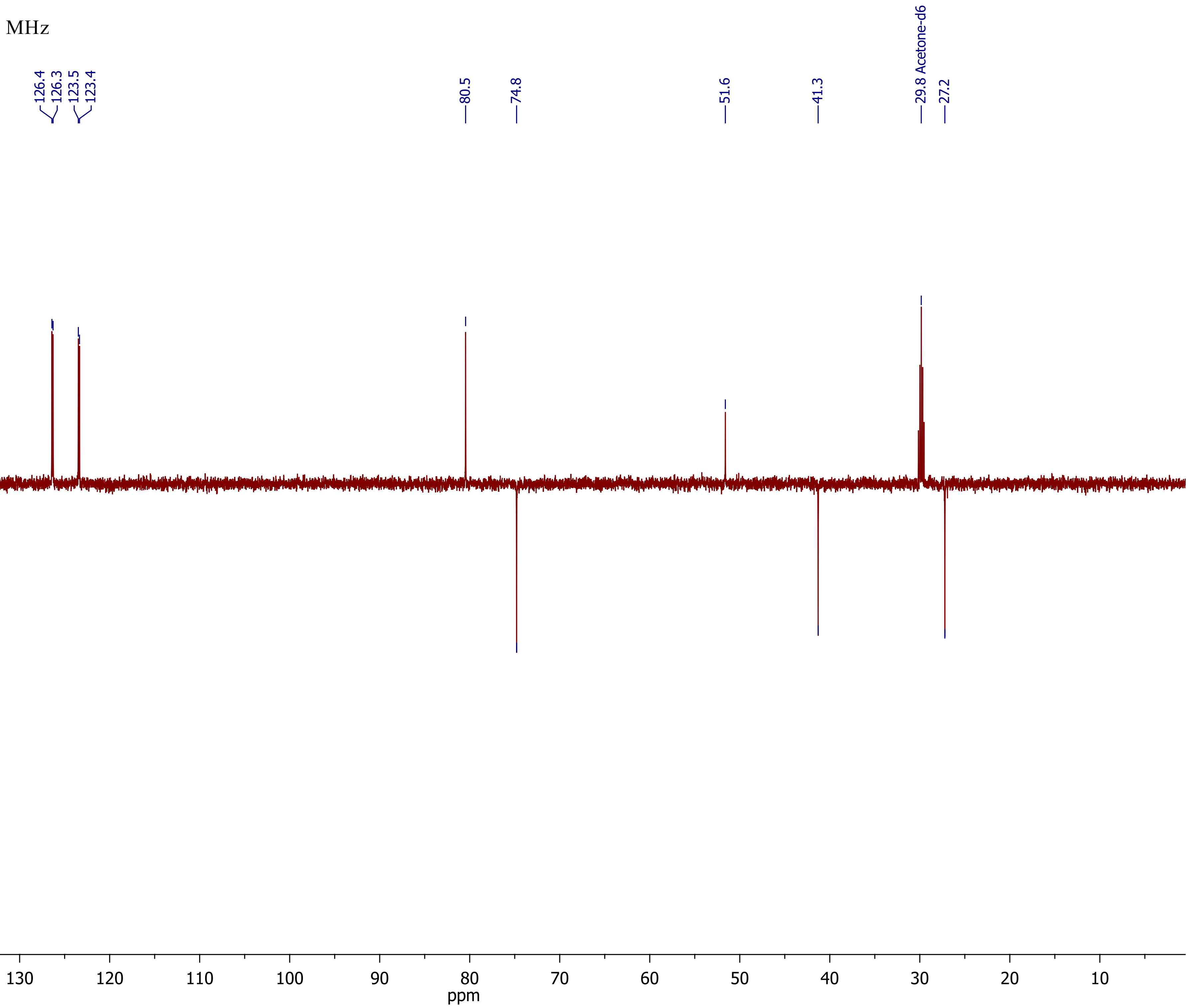

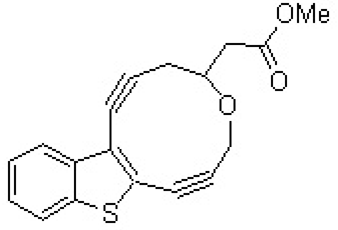
OMe
O
O
S

I

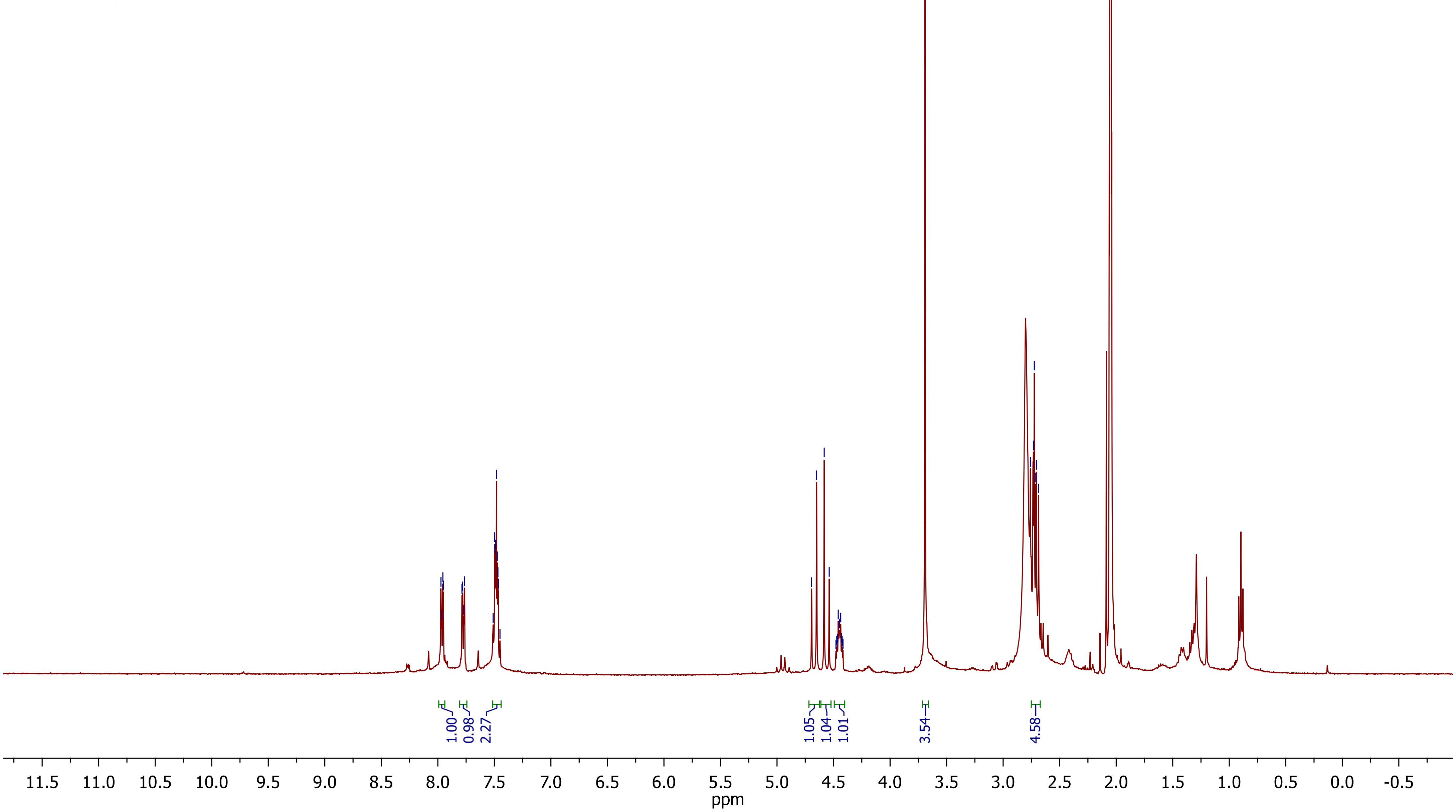
7.97
7.97
7.96
7.95
7.79
7.78
7.77
7.76
7.51
7.50
7.49
7.49
7.48
7.47
7.47
7.46
7.45
4.69
4.65
4.58
4.54
4.48
4.47
4.47
4.46
4.45
4.44
4.43
4.42
4.42
3.69
2.76
2.73
2.72
2.71
2.71
2.69
2.05 Acetone d6
1.00
0.98
2.27
1.05
1.04
1.01
3.54
4.58
11.5
11.0
10.5
10.0
9.5
9.0
8.5
8.0
7.5
7.0
6.5
6.0
5.5
5.0
4.5
4.0
3.5
3.0
2.5
2.0
1.5
1.0
0.5
0.0
-0.5
ppm

^1H NMR, $CDCl_3$, 400 MHz

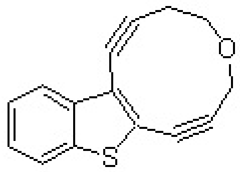

7

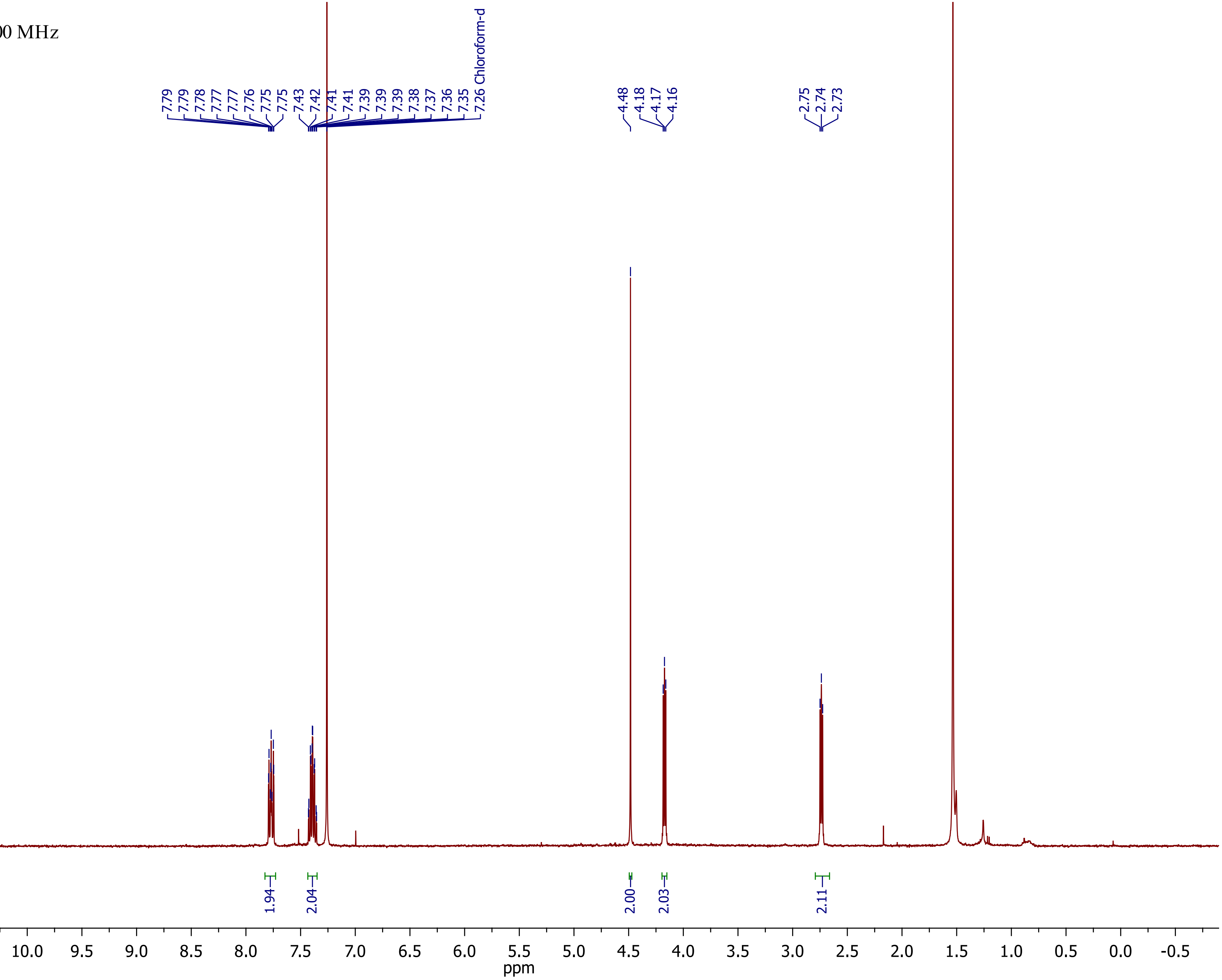

^{1}H NMR, $CDCl_3$, 400 MHz

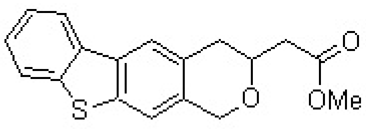

8

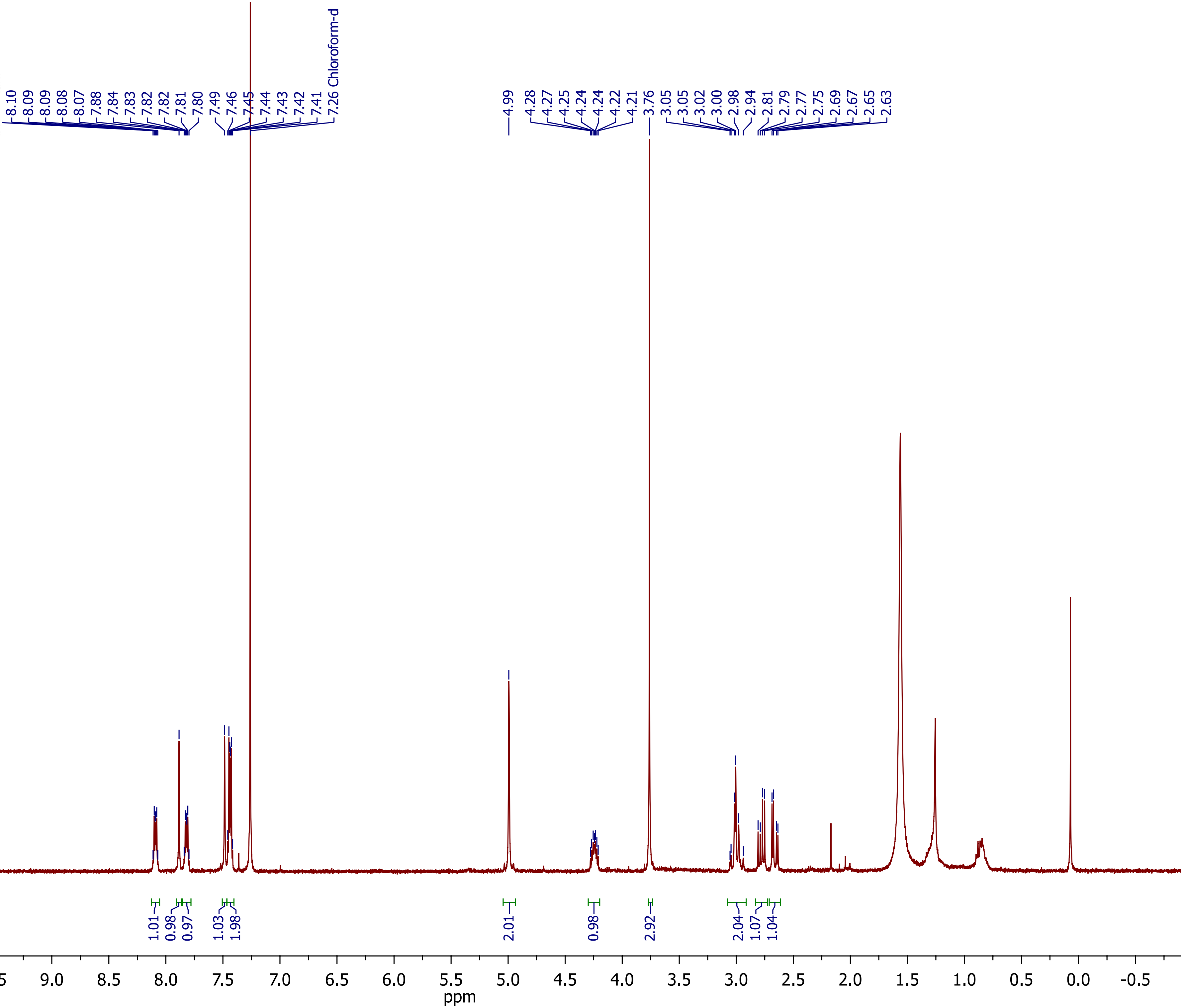

$^{13}C\{^1H\}$ NMR, $CDCl_3$, 101 MHz

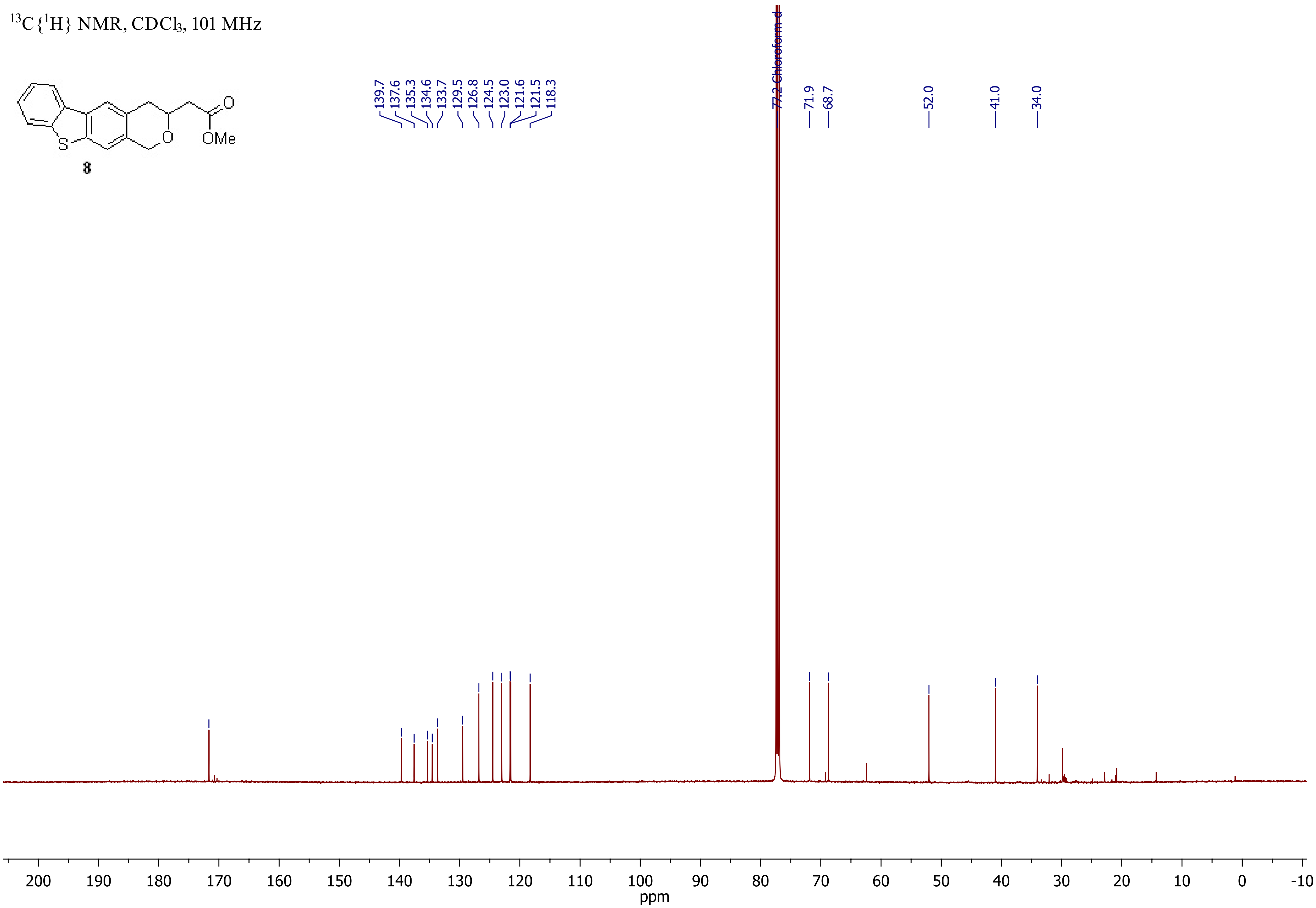

DEPT NMR, $CDCl_3$, 101 MHz

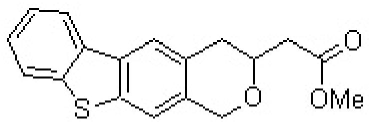

8

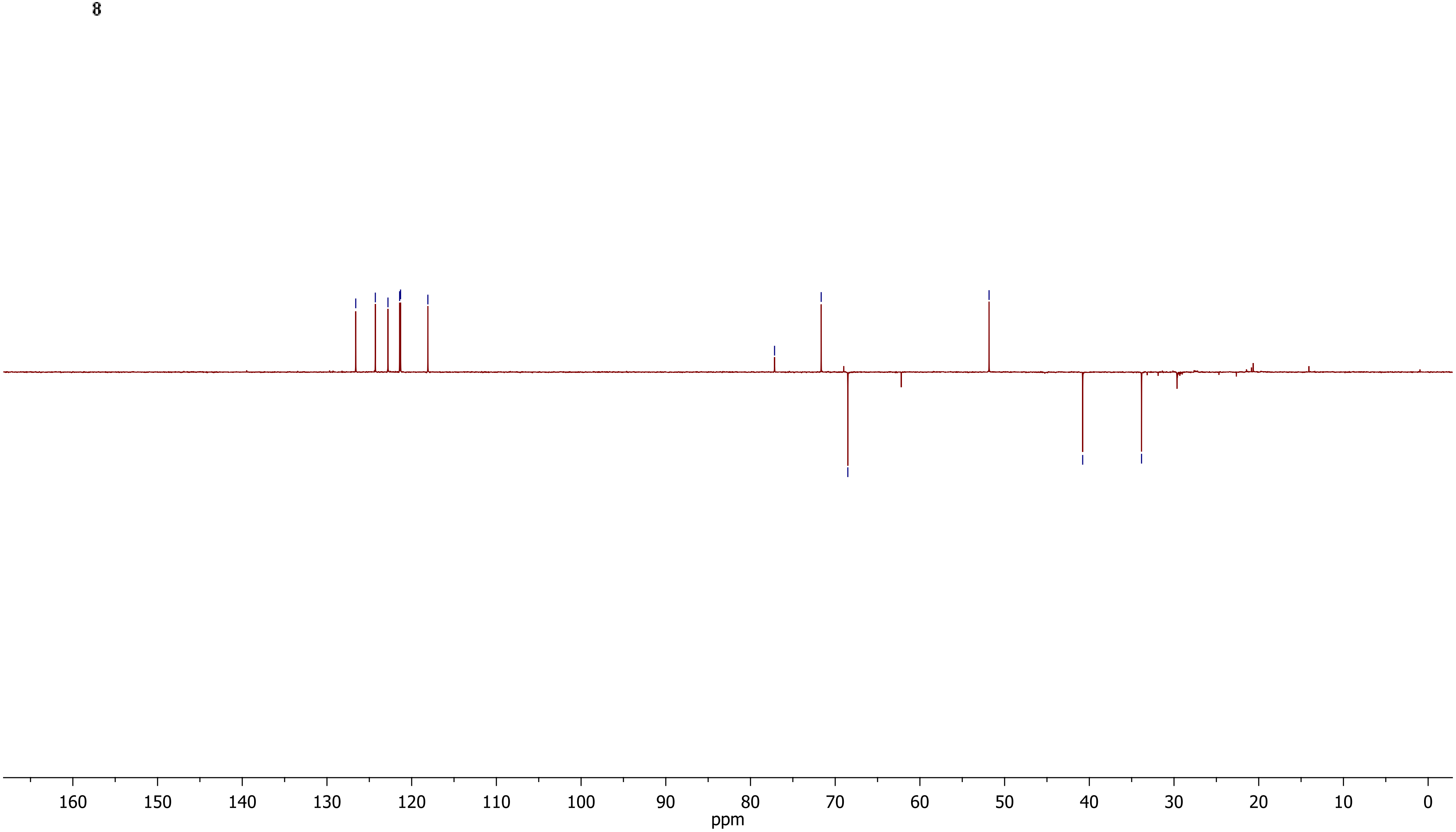

^{1}H NMR, $CDCl_3$, 400 MHz

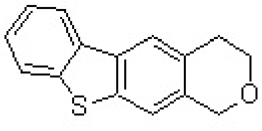

9

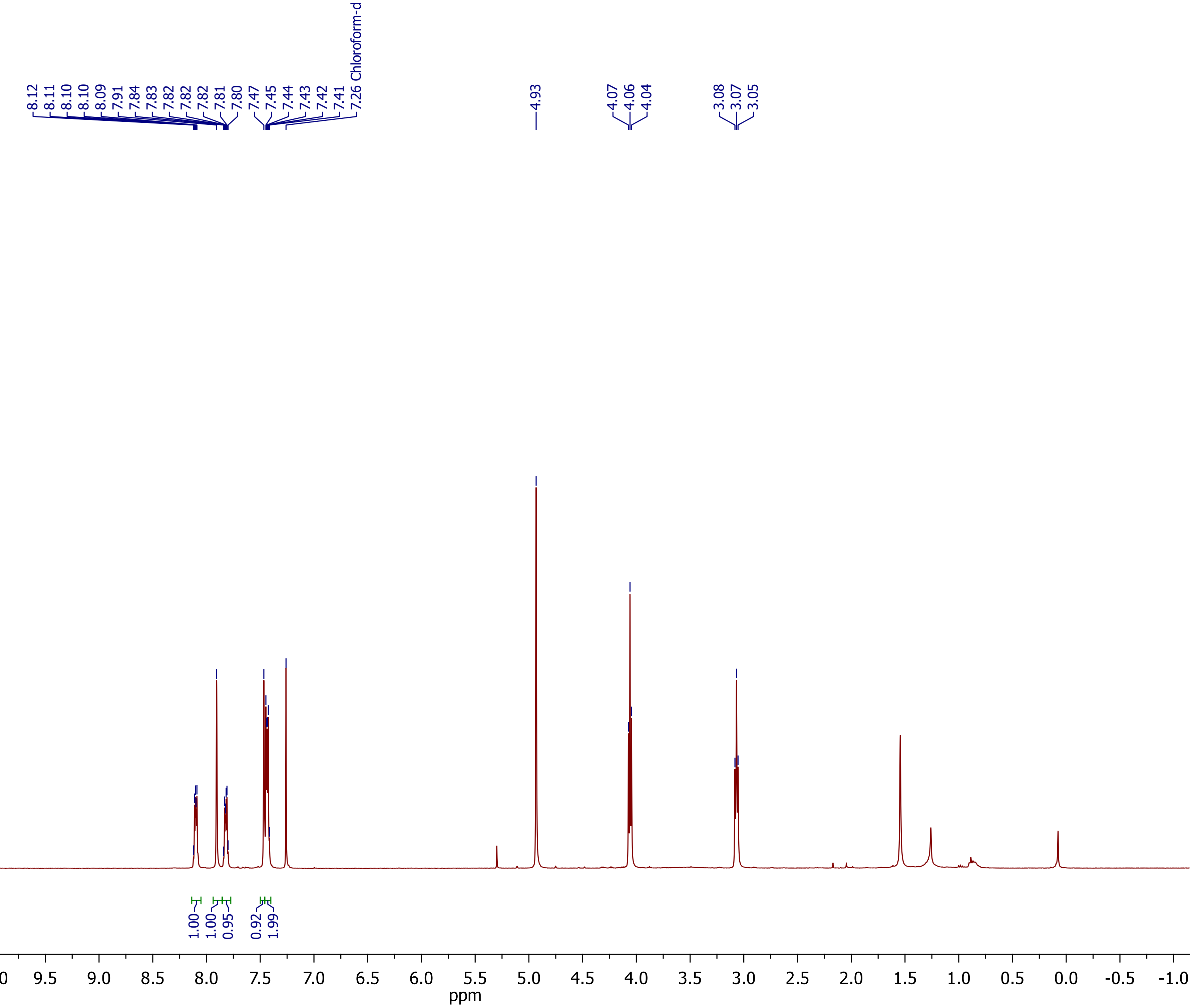

$^{13}C\{^1H\}$ NMR, $CDCl_3$, 101 MHz

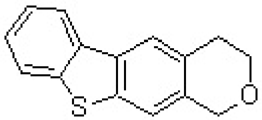

9

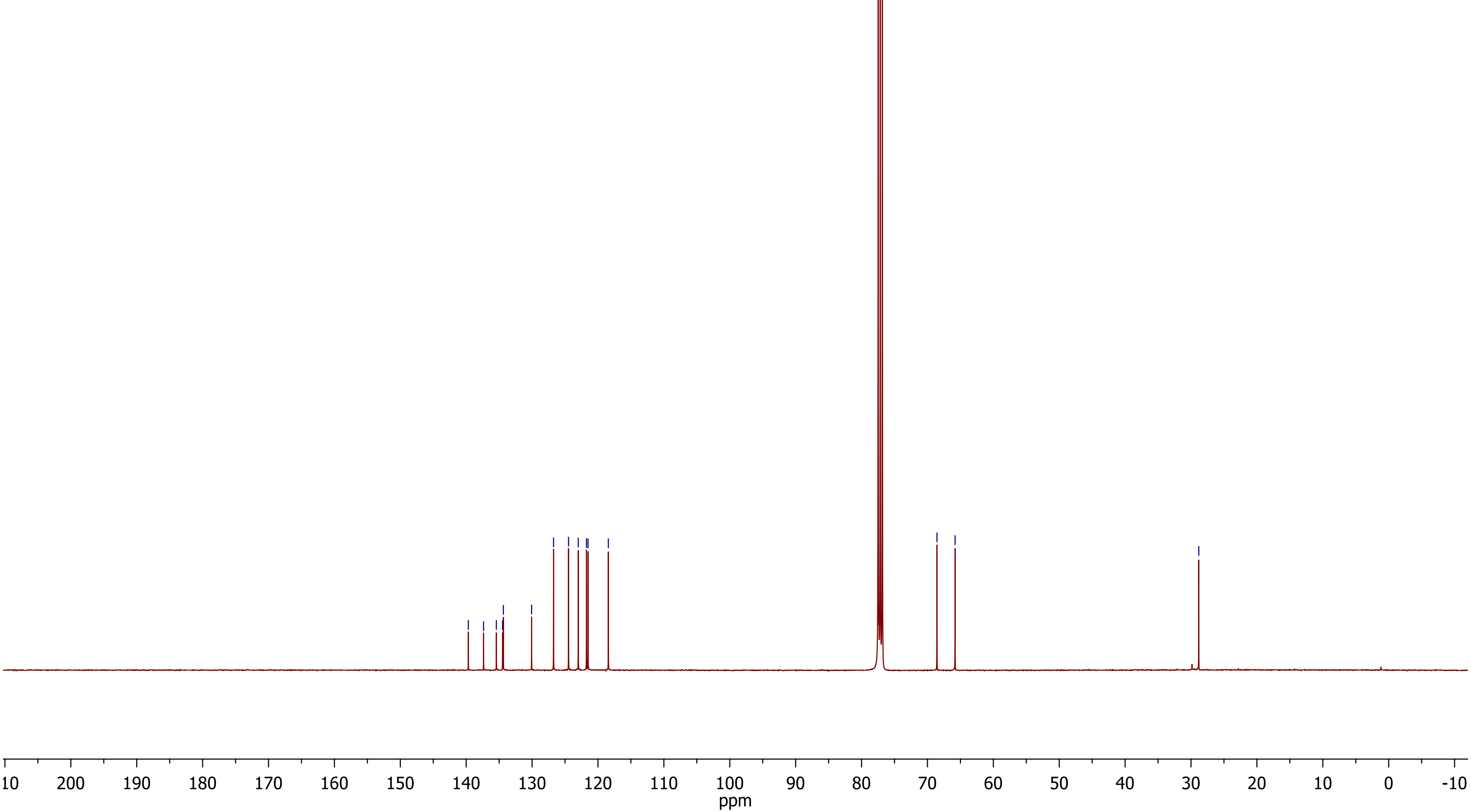

DEPT NMR, $CDCl_3$, 101 MHz

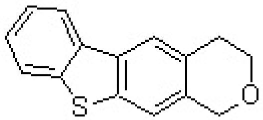

9

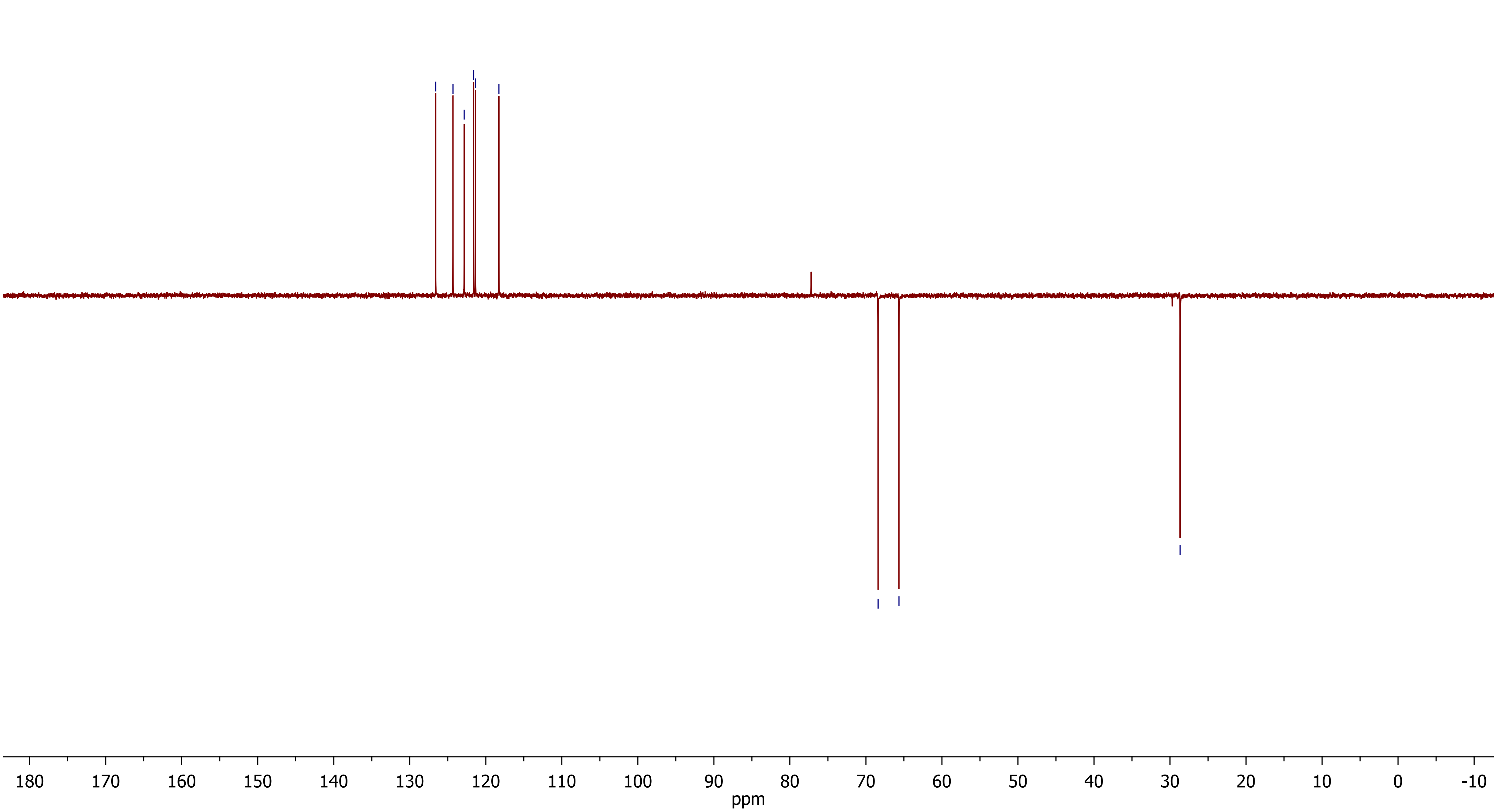

^{1}H NMR, acetone-d_6, 400 MHz

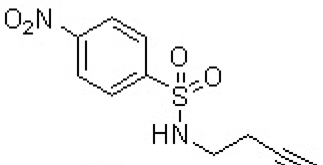

10b

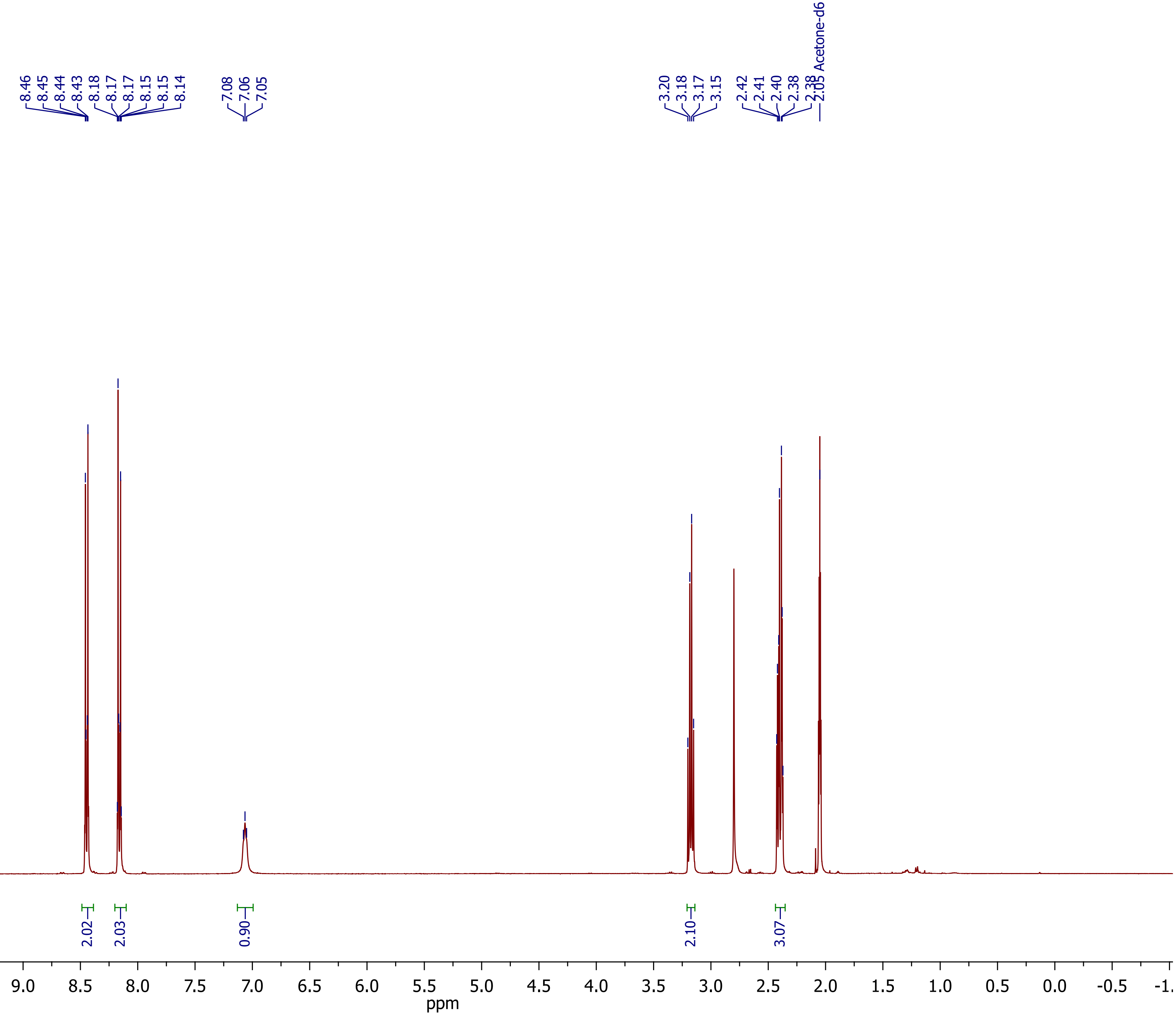

^{1}H NMR, $CDCl_3$, 400 MHz

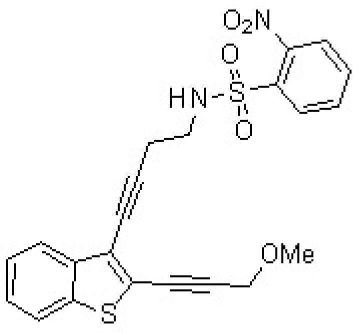

11a

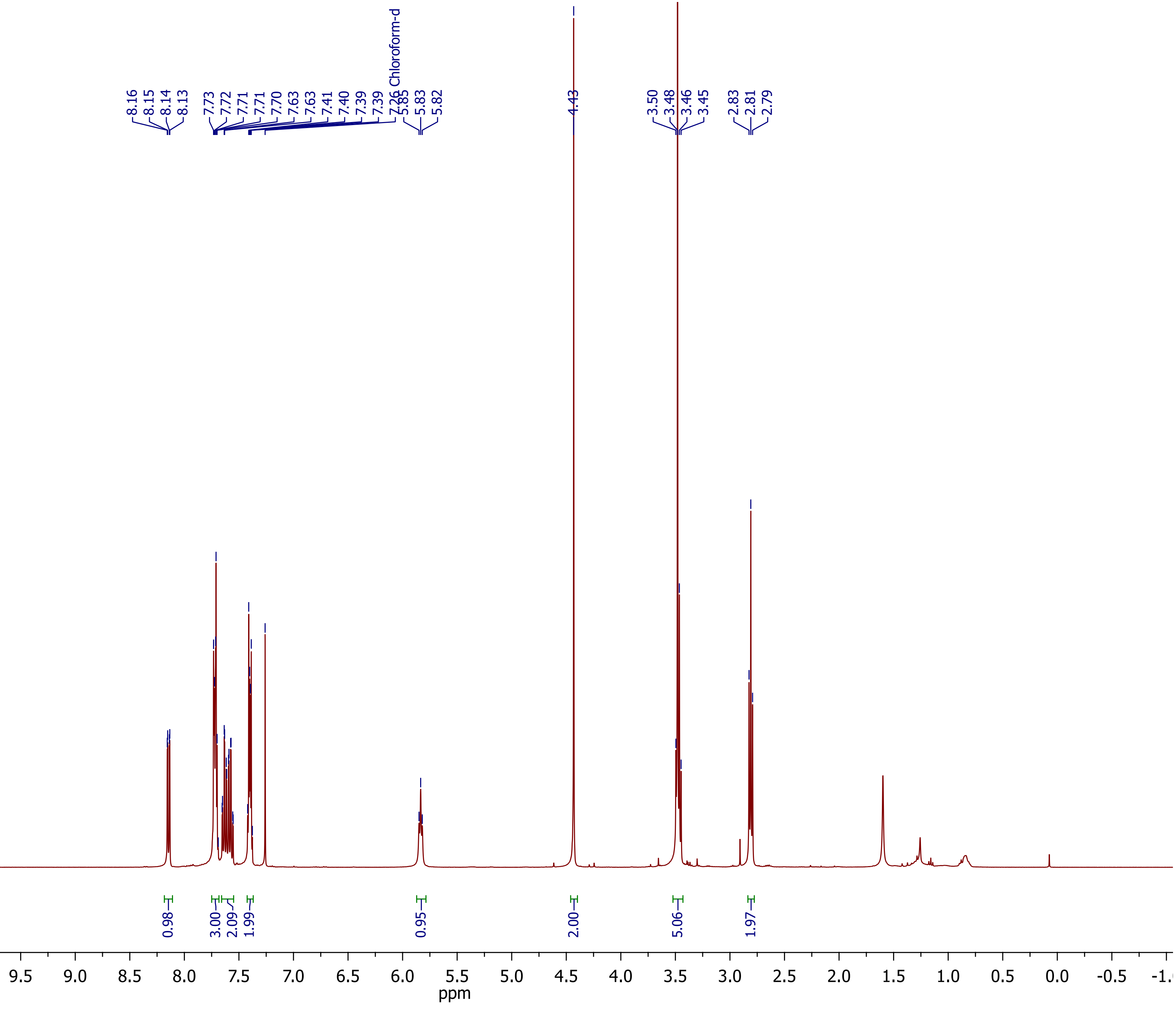

$^{13}C\{^{1}H\}$ NMR, $CDCl_3$, 101 MHz

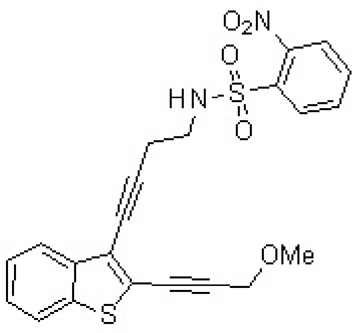

11a

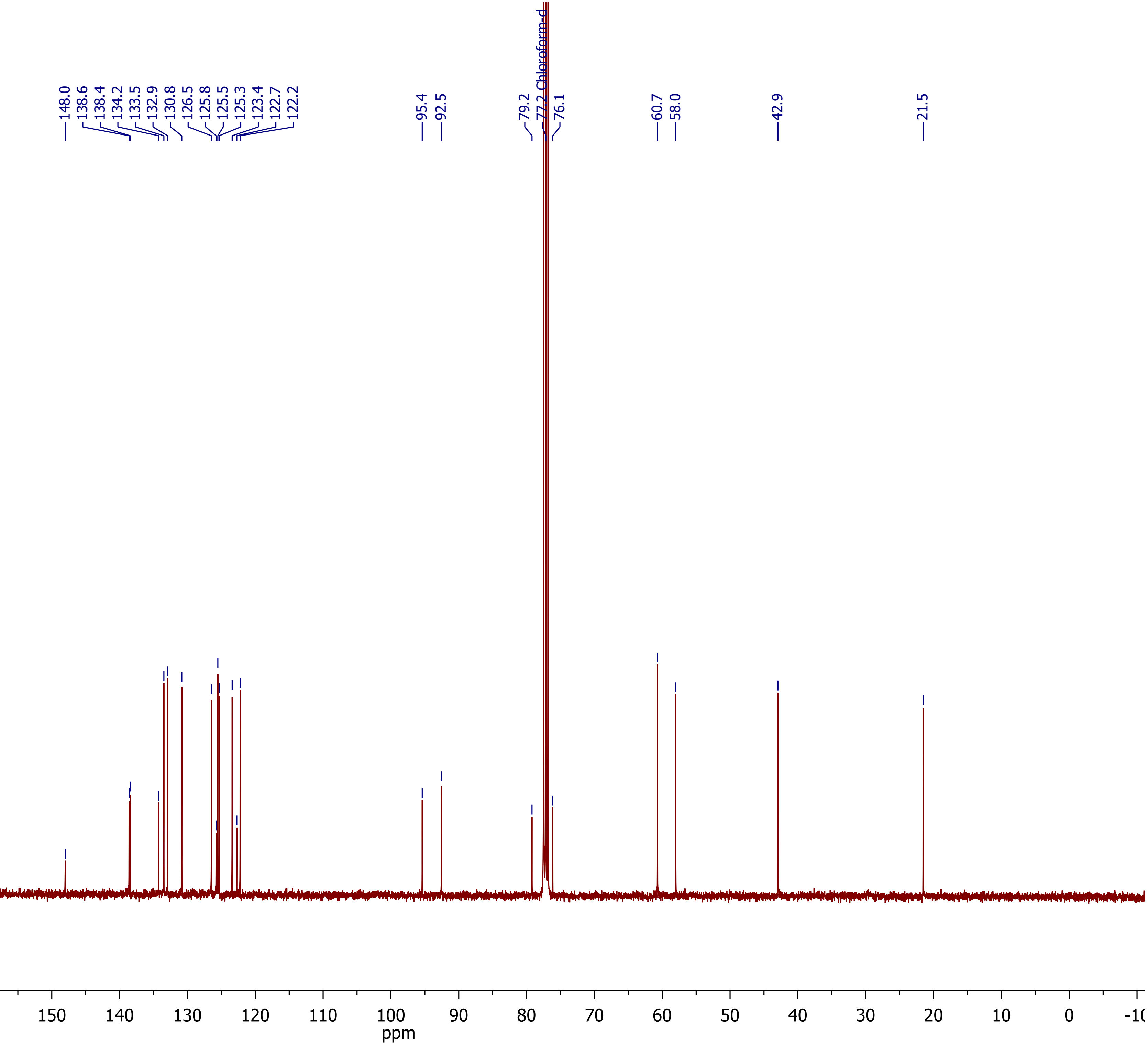

DEPT NMR, $CDCl_3$, 101 MHz

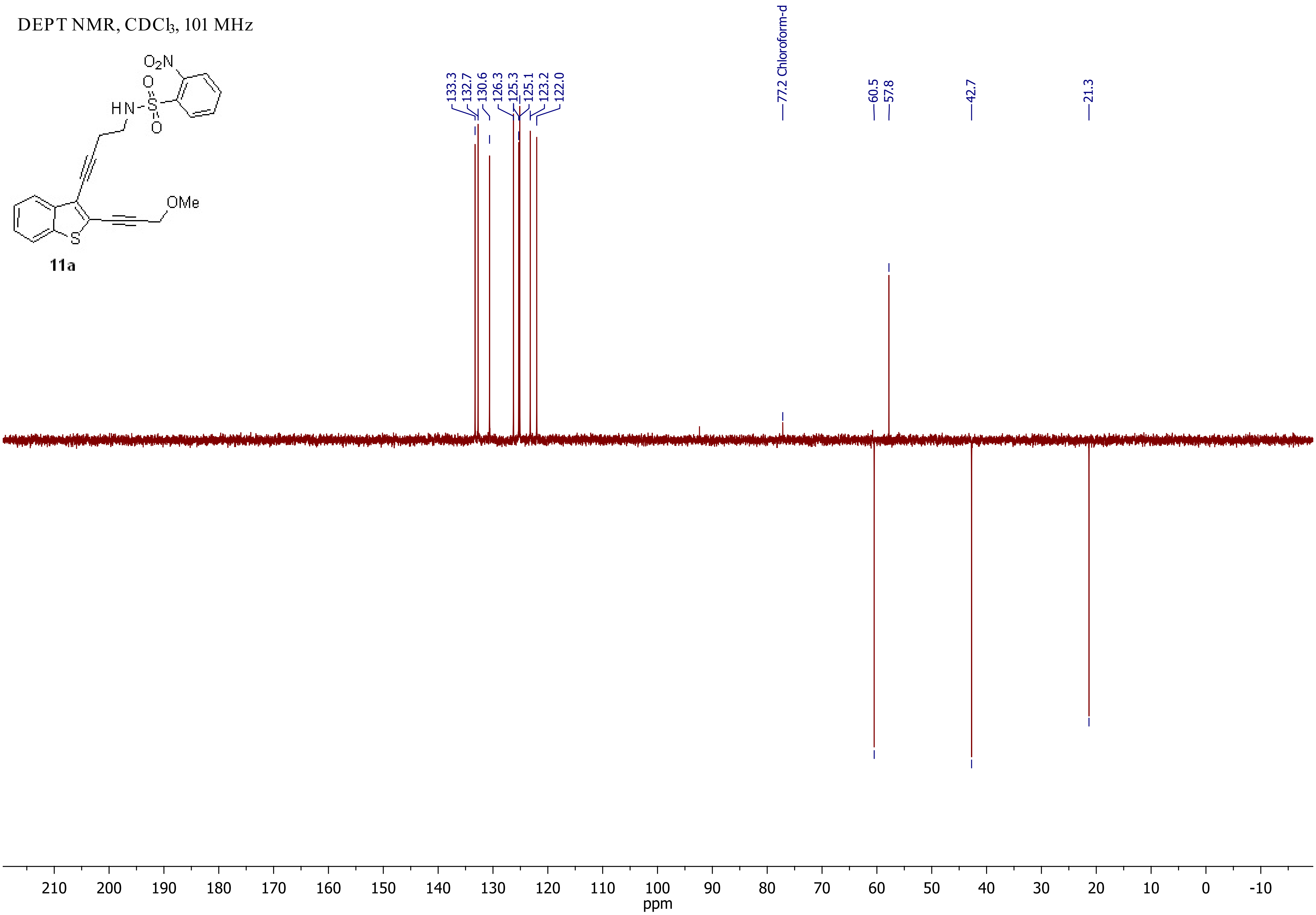

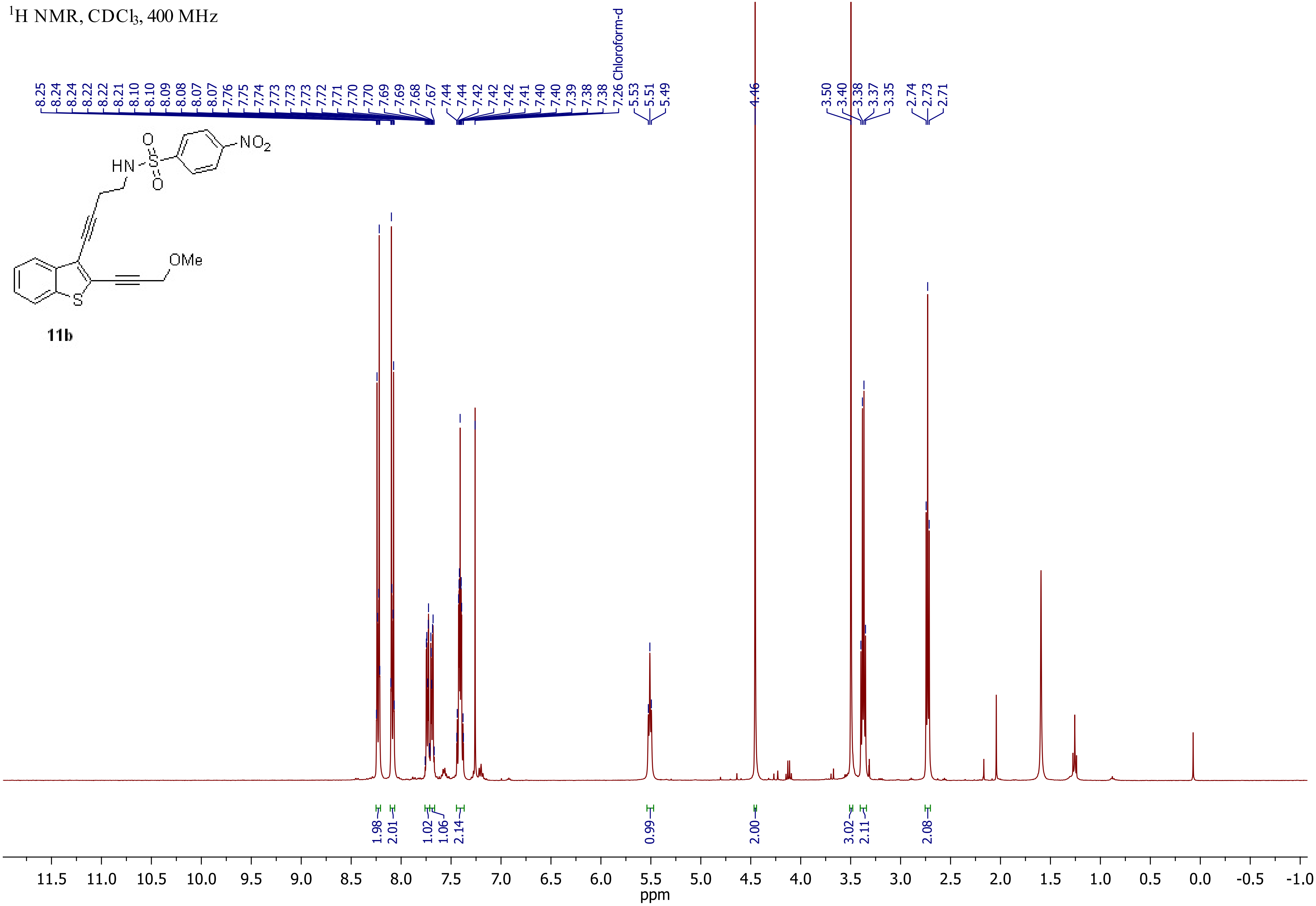

^1H NMR, $CDCl_3$, 400 MHz
11b
Chloroform-d
ppm

$^{13}C\{^1H\}$ NMR, $CDCl_3$, 101 MHz

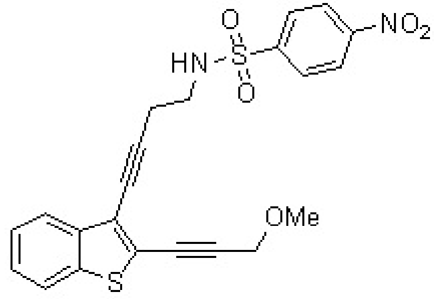

11b

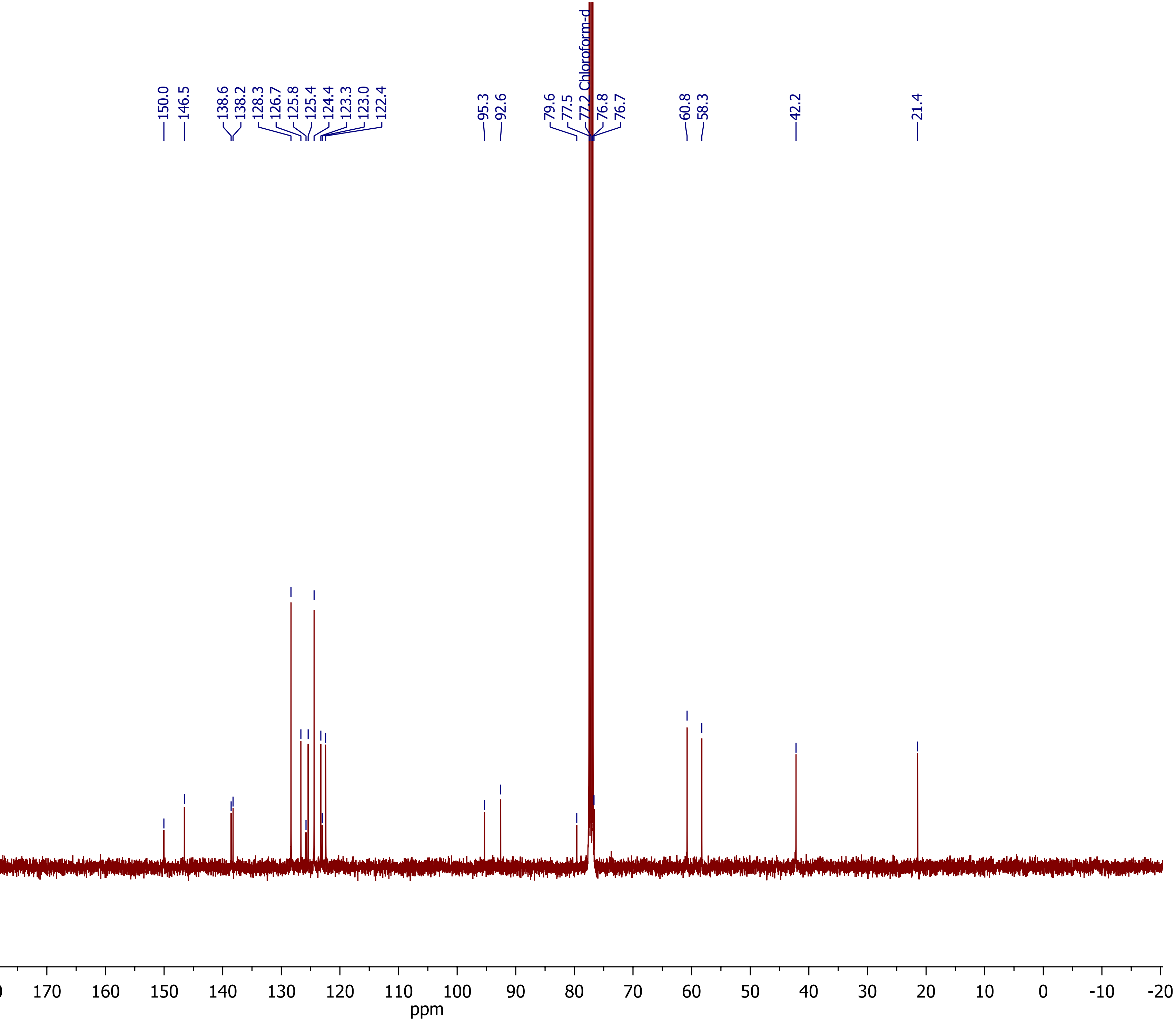

DEPT NMR, $CDCl_3$, 101 MHz

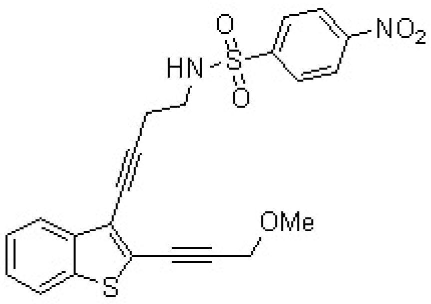

11b

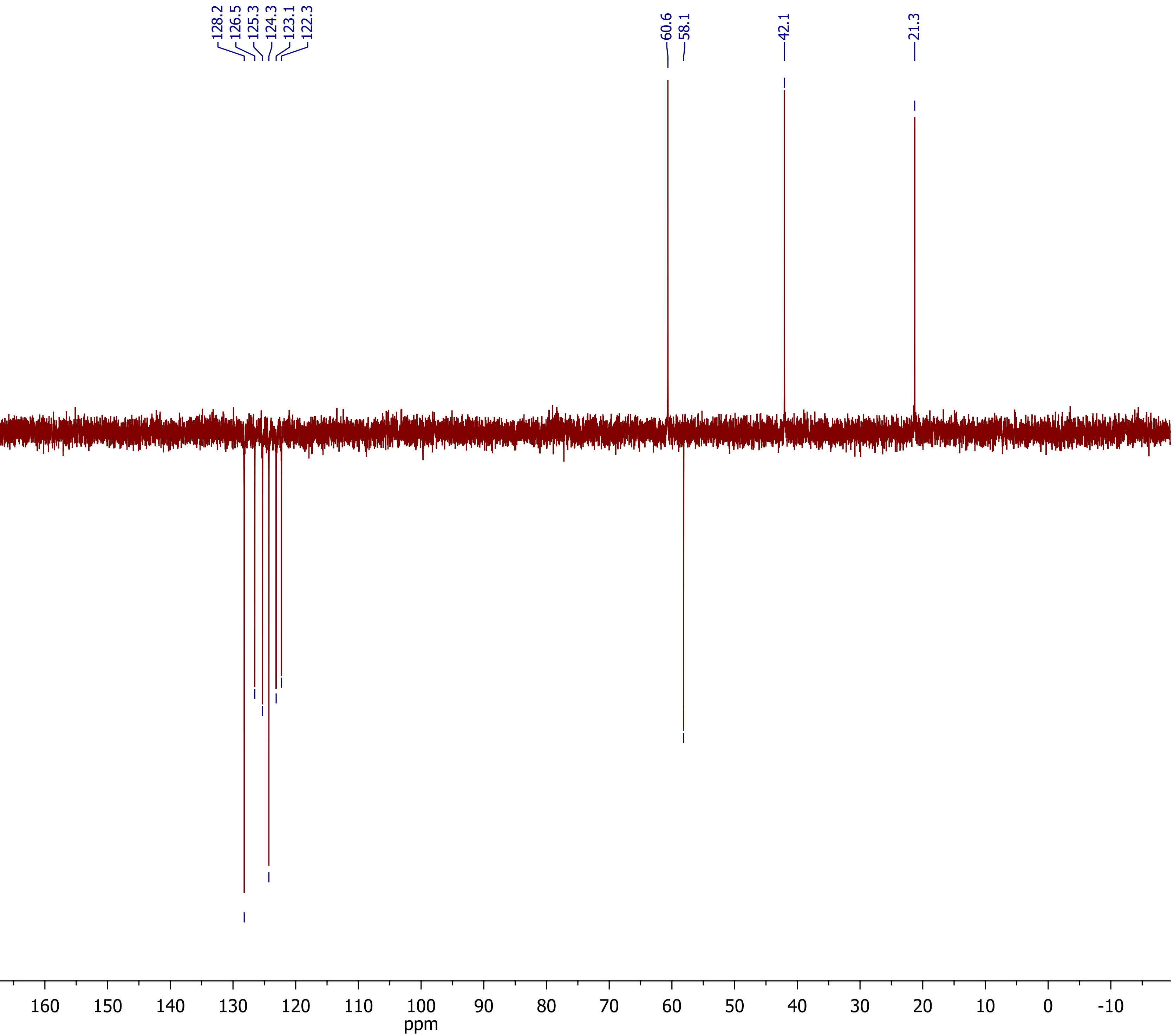

^{1}H NMR, CD_3CN, 400 MHz

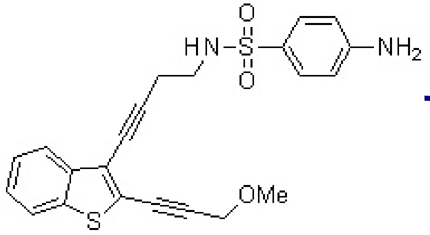

11c

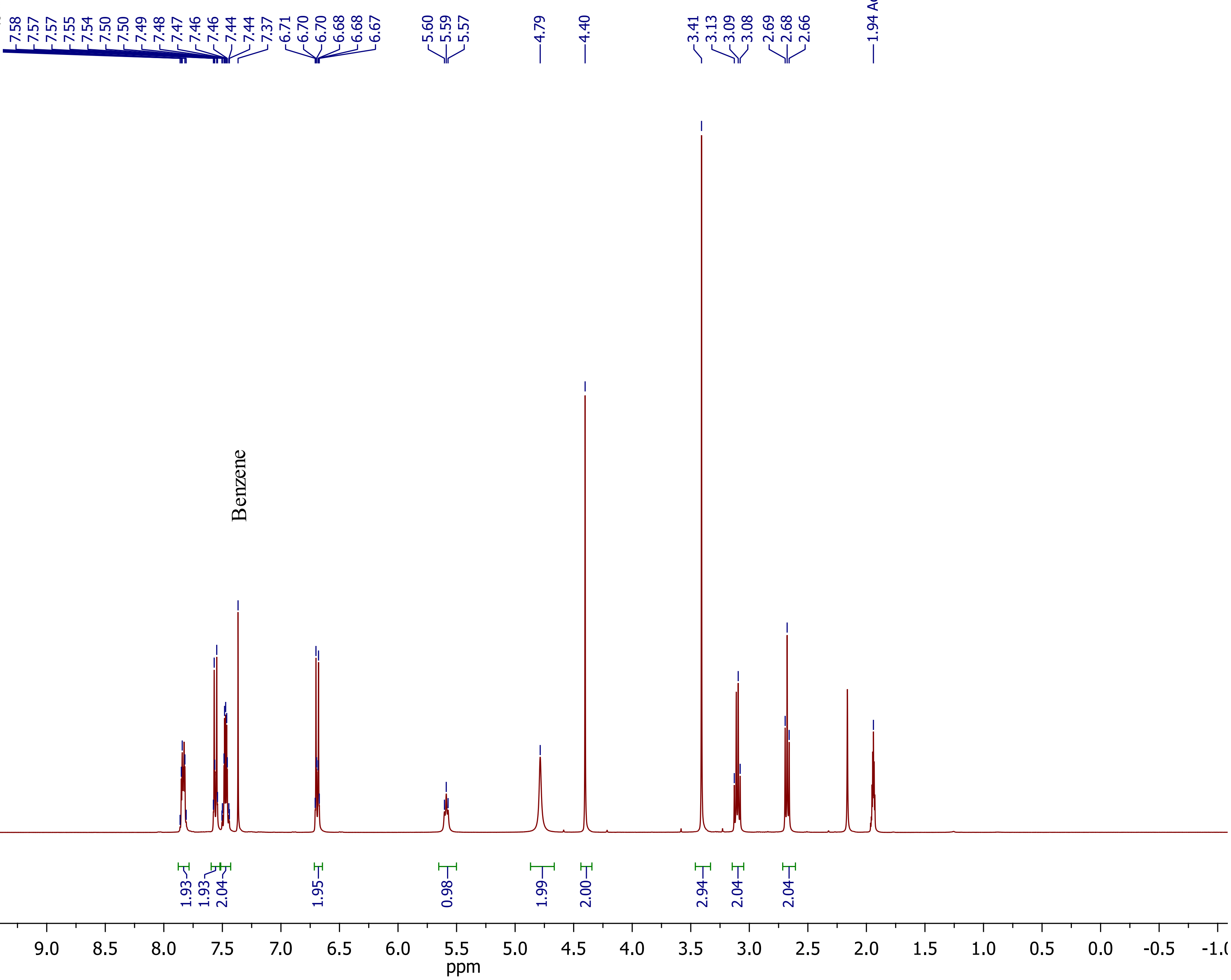

$^{13}C\{^{1}H\}$ NMR, CD_3CN, 101 MHz

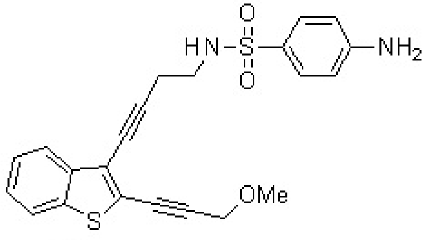

11c

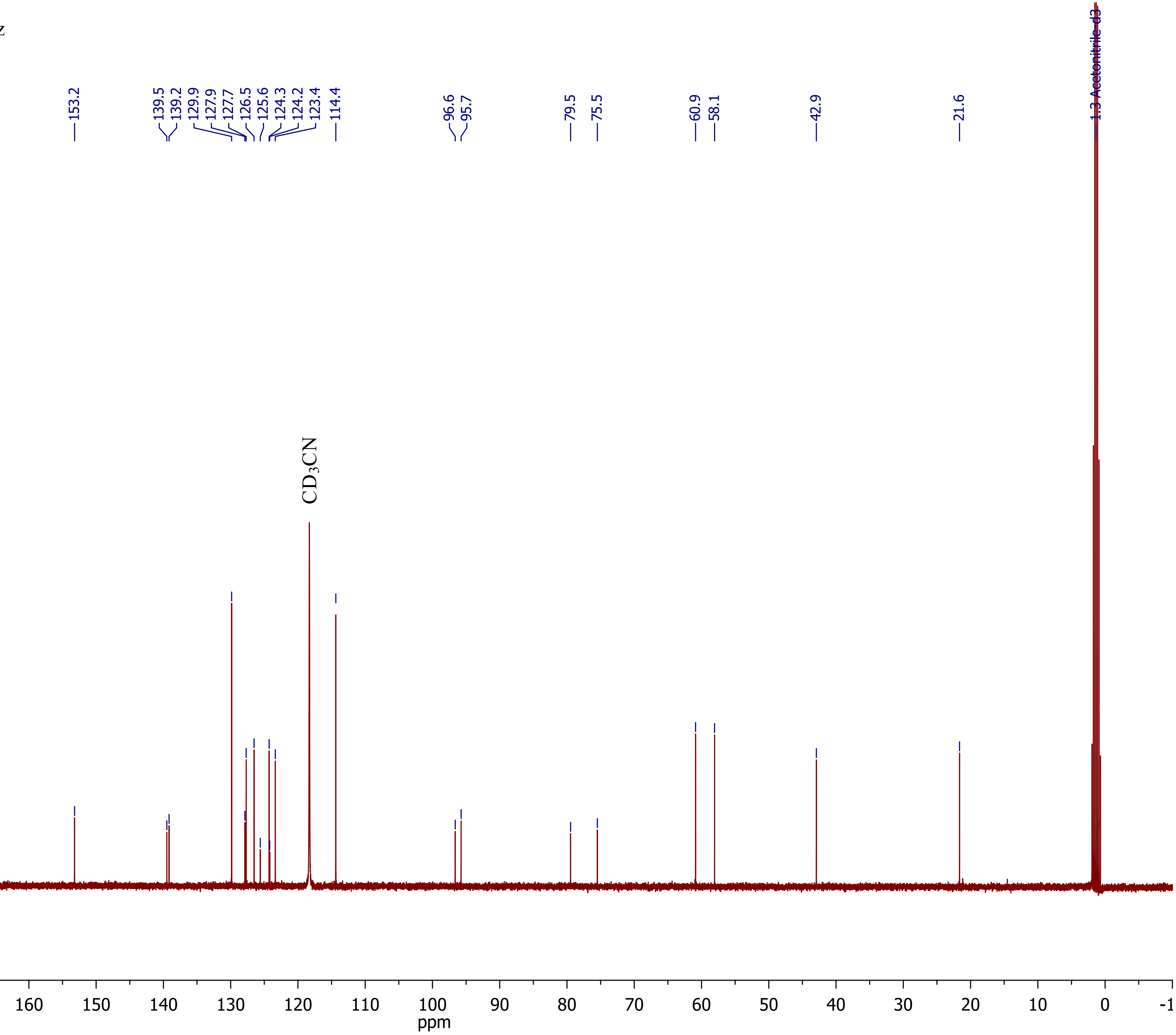

DEPT NMR, CD_3CN, 101 MHz

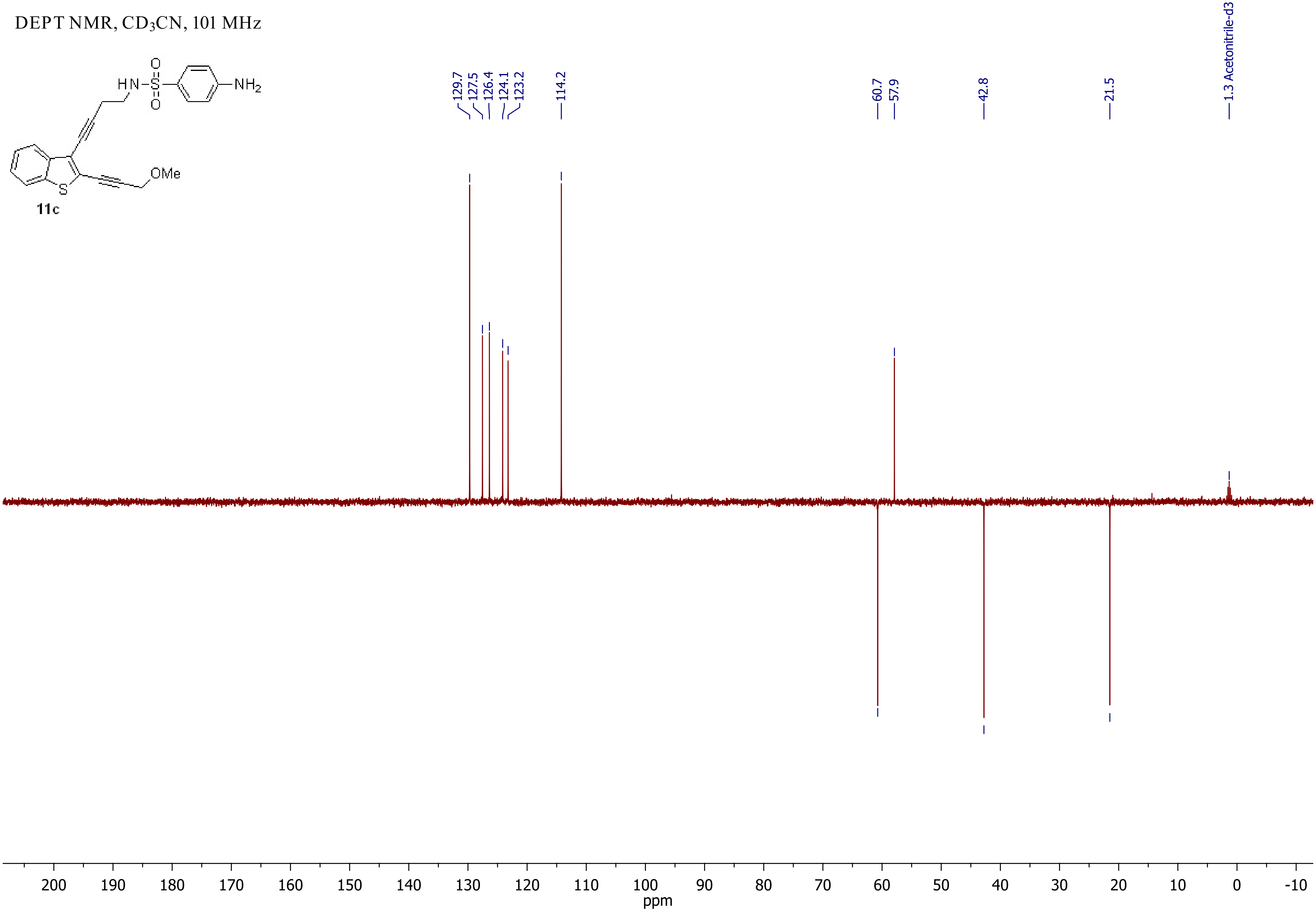

^{1}H NMR, acetone-d_6, 500 MHz

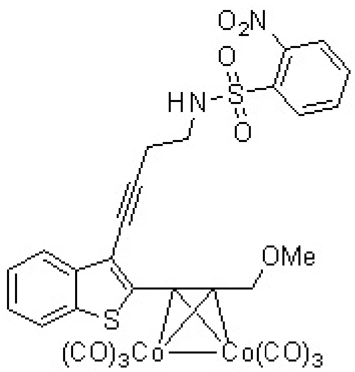

12a

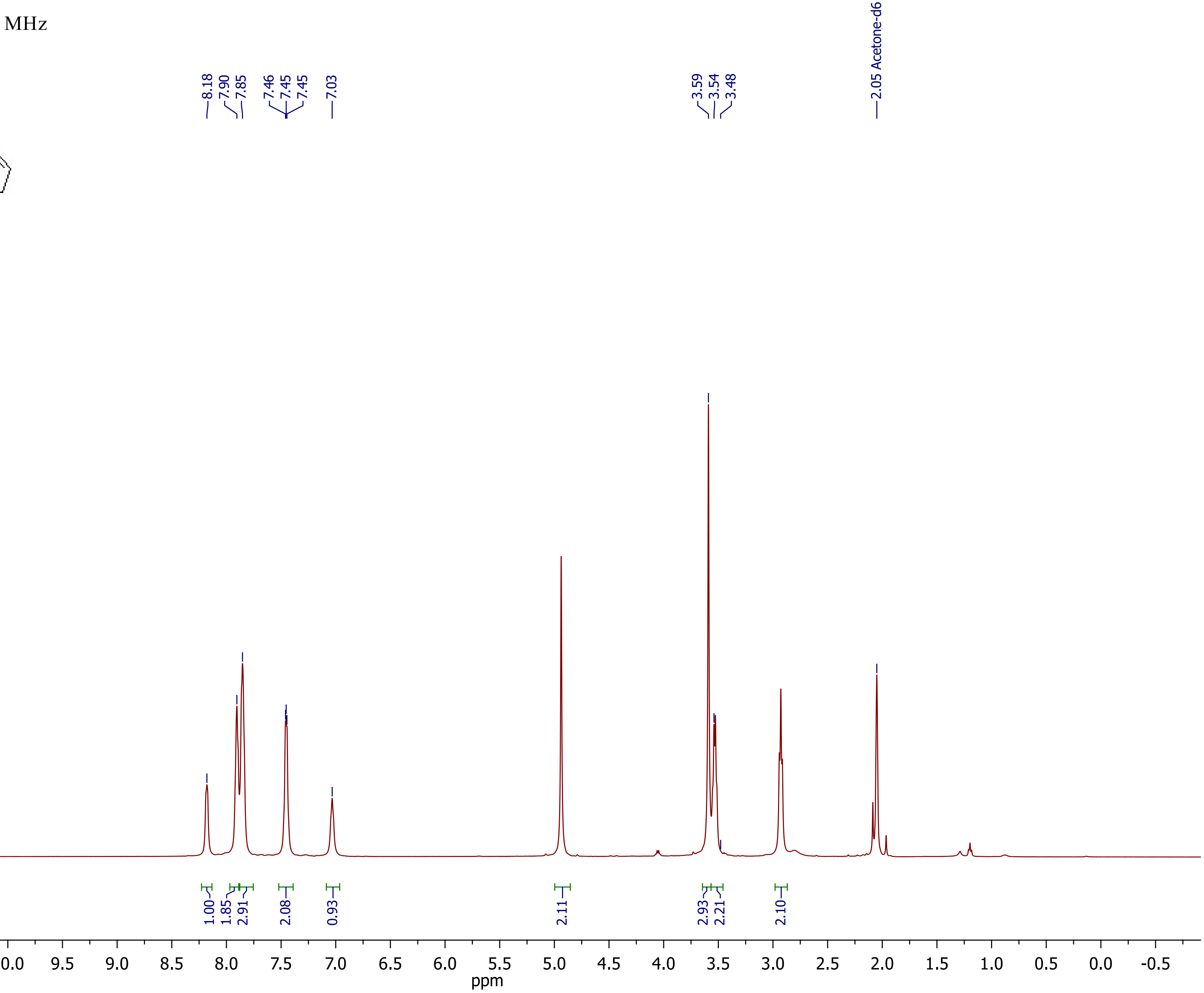

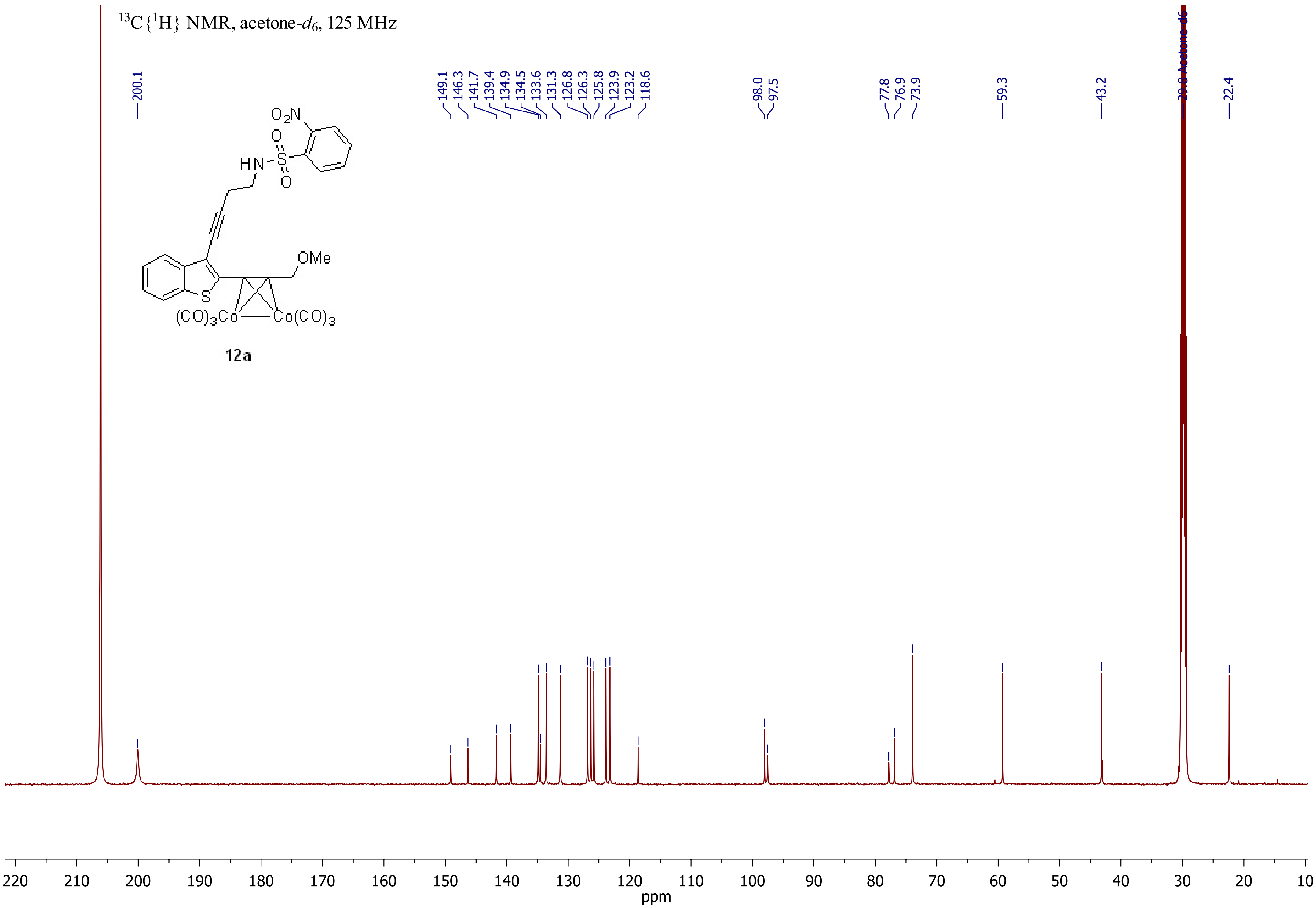

13C{1H} NMR, acetone-d6, 125 MHz
200.1
149.1
146.3
141.7
139.4
134.9
134.5
133.6
131.3
126.8
126.3
125.8
123.9
123.2
118.6
98.0
97.5
77.8
76.9
73.9
59.3
43.2
29.8 Acetone-d6
22.4
O2N
HN
S
O
OMe
(CO)3Co
Co(CO)3
12a
220
210
200
190
180
170
160
150
140
130
120
110
100
90
80
70
60
50
40
30
20
10
ppm

DEPT NMR, acetone-d_6, 125 MHz

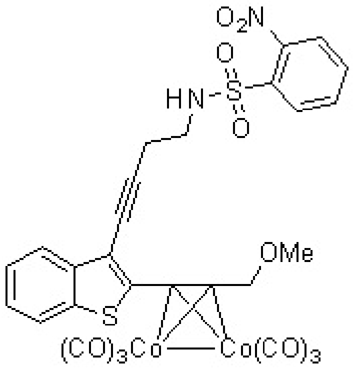

12a

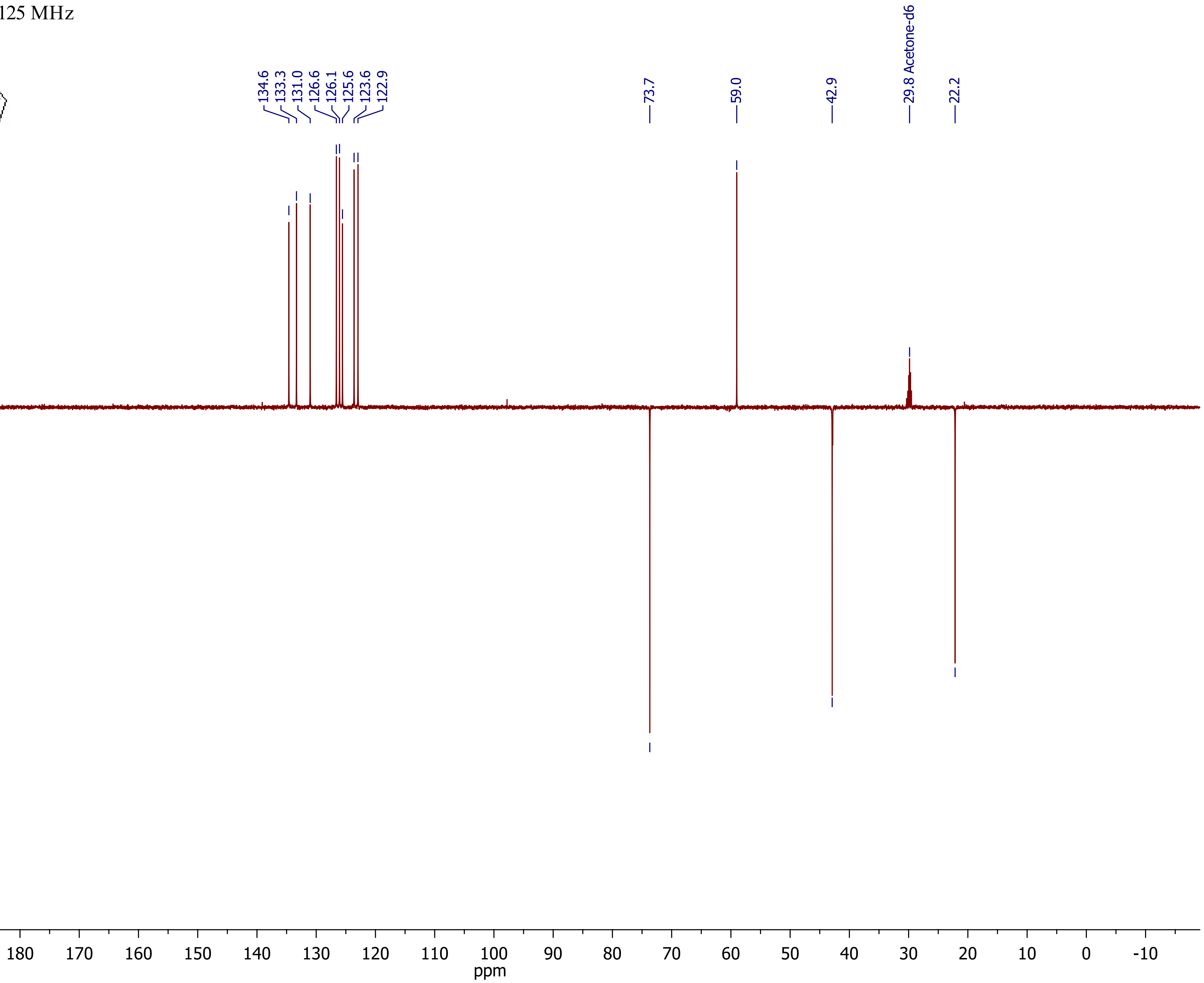

^{1}H NMR, CD_3CN, 400 MHz

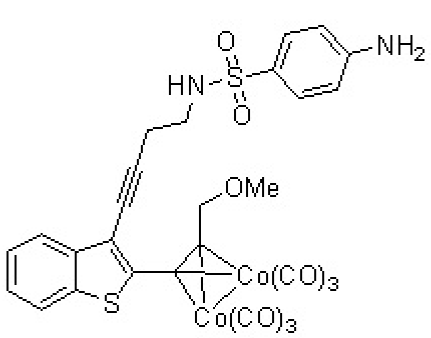

12b

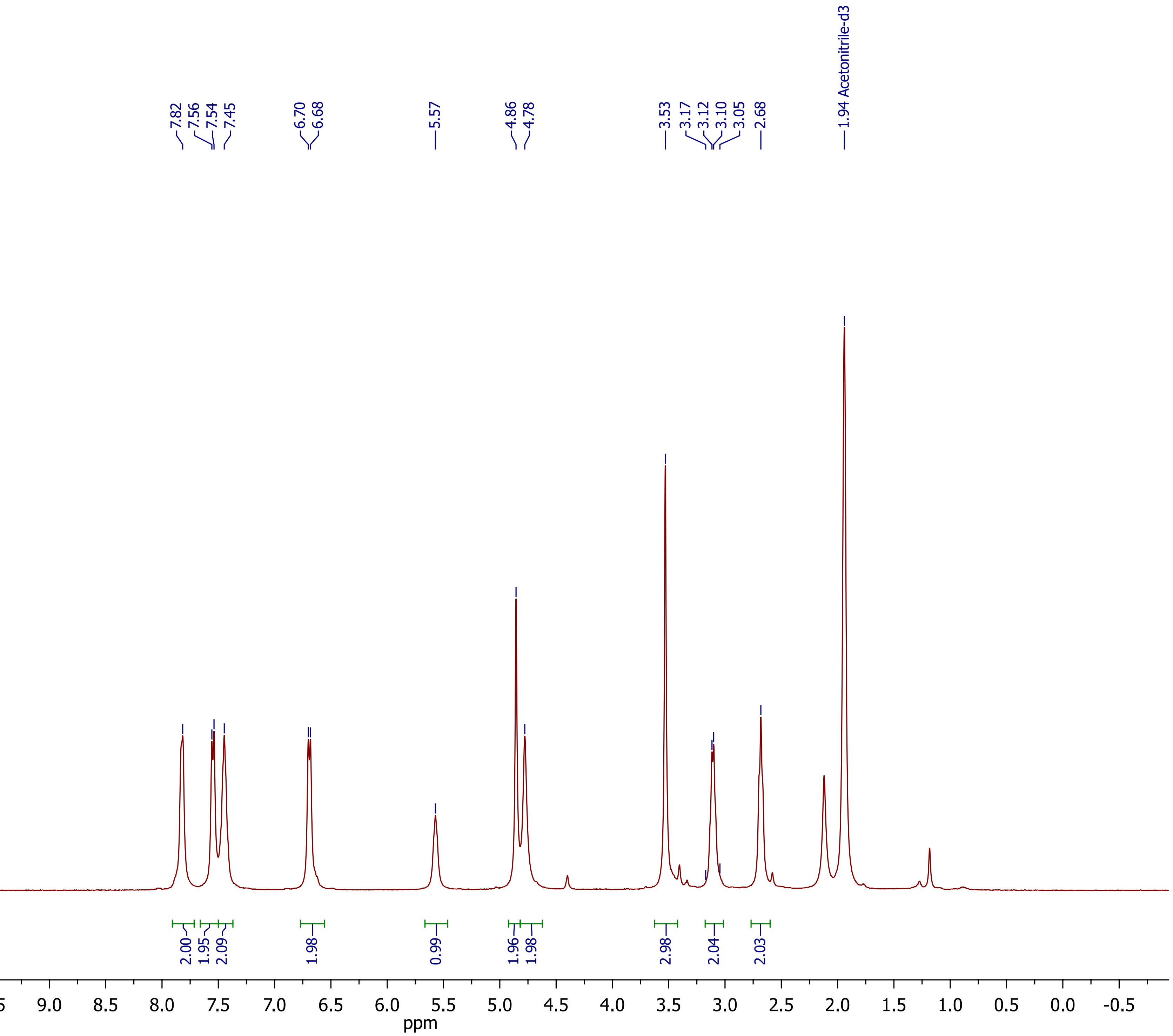

$^{13}C\{^1H\}$ NMR, CD_3CN, 101 MHz

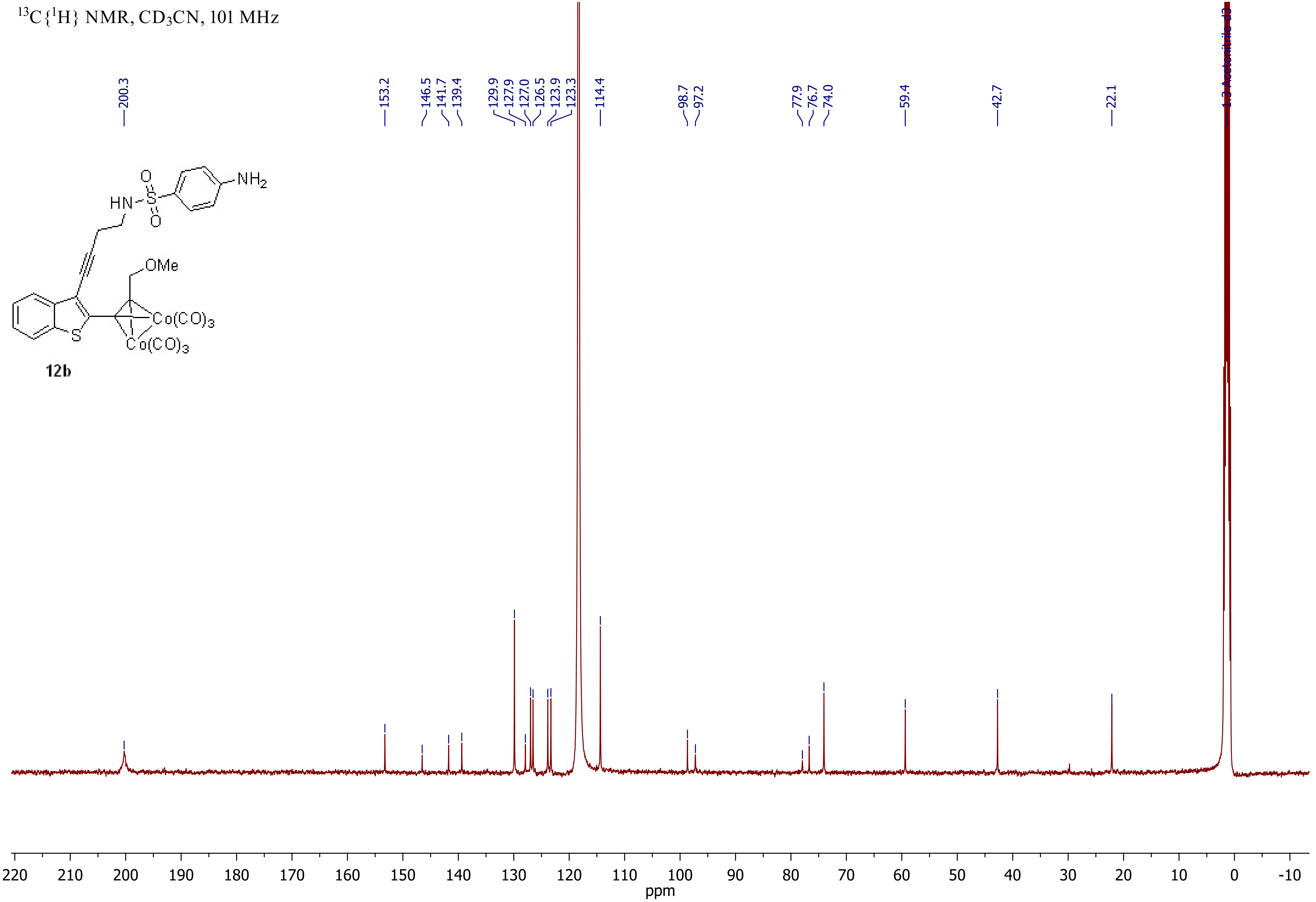

DEPT NMR, CD_3CN, 101 MHz

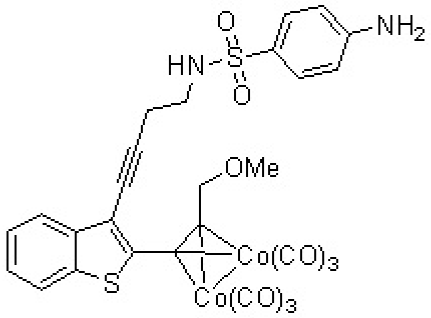

12b

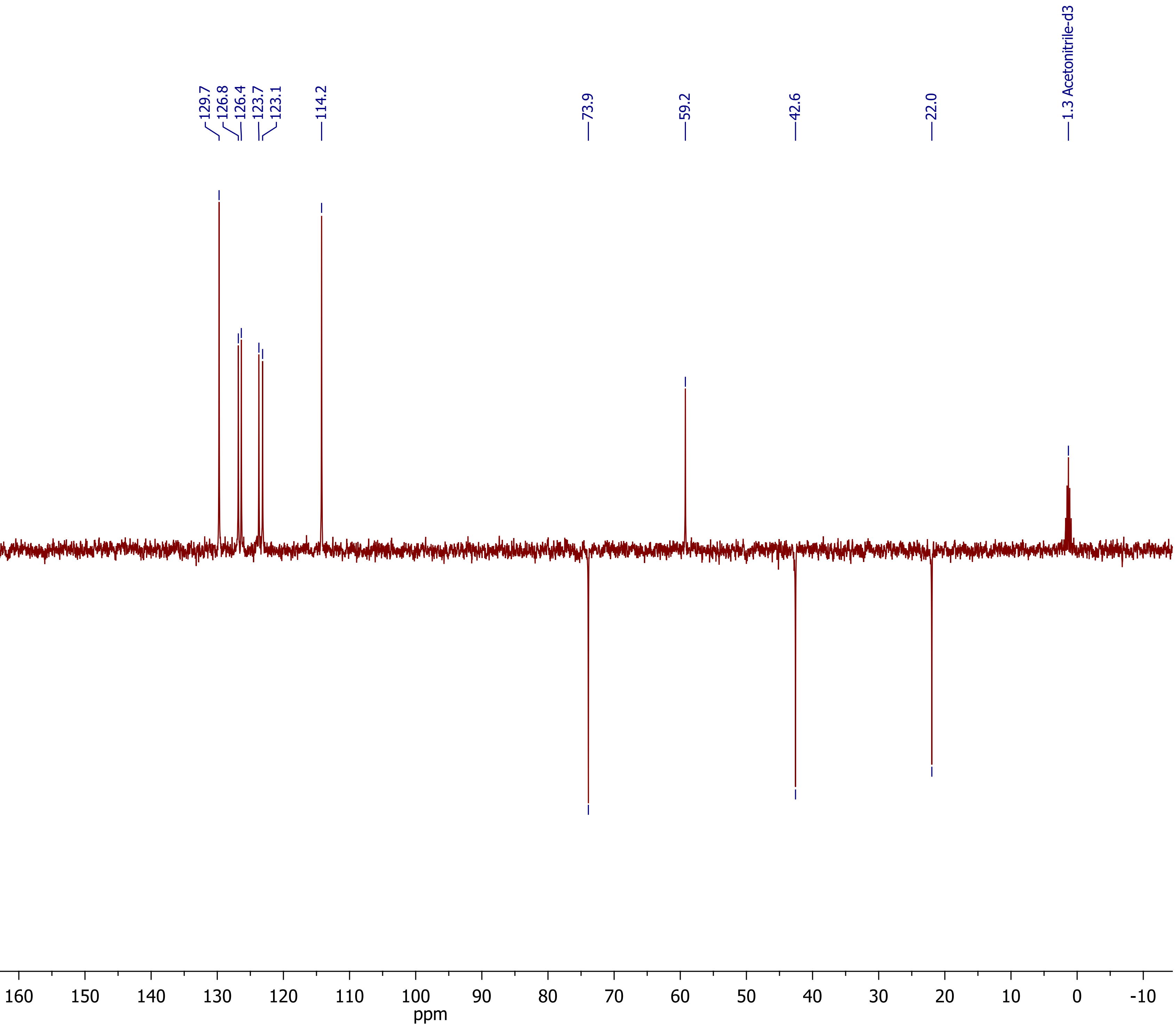

^{1}H NMR, acetone-d_6, 400 MHz

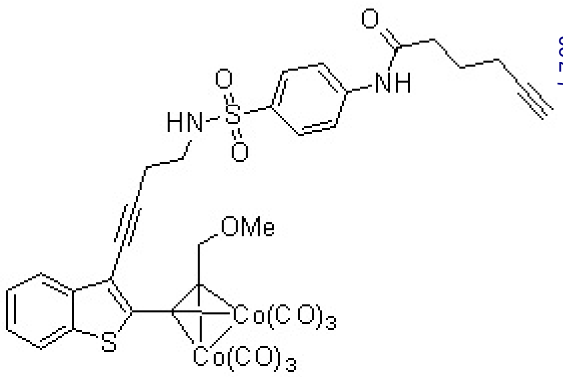

12c

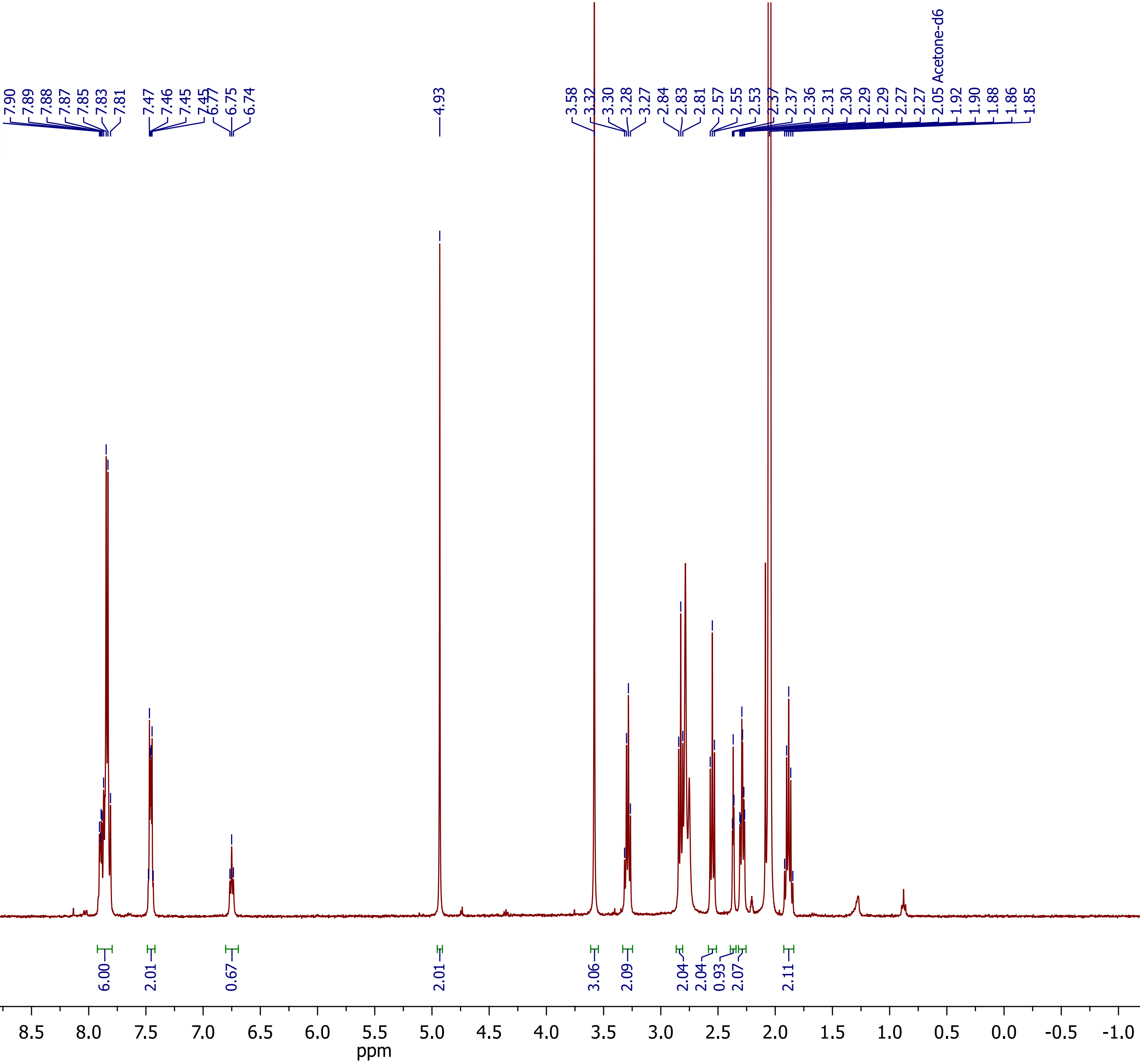

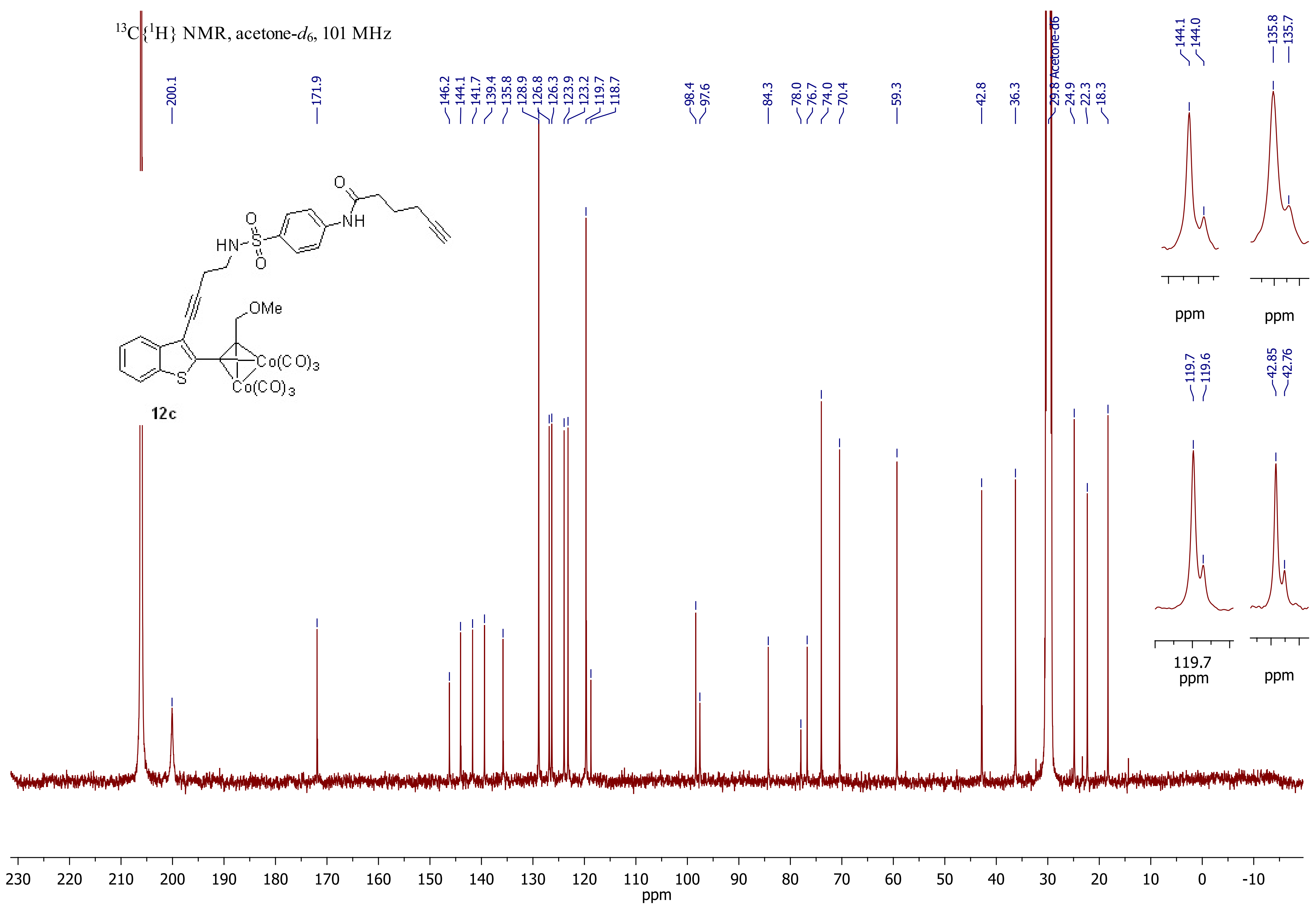

$^{13}C\{^1H\}$ NMR, acetone-d_6, 101 MHz
12c
200.1
171.9
146.2
144.1
141.7
139.4
135.8
128.9
126.8
126.3
123.9
123.2
119.7
118.7
98.4
97.6
84.3
78.0
76.7
74.0
70.4
59.3
42.8
36.3
29.8 Acetone-d6
24.9
22.3
18.3
144.1
144.0
135.8
135.7
119.7
119.6
42.85
42.76
ppm

DEPT NMR, acetone-d_6, 101 MHz

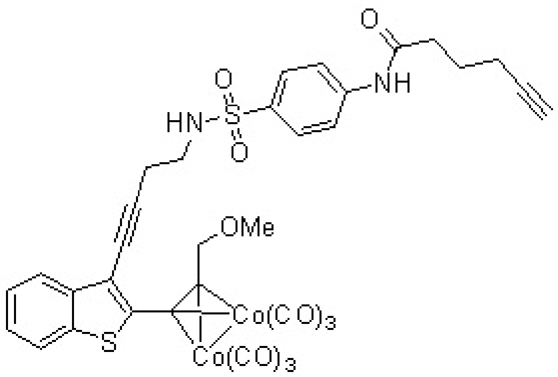

12c

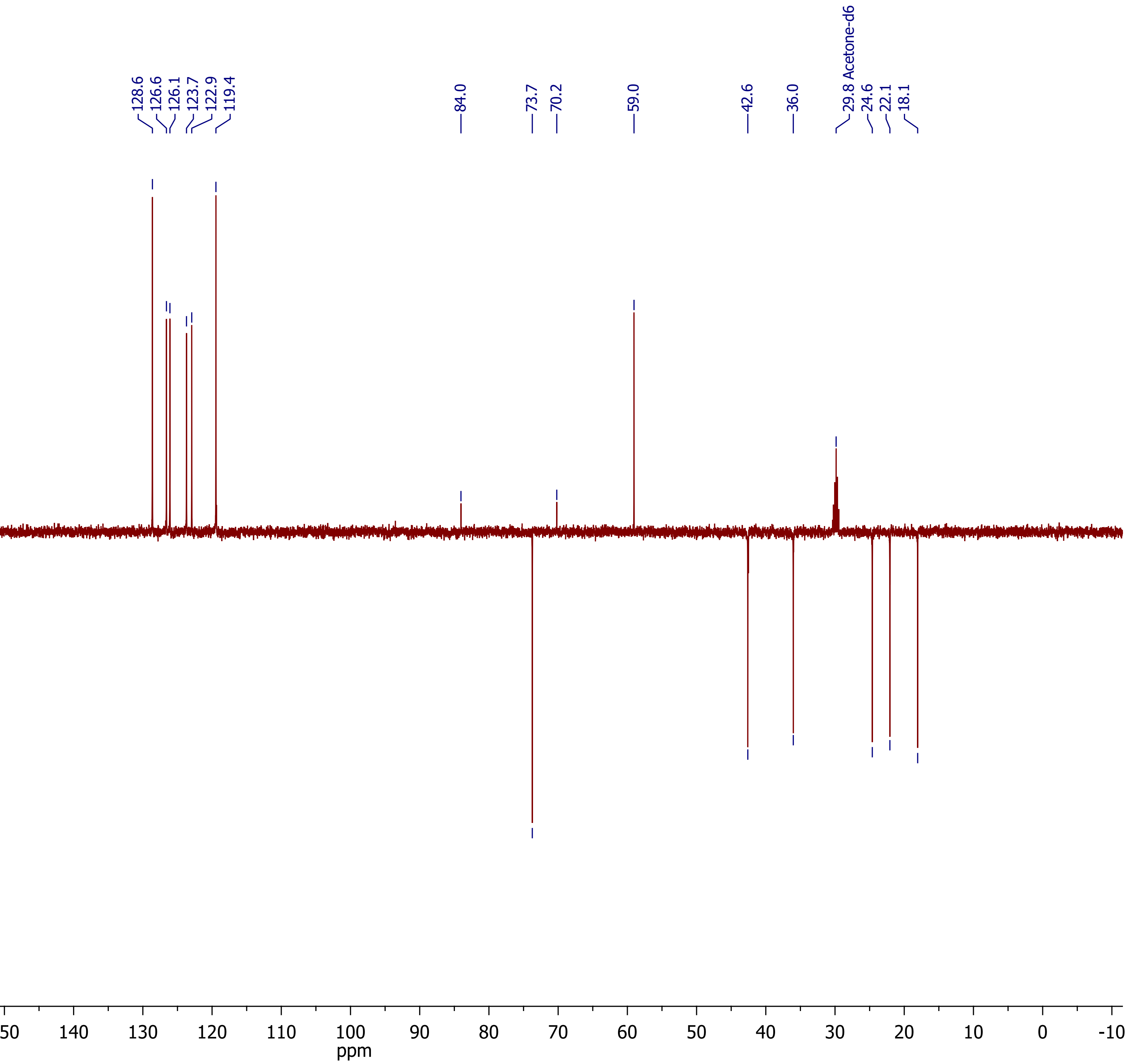

^{1}H NMR, acetone-d_6, 400 MHz

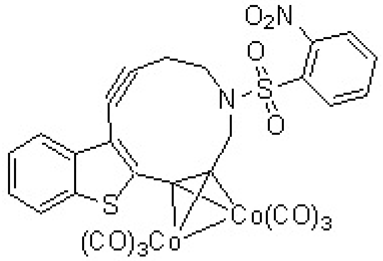

13a

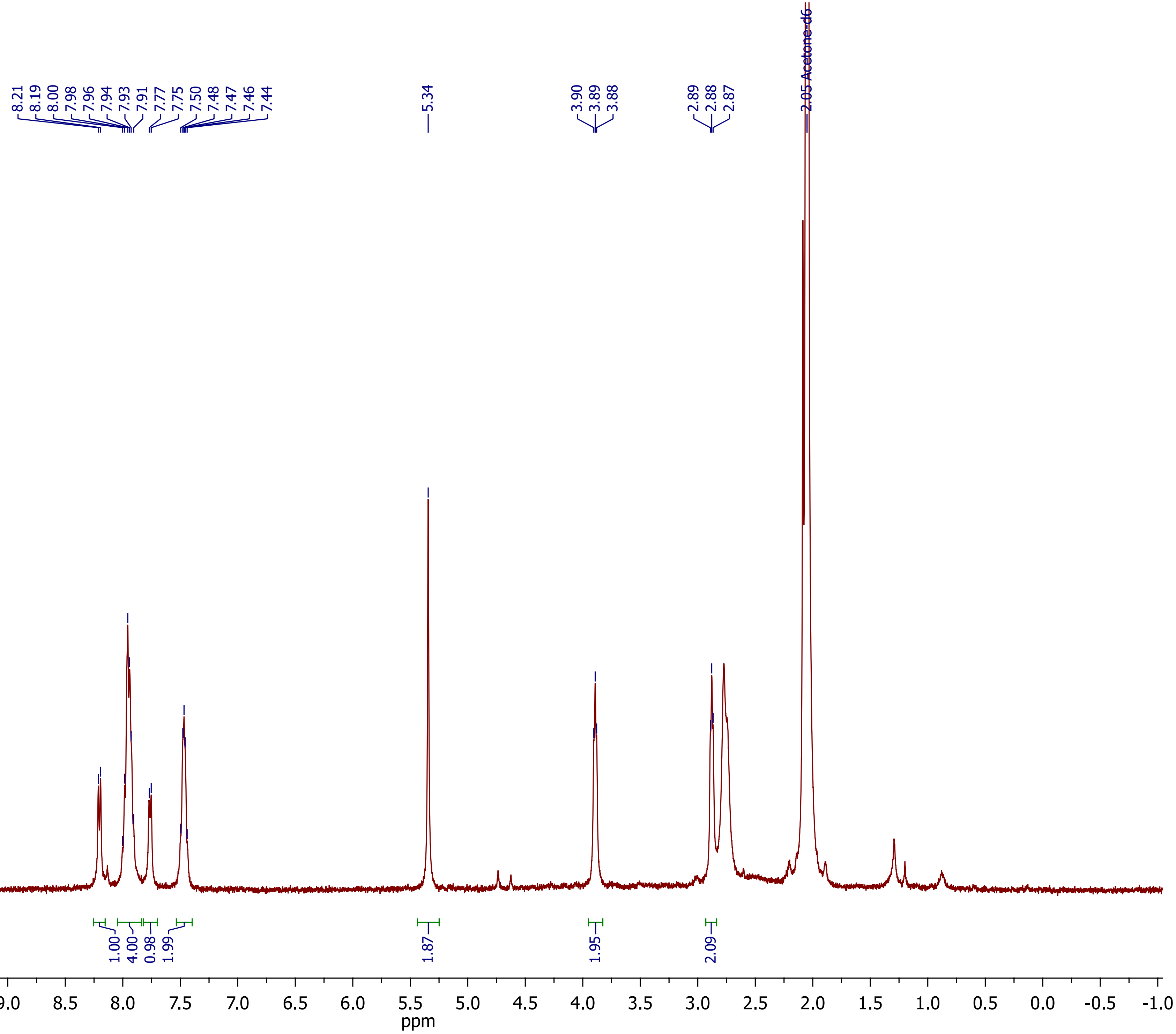

$^{13}C\{^{1}H\}$ NMR, acetone-d_6, 101 MHz

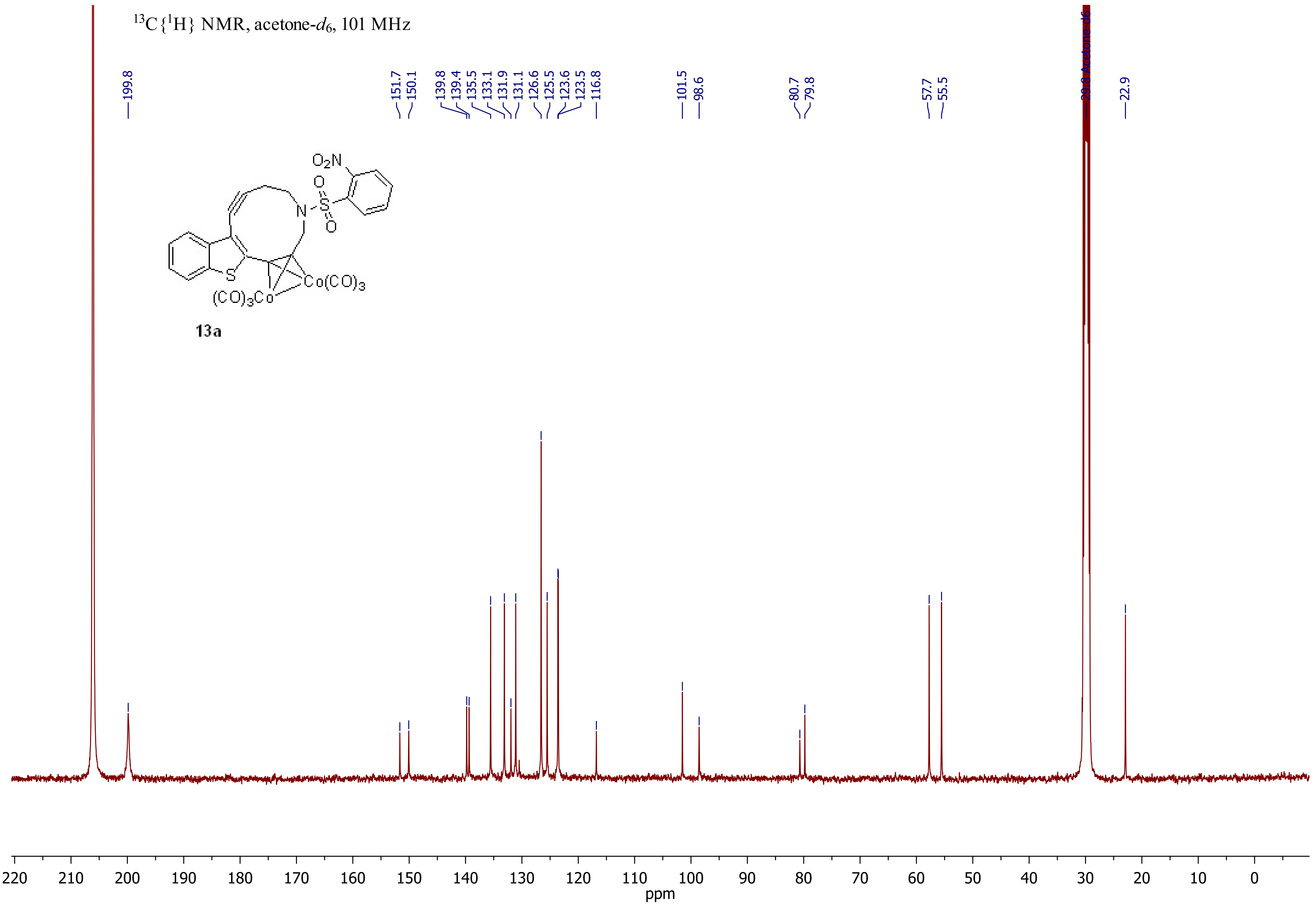

DEPT NMR, acetone-d_6, 101 MHz

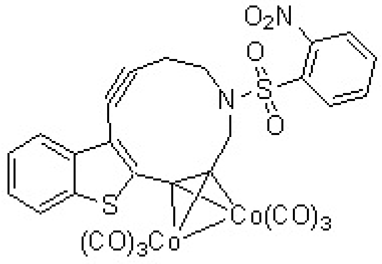

13a

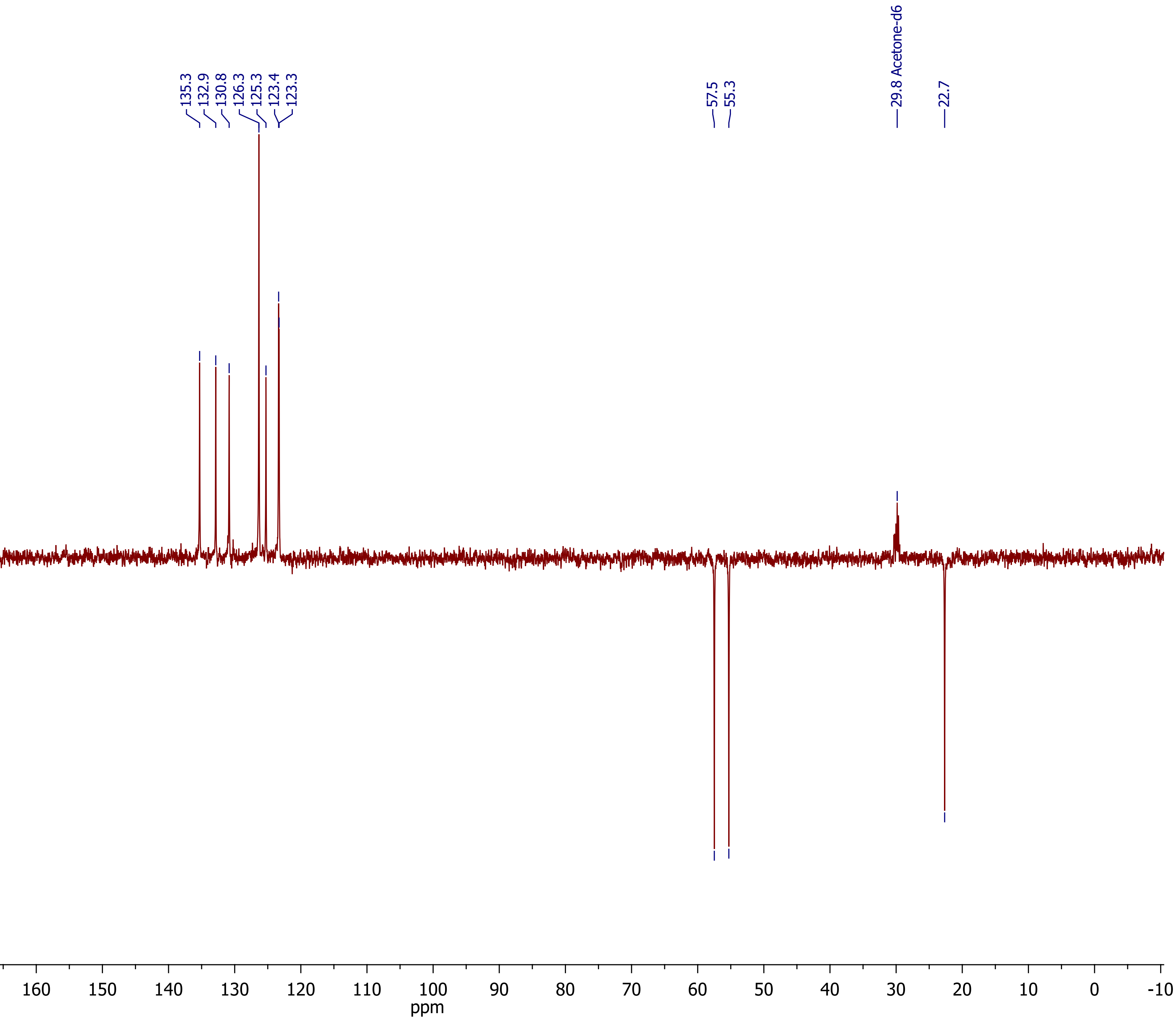

^{1}H NMR, acetone-d_6, 400 MHz

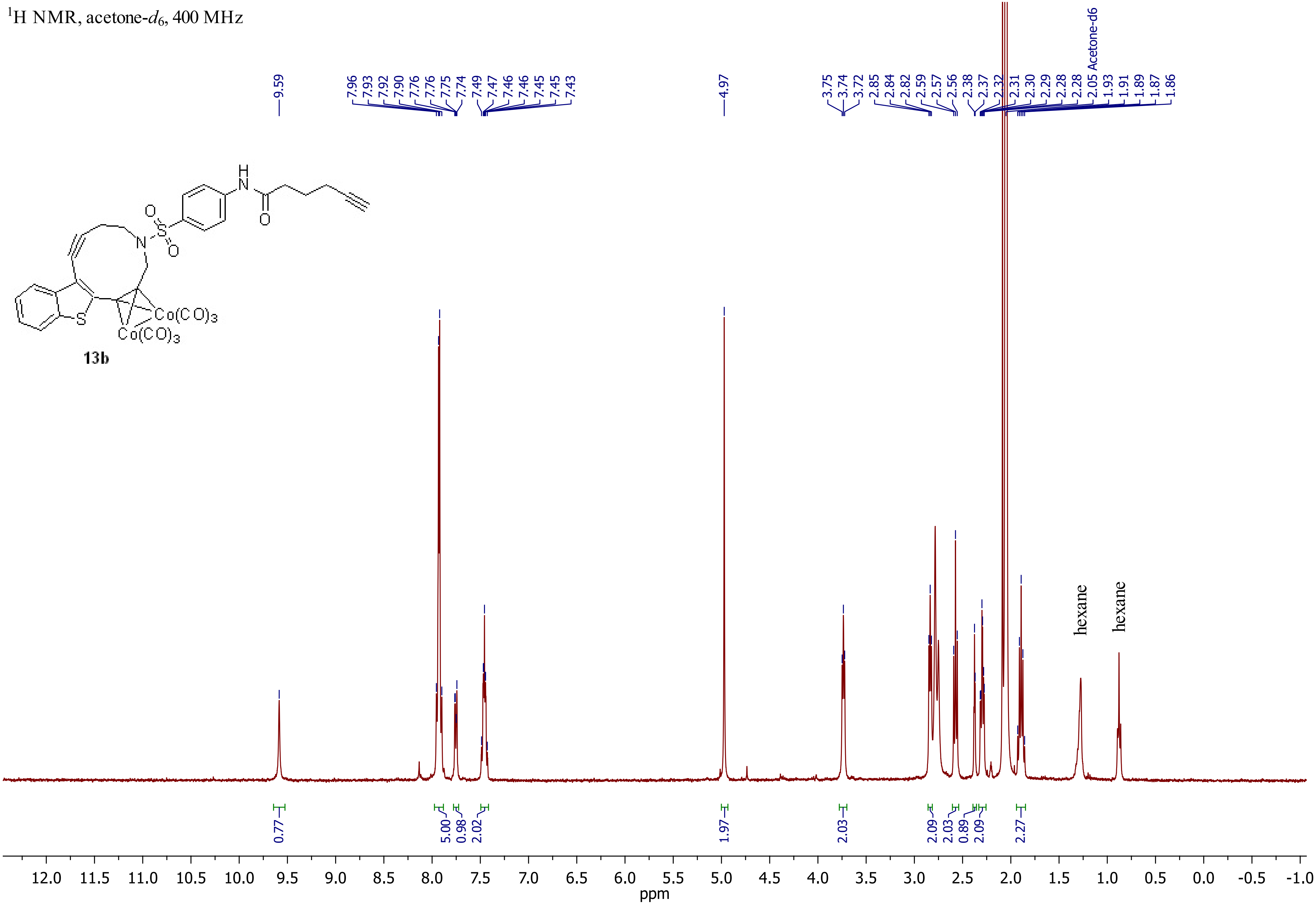

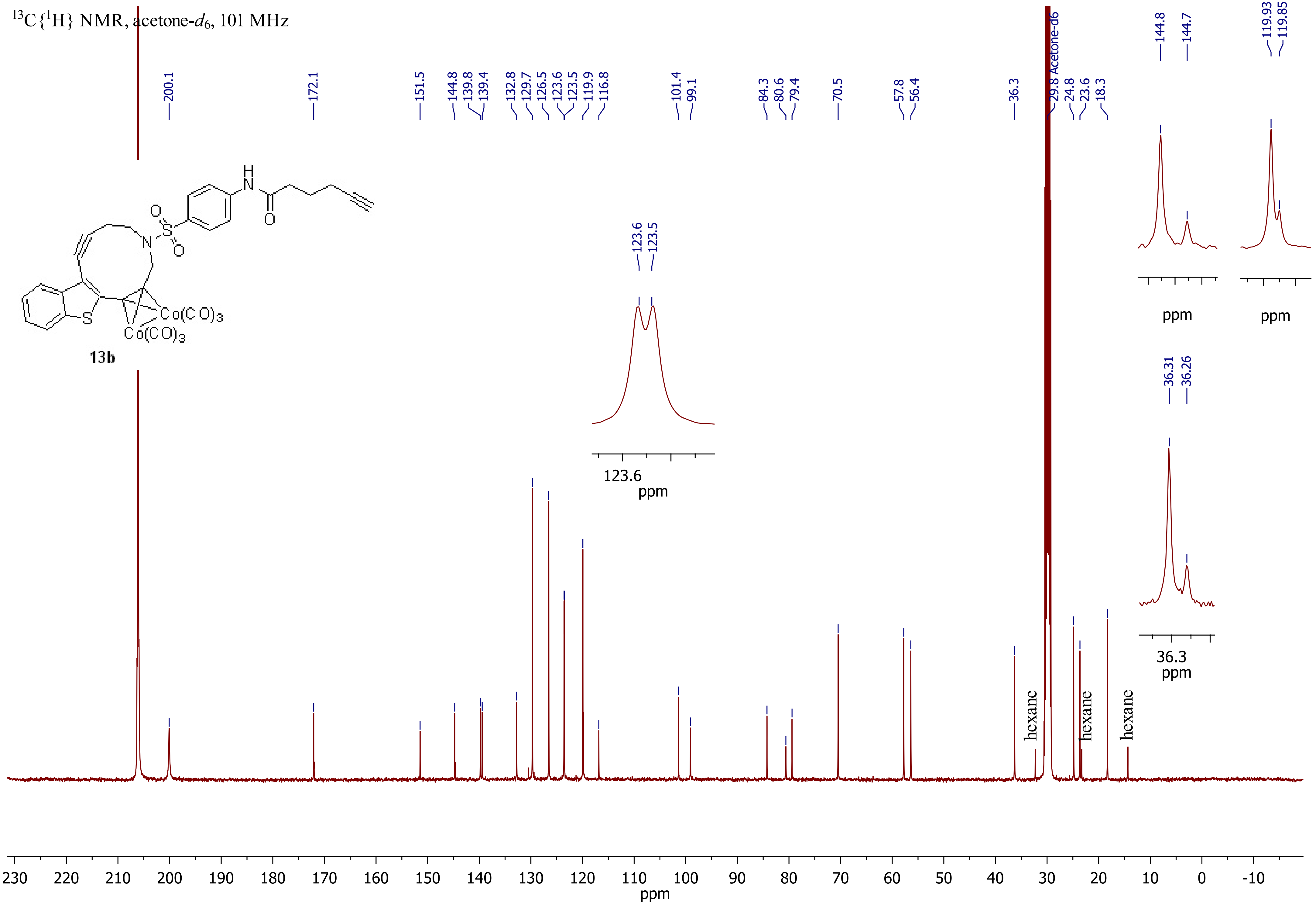
$^{13}C\{^1H\}$ NMR, acetone-d_6, 101 MHz
200.1
172.1
151.5
144.8
139.8
139.4
132.8
129.7
126.5
123.6
123.5
119.9
116.8
101.4
99.1
84.3
80.6
79.4
70.5
57.8
56.4
36.3
29.8 Acetone-d6
24.8
23.6
18.3
144.8
144.7
119.93
119.85
36.31
36.26
13b
hexane
hexane
hexane
ppm

DEPT NMR, acetone-d_6, 101 MHz

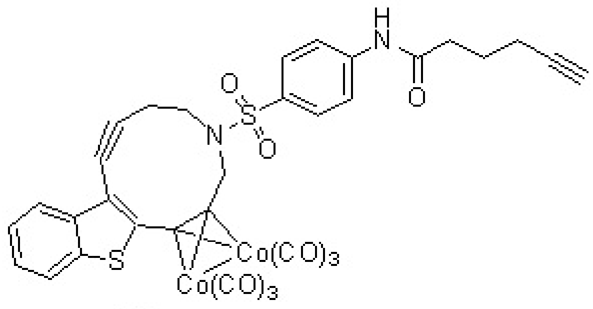

13b

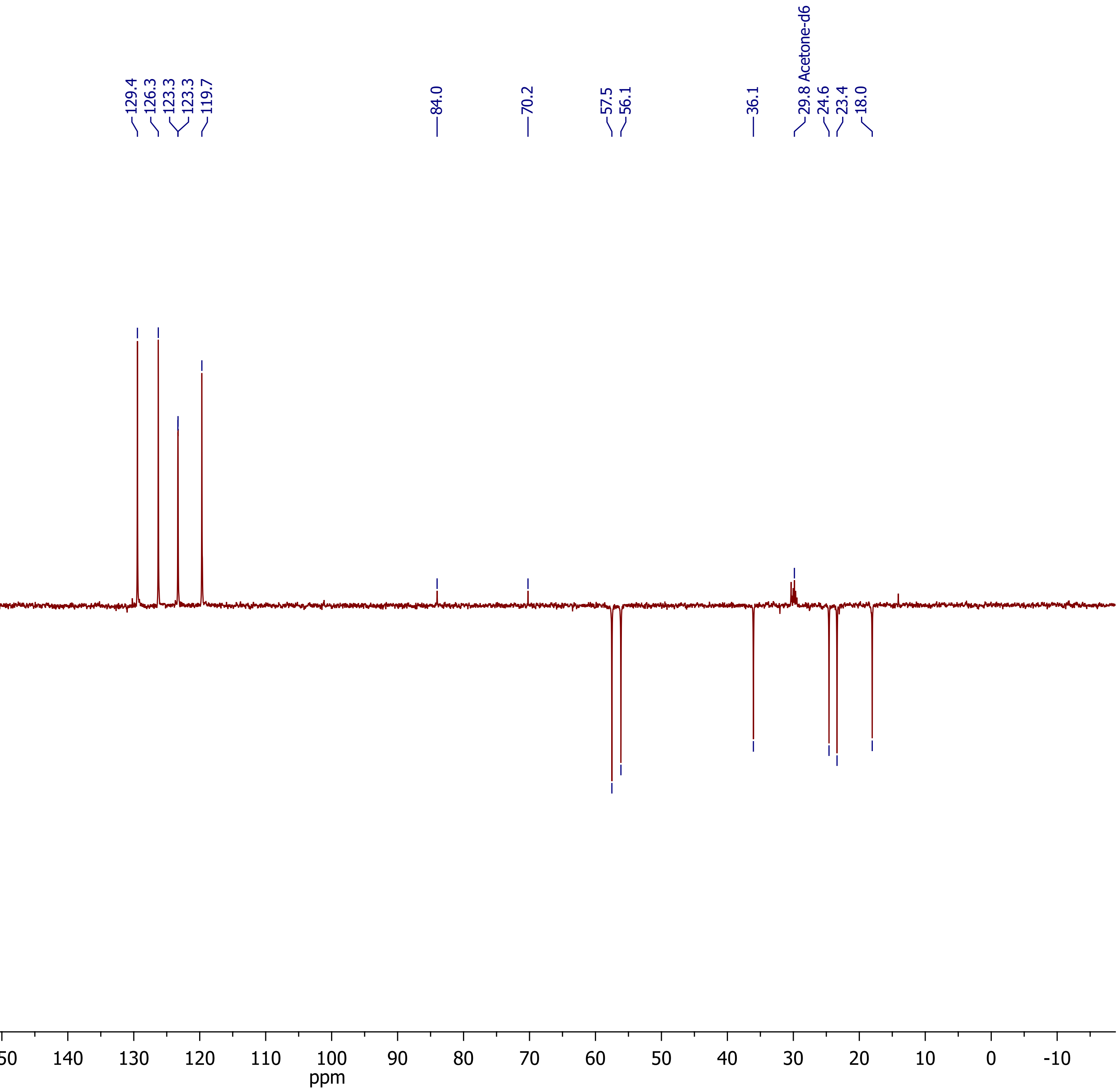

^{1}H NMR, acetone-d_6, 400 MHz

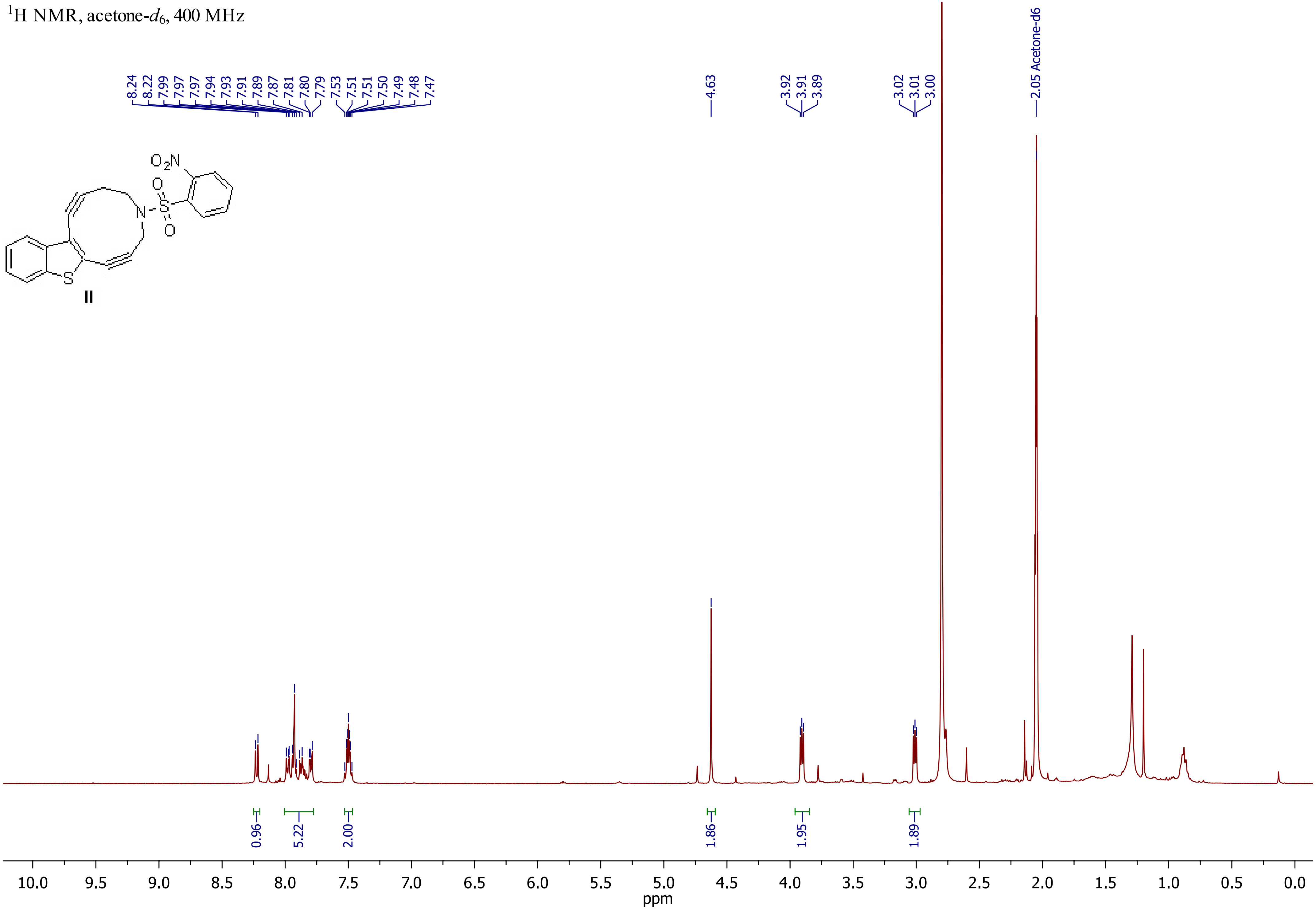

$^{13}C\{^1H\}$ NMR, acetone-d_6, 101 MHz

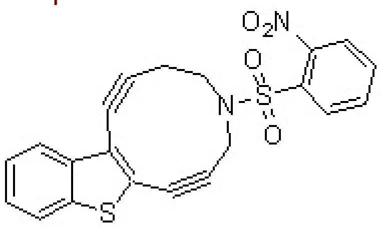

II

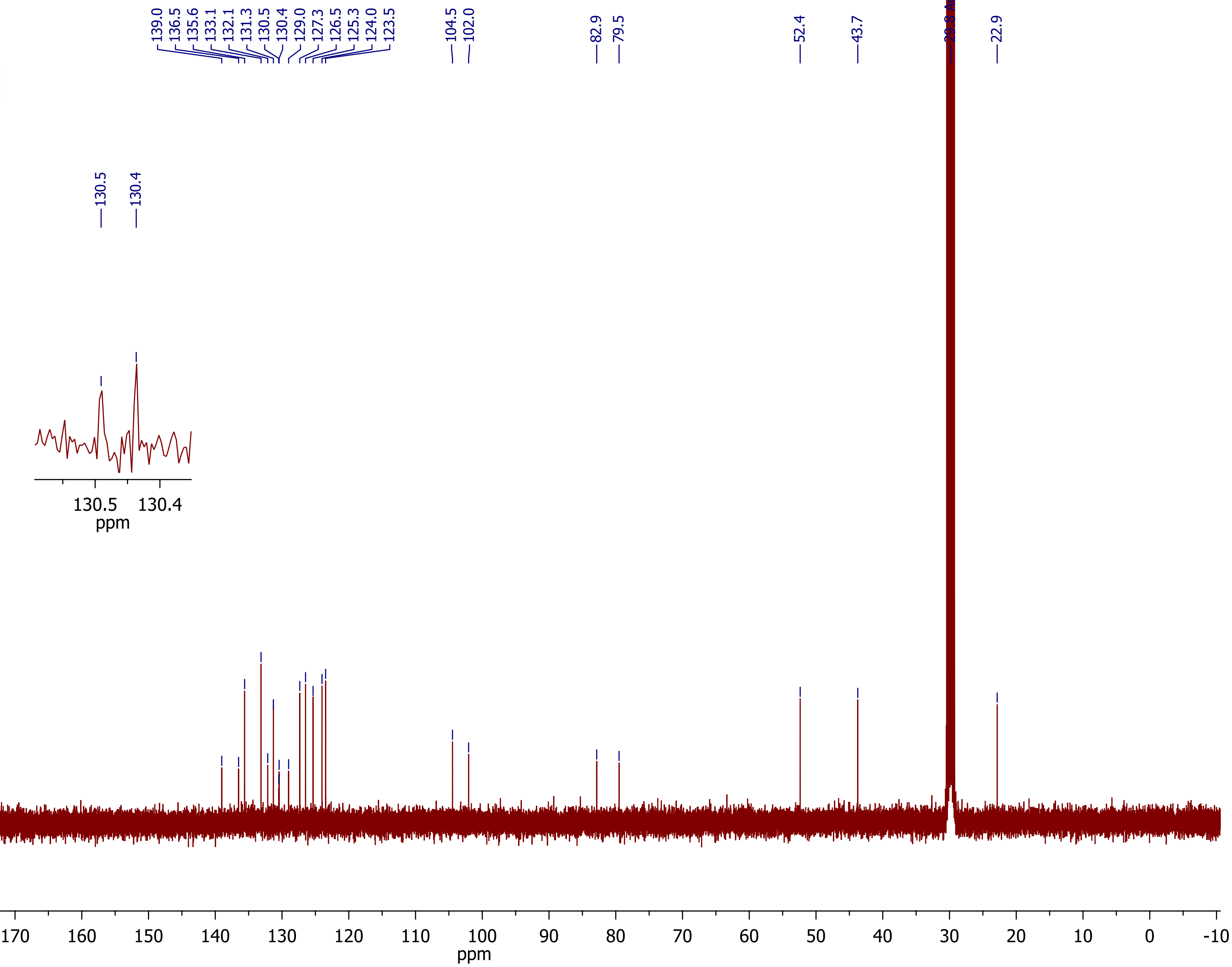

DEPT NMR, acetone-d_6, 101 MHz

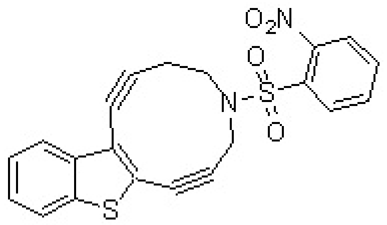

II

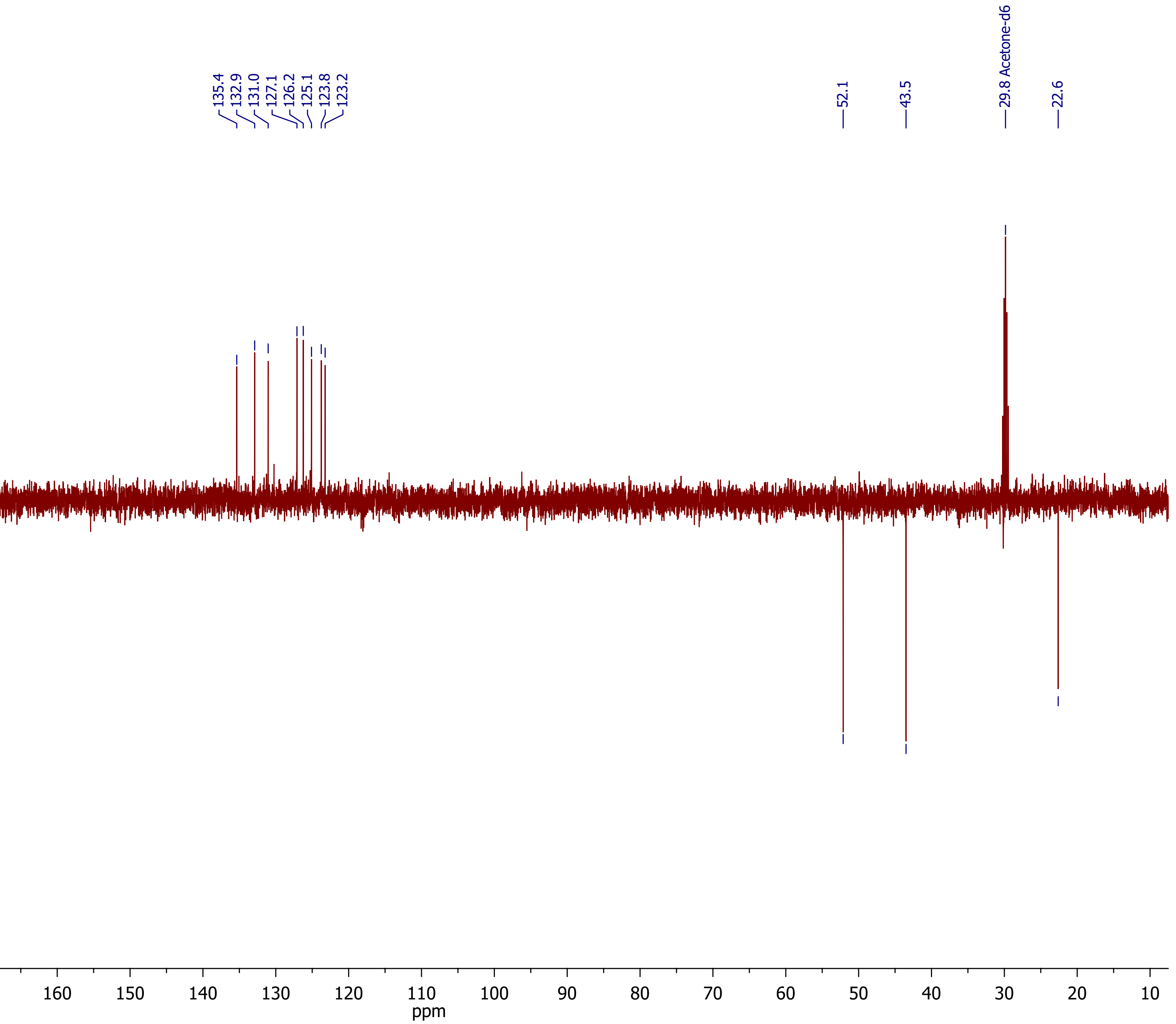

^{1}H NMR, acetone-d_6, 400 MHz

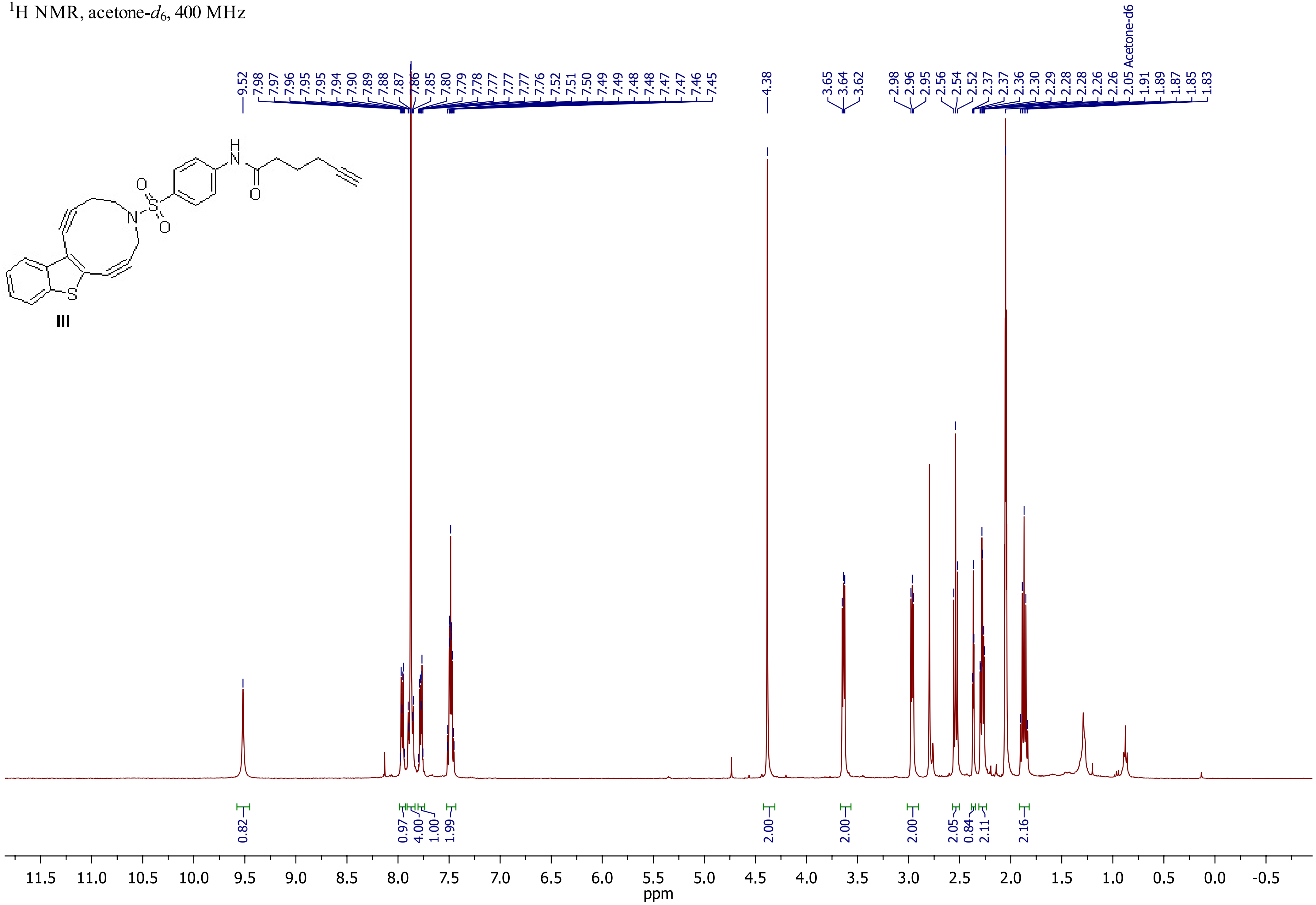

$^{13}C\{^1H\}$ NMR, acetone-d_6, 101 MHz

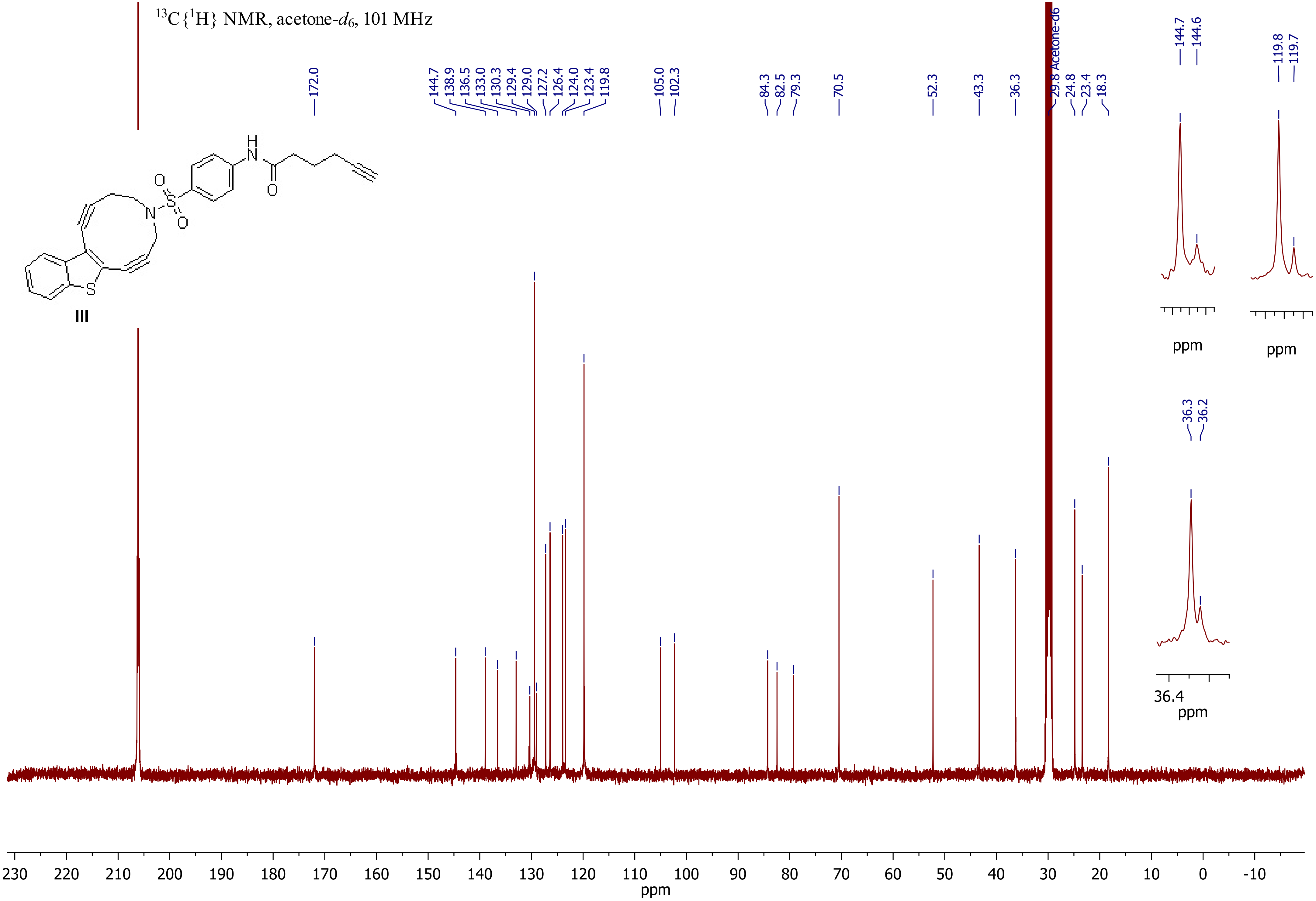

DEPT NMR, acetone-d_6, 101 MHz

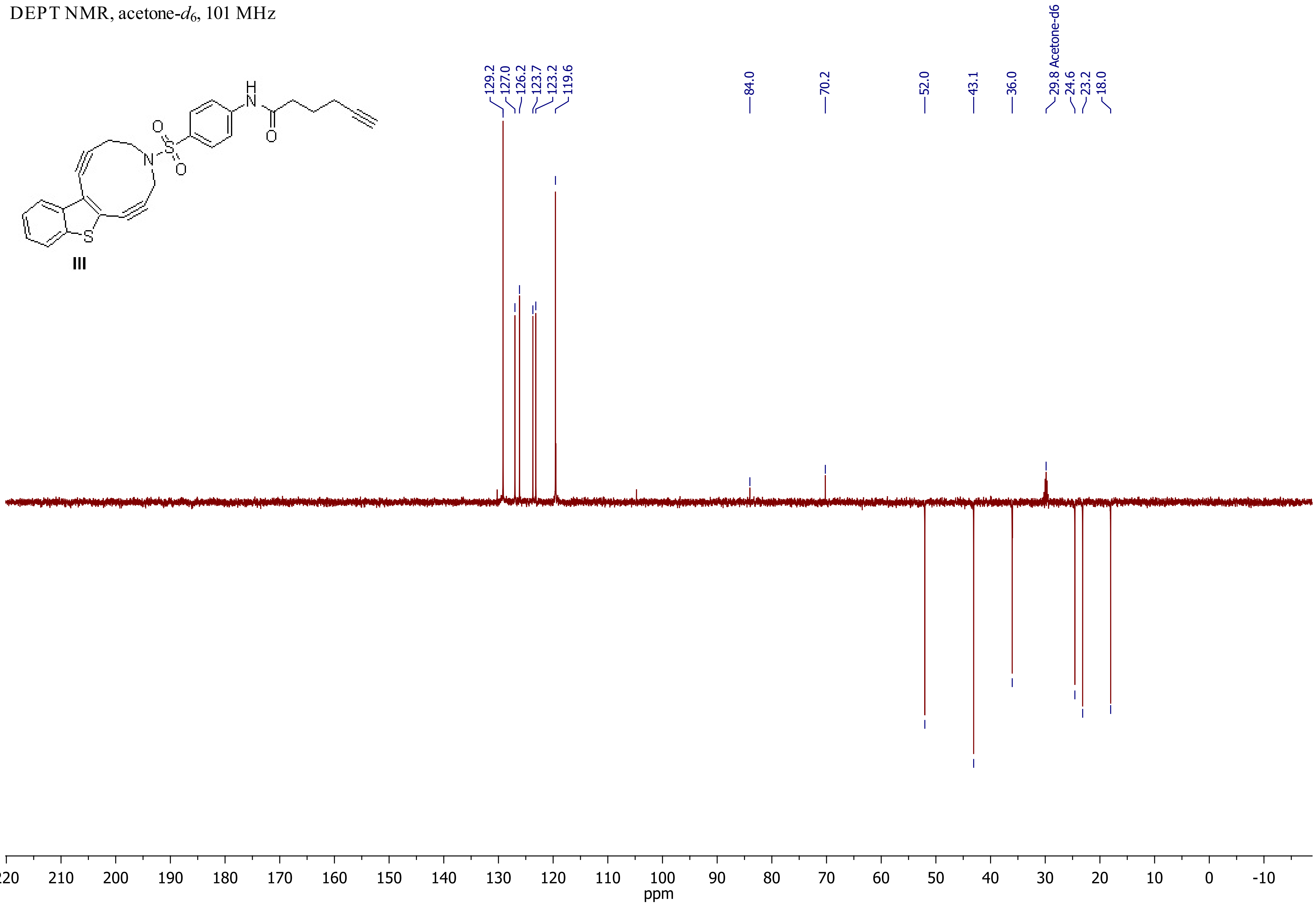

^{1}H NMR, acetone-d_6, 400 MHz

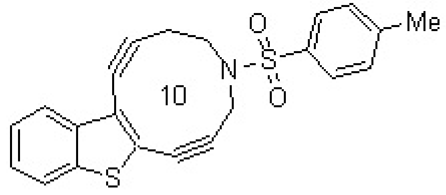

14

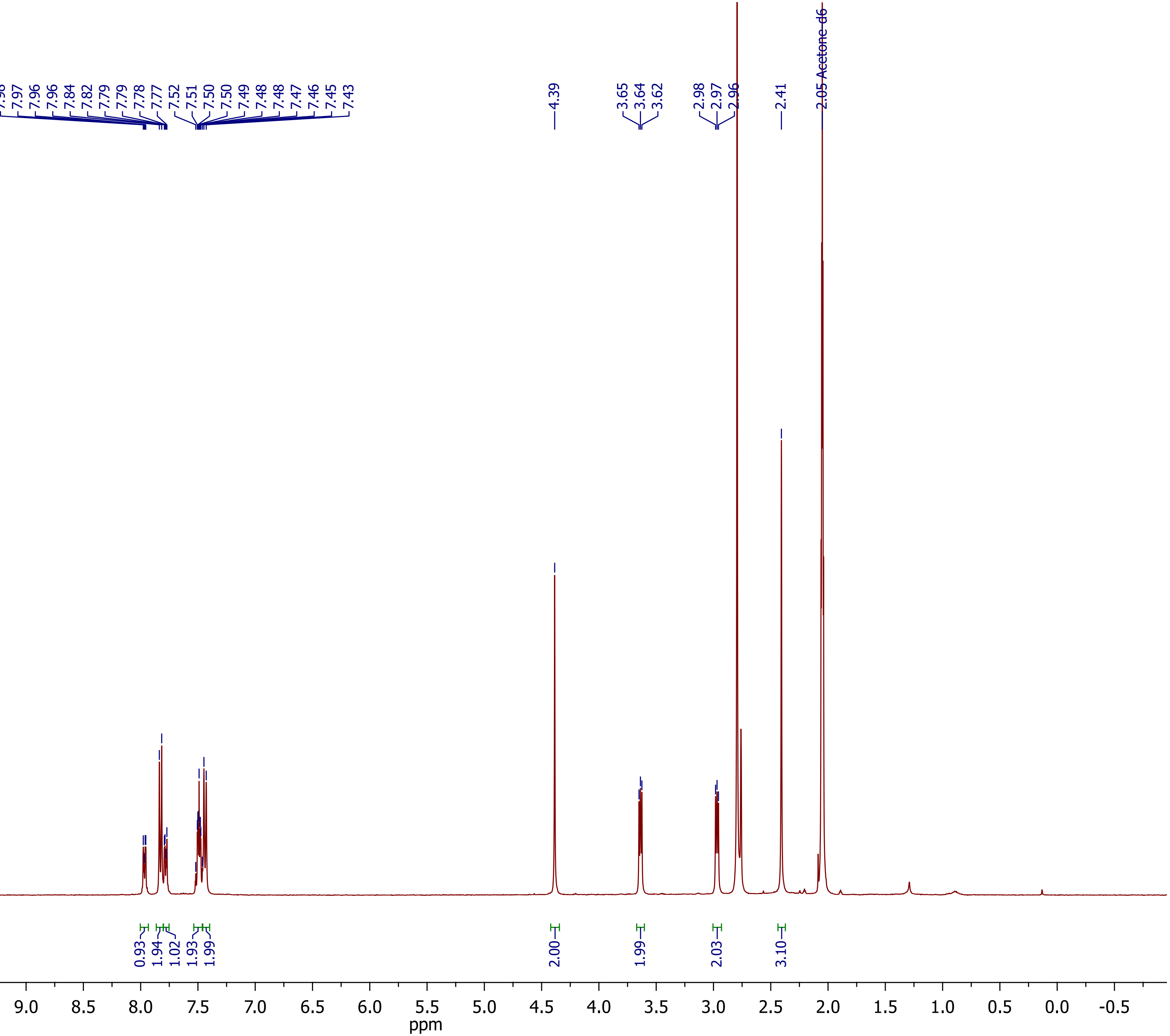

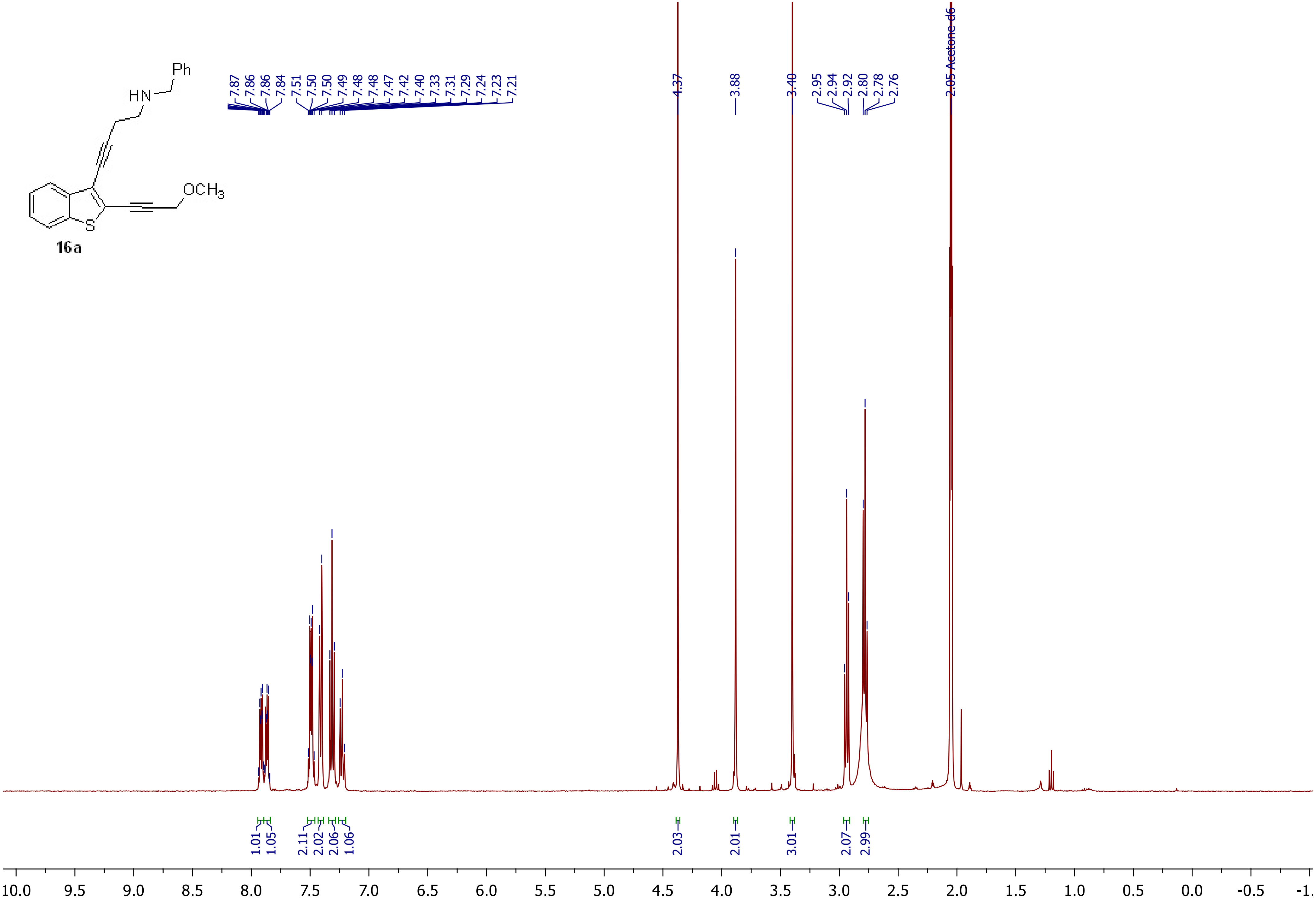
Ph
HN
OCH3
S
16a
7.87 7.86 7.86 7.84 7.51 7.50 7.50 7.49 7.48 7.48 7.47 7.42 7.40 7.33 7.31 7.29 7.24 7.23 7.21
4.37
3.88
3.40
2.95 2.94 2.92 2.80 2.78 2.76
2.05 Acetone-d6
1.01 1.05 2.11 2.02 2.06 1.06
2.03
2.01
3.01
2.07 2.99
10.0 9.5 9.0 8.5 8.0 7.5 7.0 6.5 6.0 5.5 5.0 4.5 4.0 3.5 3.0 2.5 2.0 1.5 1.0 0.5 0.0 -0.5 -1.
ppm

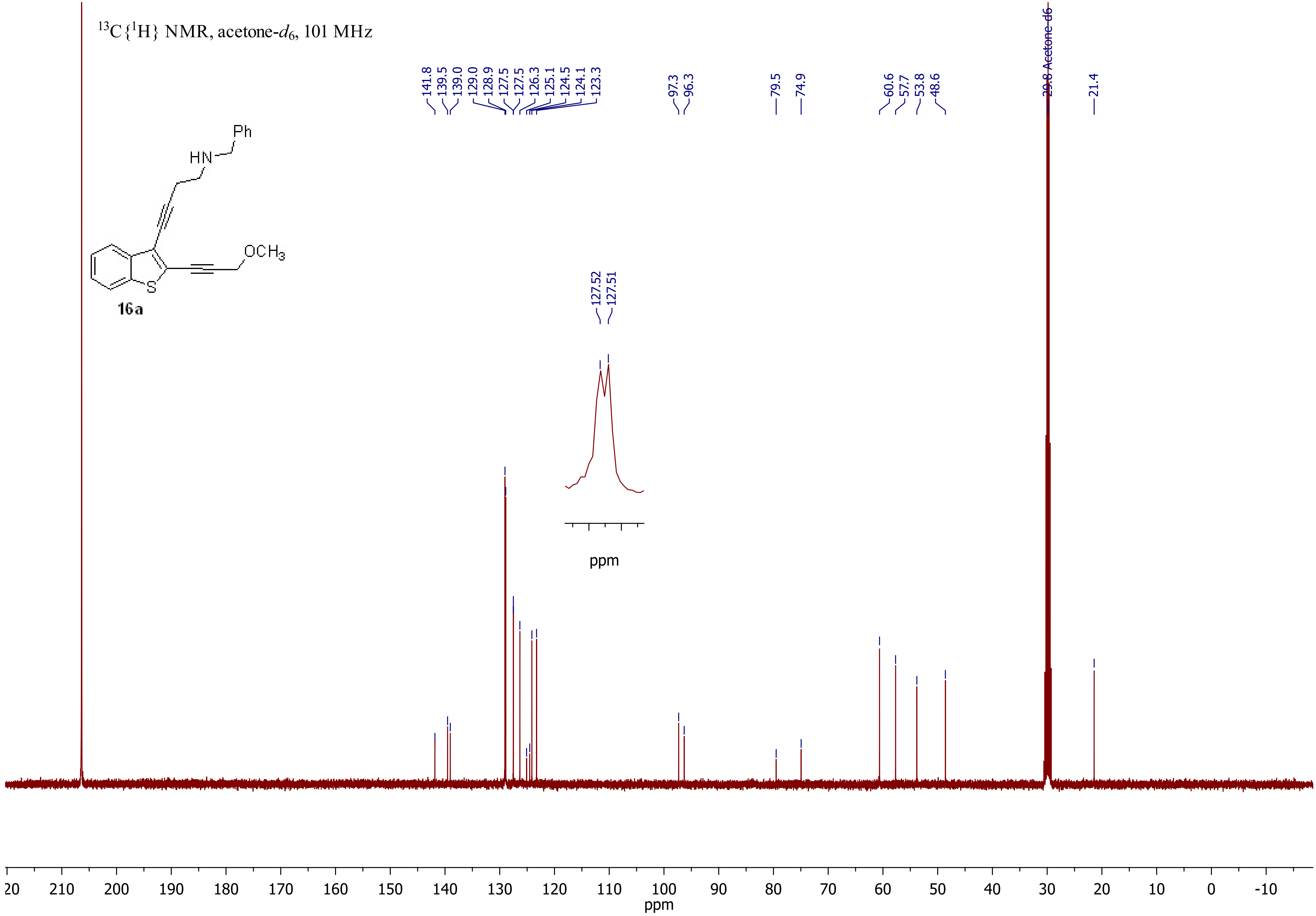

$^{13}C\{^1H\}$ NMR, acetone-d_6, 101 MHz
141.8
139.5
139.0
129.0
128.9
127.5
127.5
126.3
125.1
124.5
124.1
123.3
97.3
96.3
79.5
74.9
60.6
57.7
53.8
48.6
29.8 Acetone-d6
21.4
Ph
HN
OCH3
S
16a
127.52
127.51
ppm
20 210 200 190 180 170 160 150 140 130 120 110 100 90 80 70 60 50 40 30 20 10 0 -10
ppm

DEPT NMR, acetone-d_6, 101 MHz

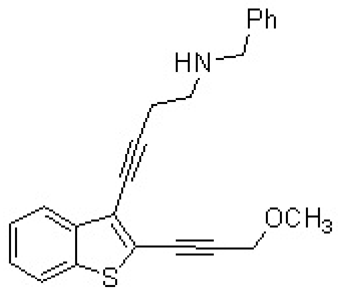

16a

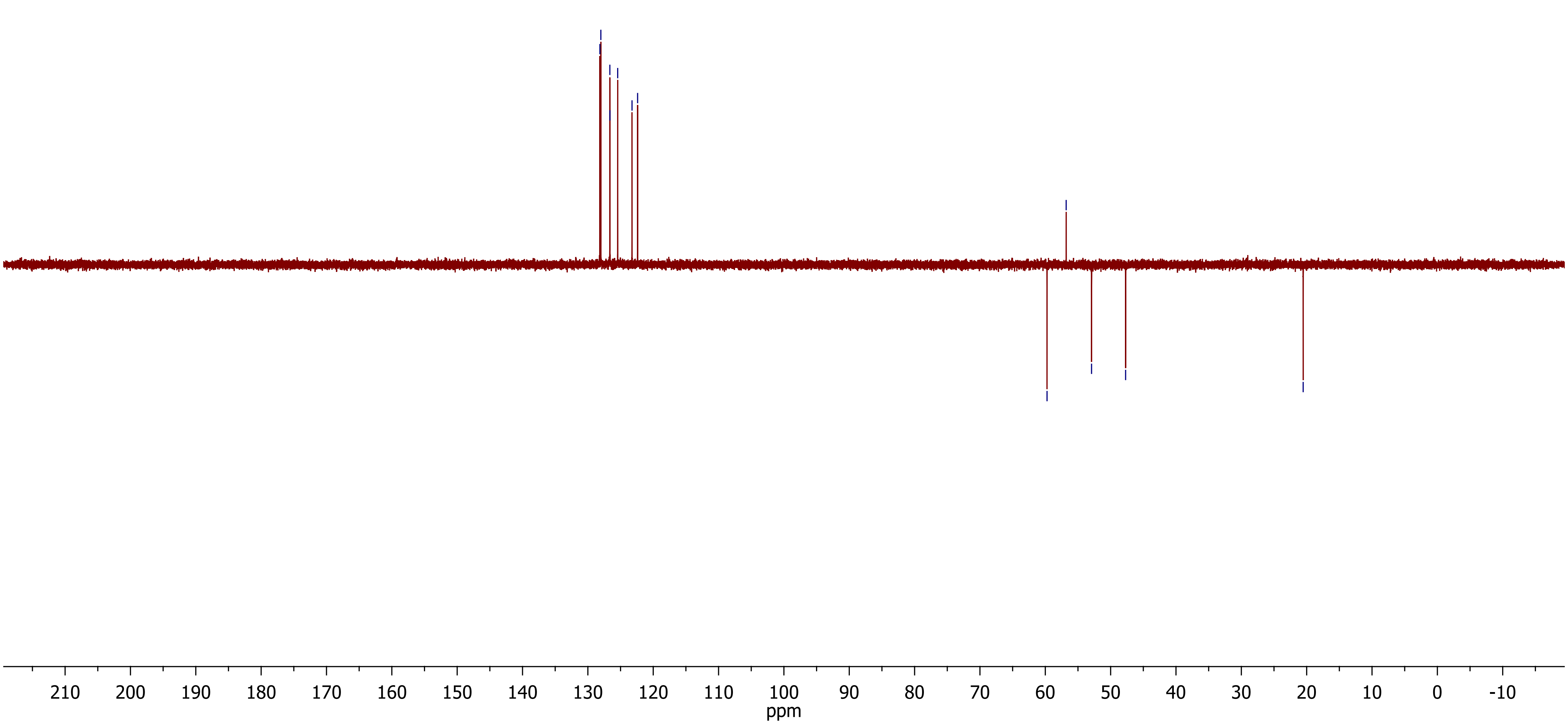

^{1}H NMR, $CDCl_3$, 400 MHz

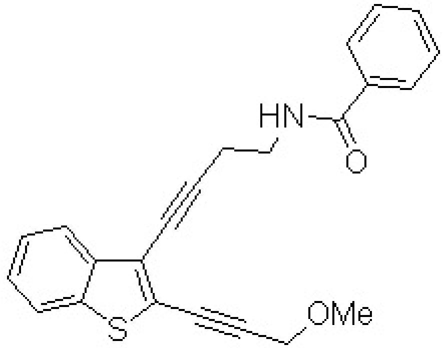

16b

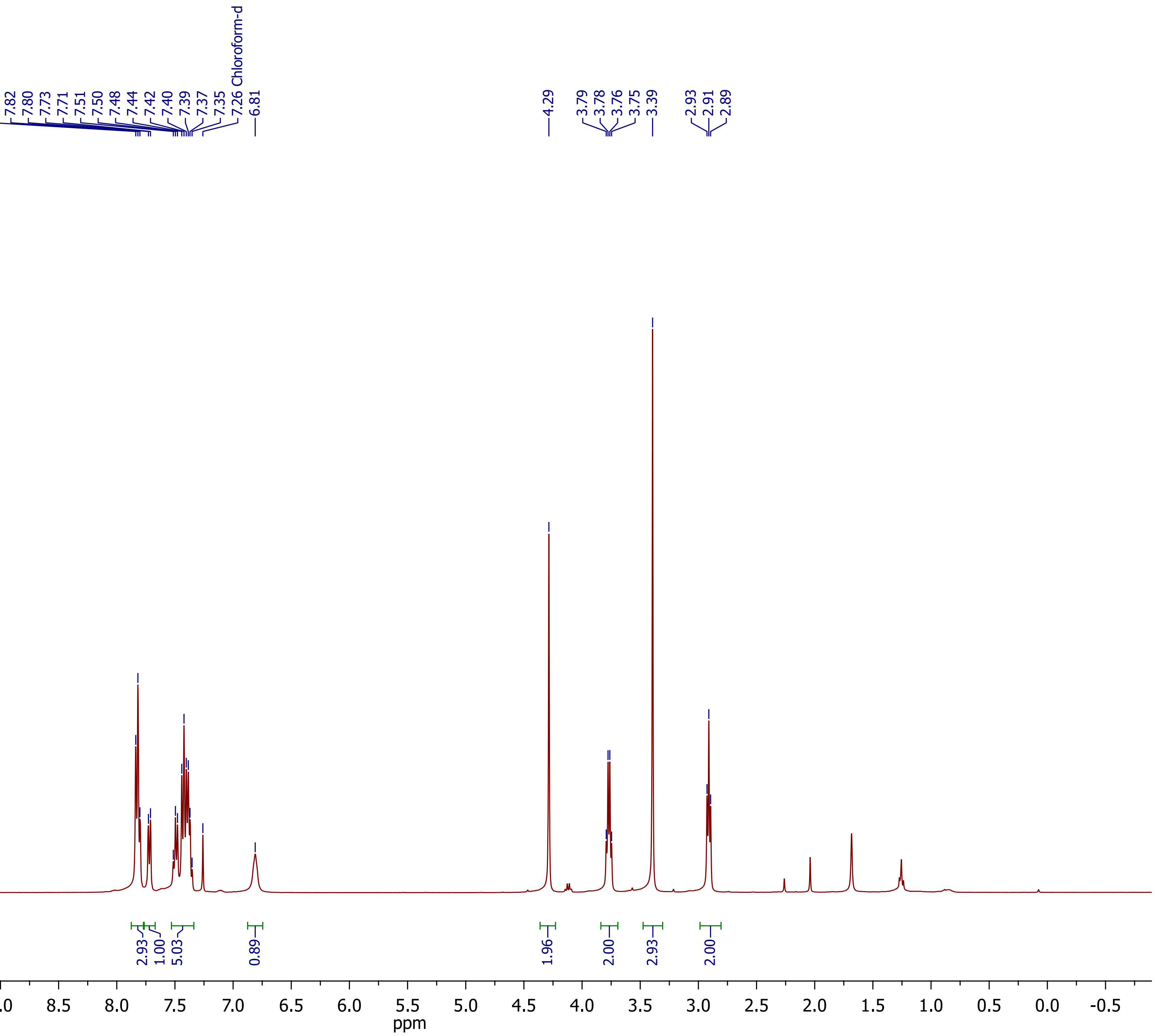

$^{13}C\{^1H\}$ NMR, $CDCl_3$, 101 MHz

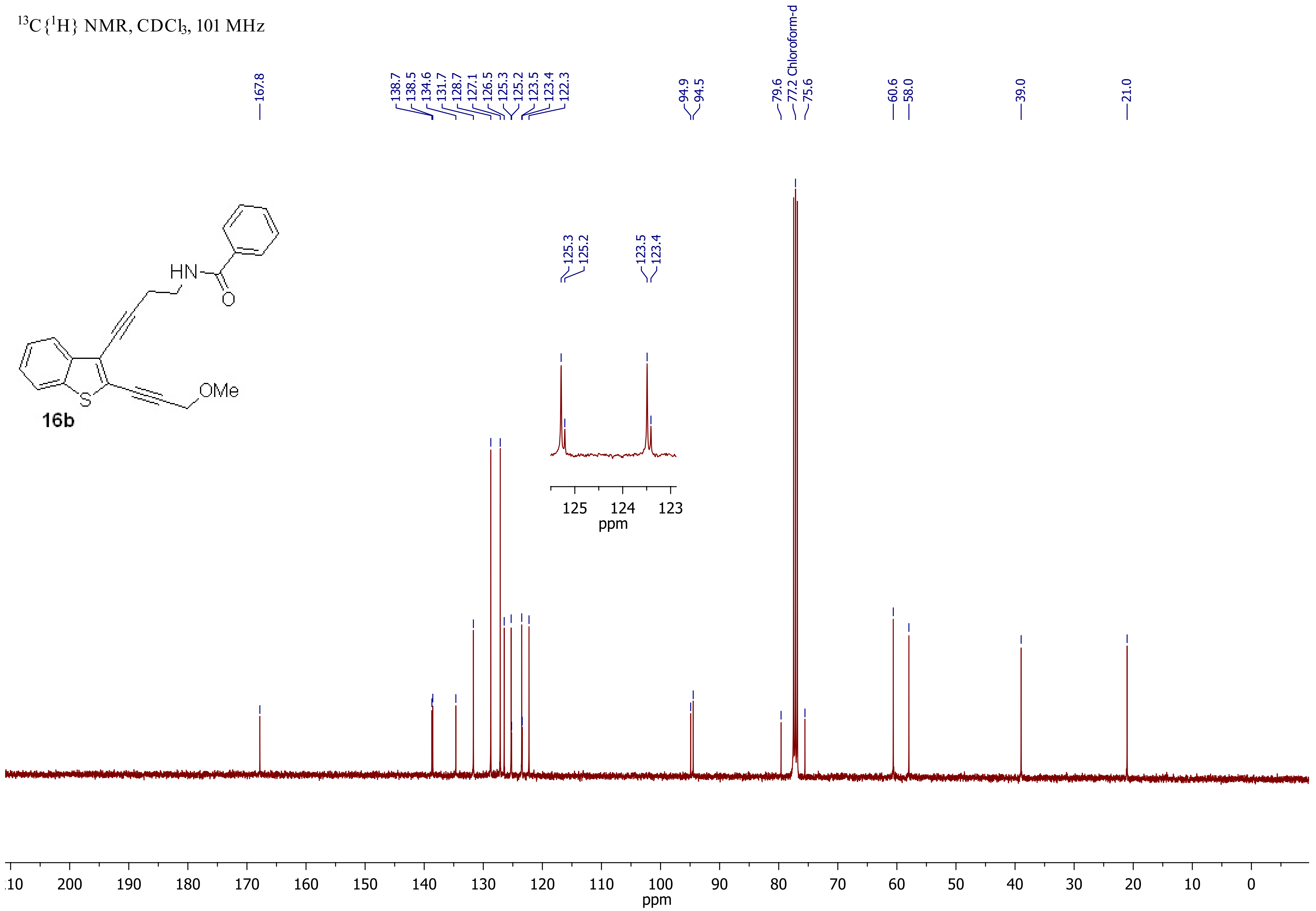

DEPT NMR, $CDCl_3$, 101 MHz

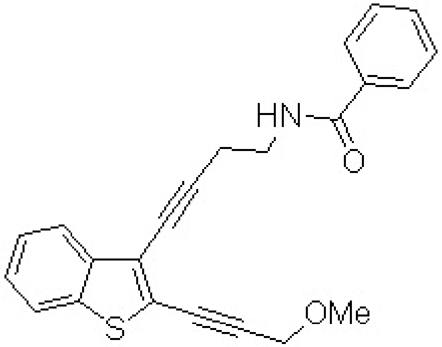

16b

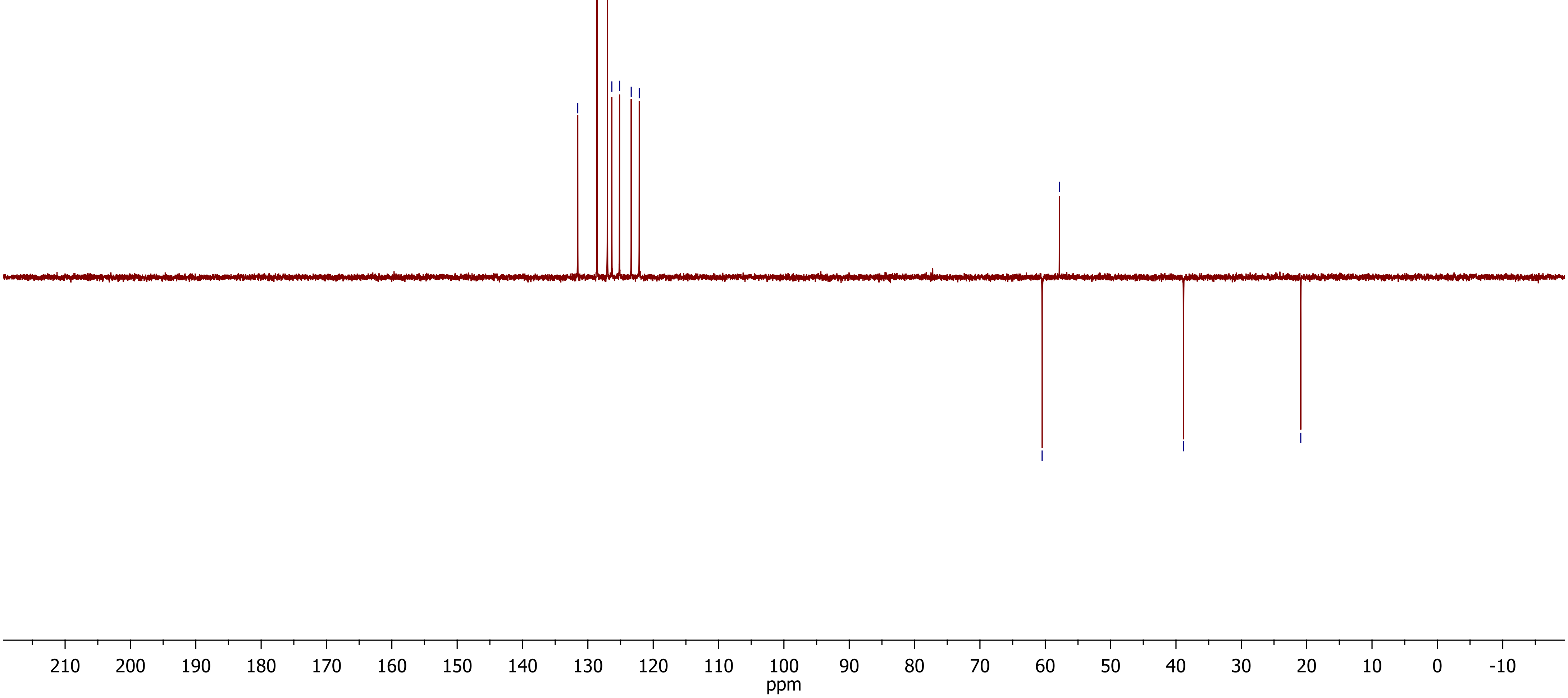

^{1}H NMR, acetone-d_6, 400 MHz

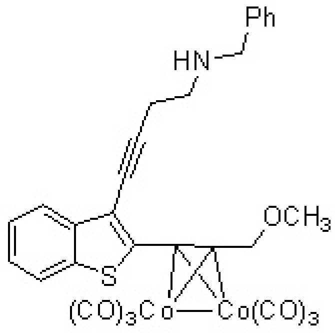

17a

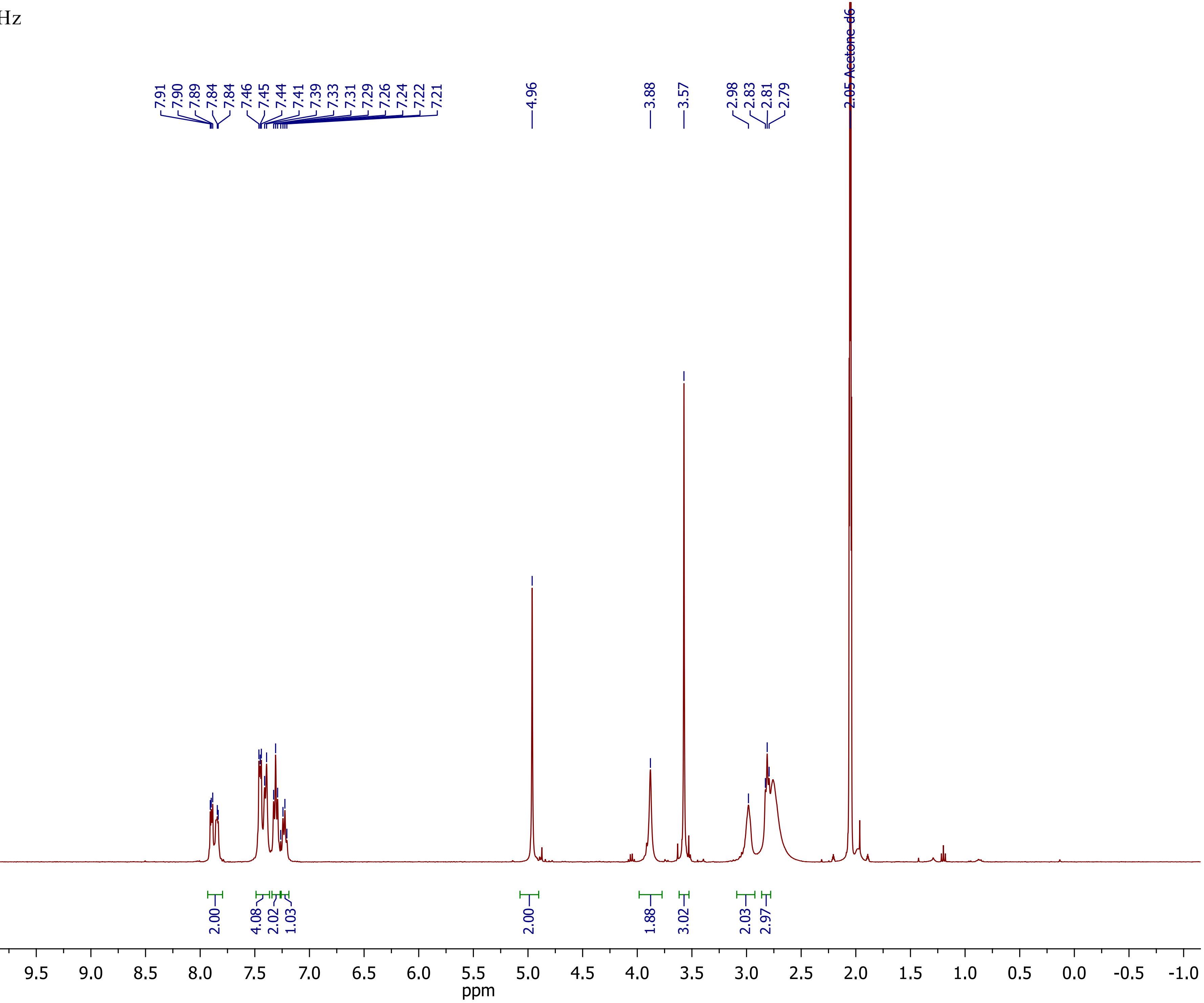

$^{13}C\{^1H\}$ NMR, acetone-d_6, 101 MHz

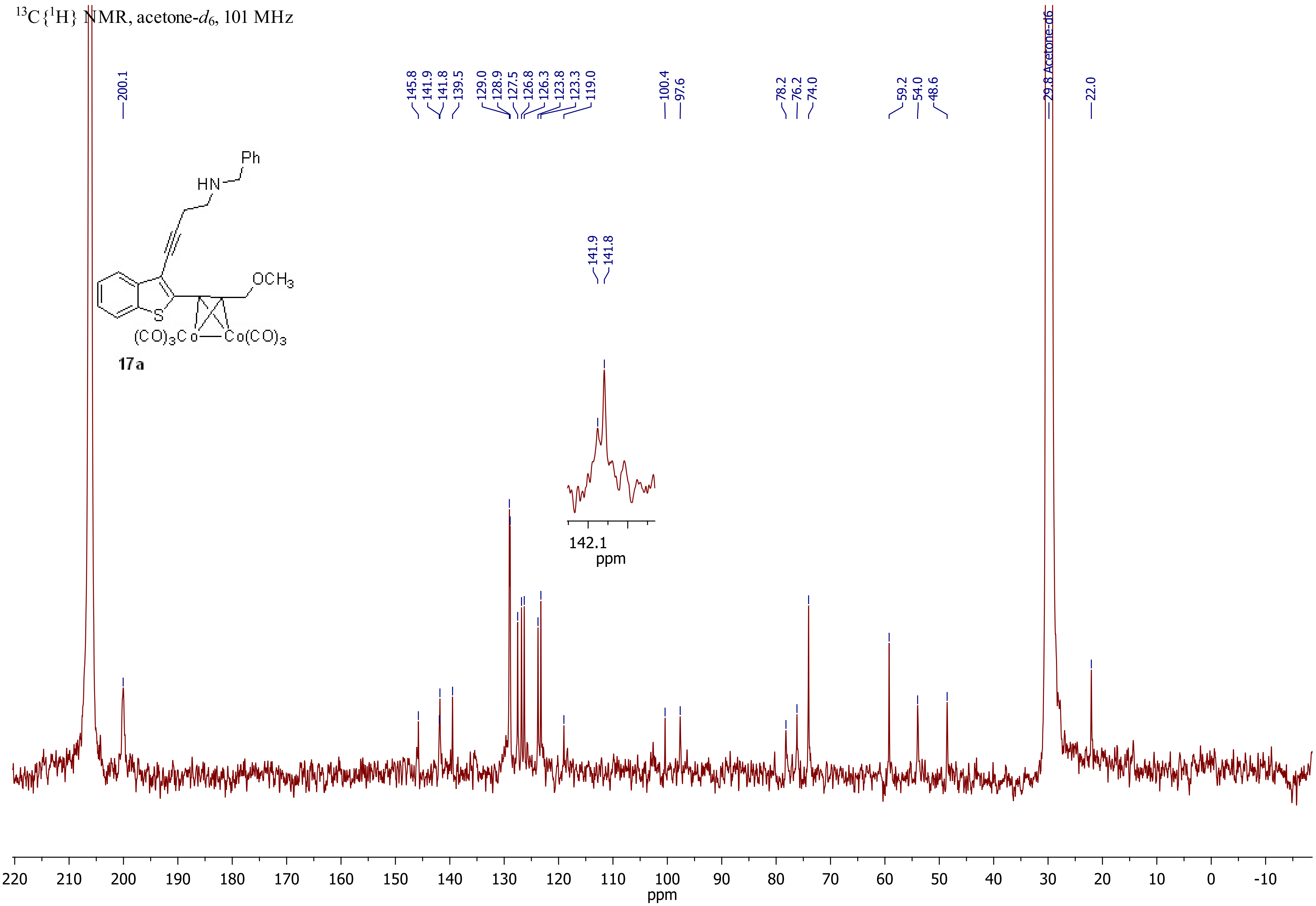

DEPT NMR, acetone-d_6, 101 MHz

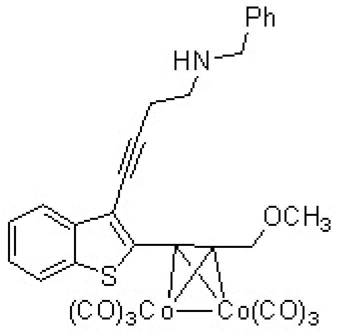

17a

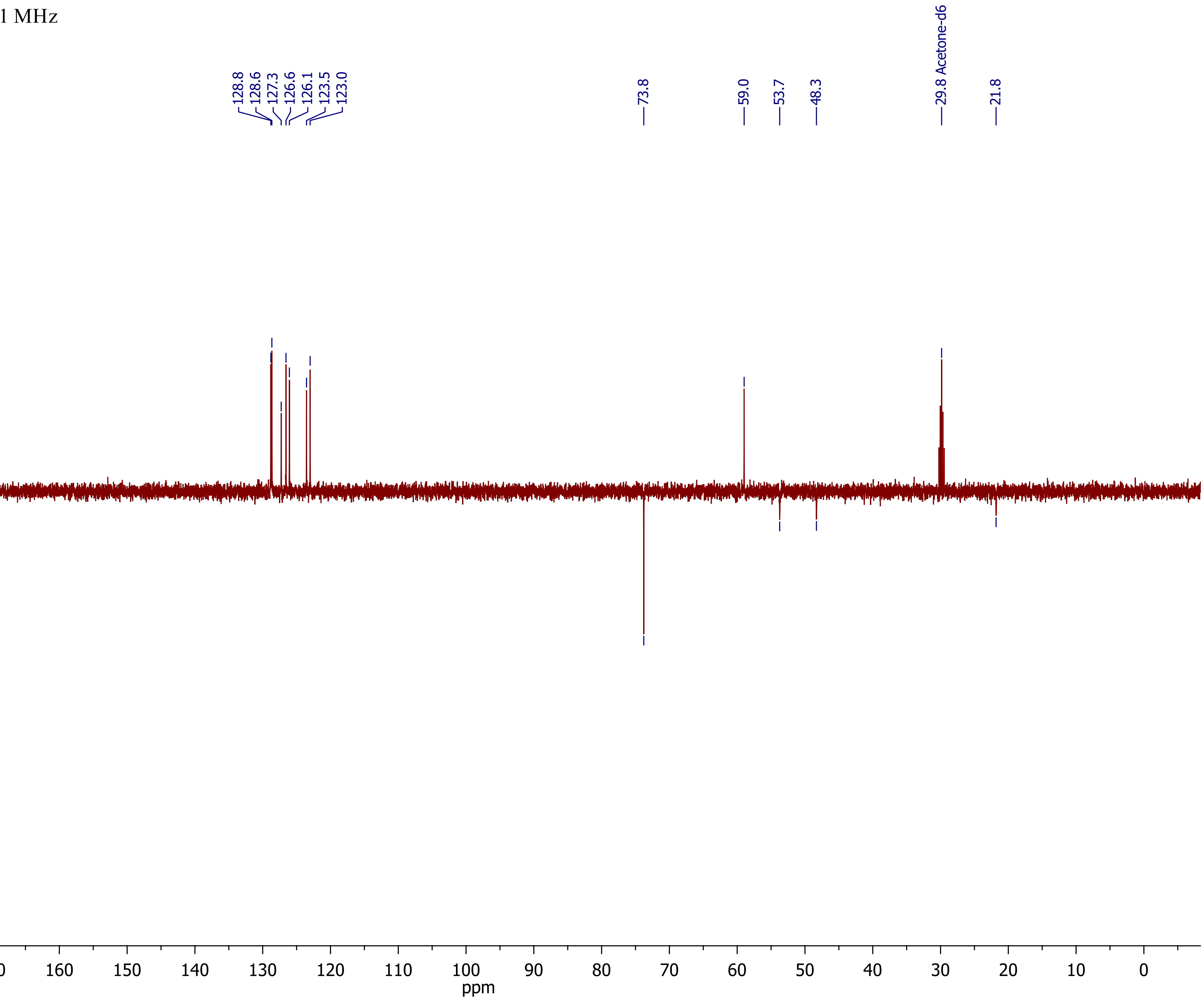

^{1}H NMR, $CDCl_3$, 400 MHz

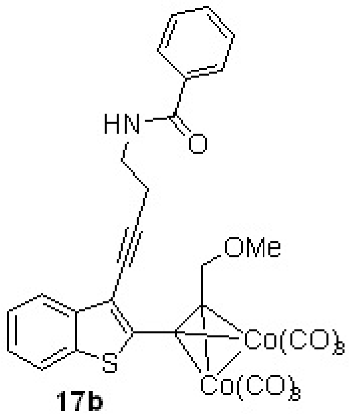

17b

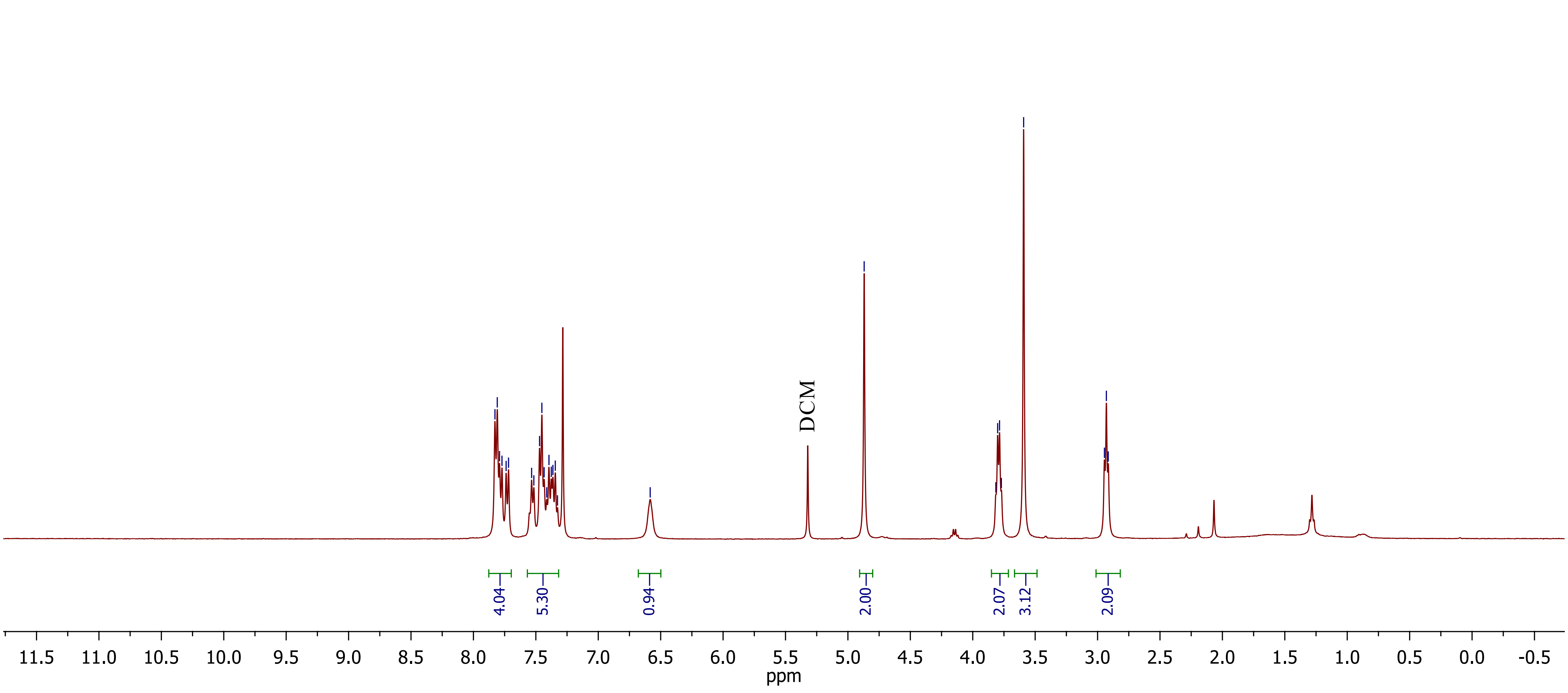

$^{13}C\{^1H\}$ NMR, $CDCl_3$, 125 MHz Only signals from CO ligands and from carbon atoms bonded to hydrogen atoms can be detected by $^{13}C\{^1H\}$ NMR.

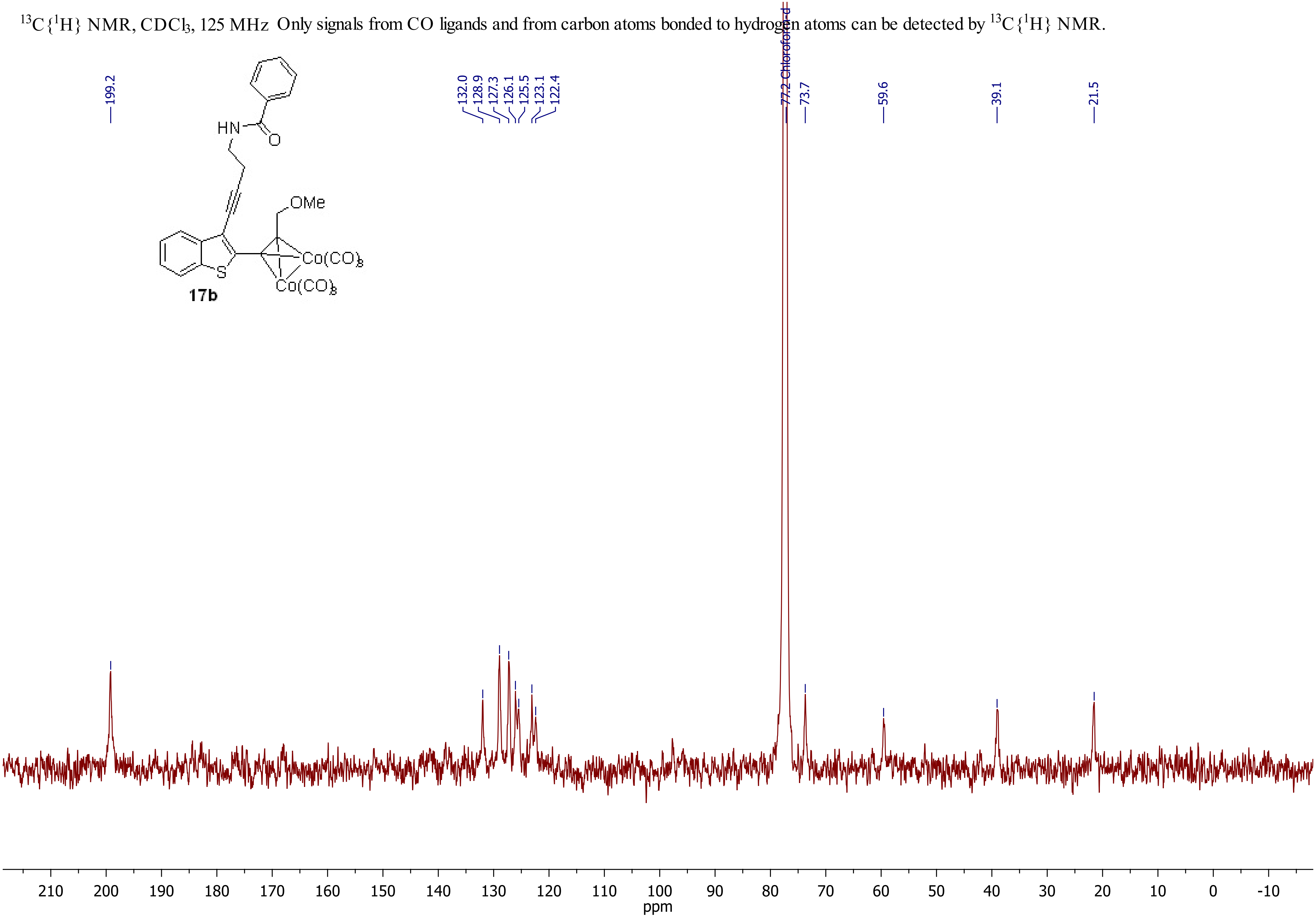

^{1}H NMR, acetone-d_6, 400 MHz

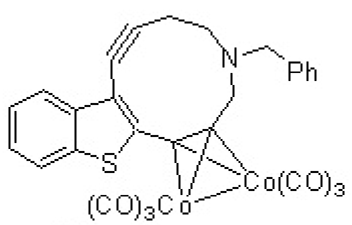

18

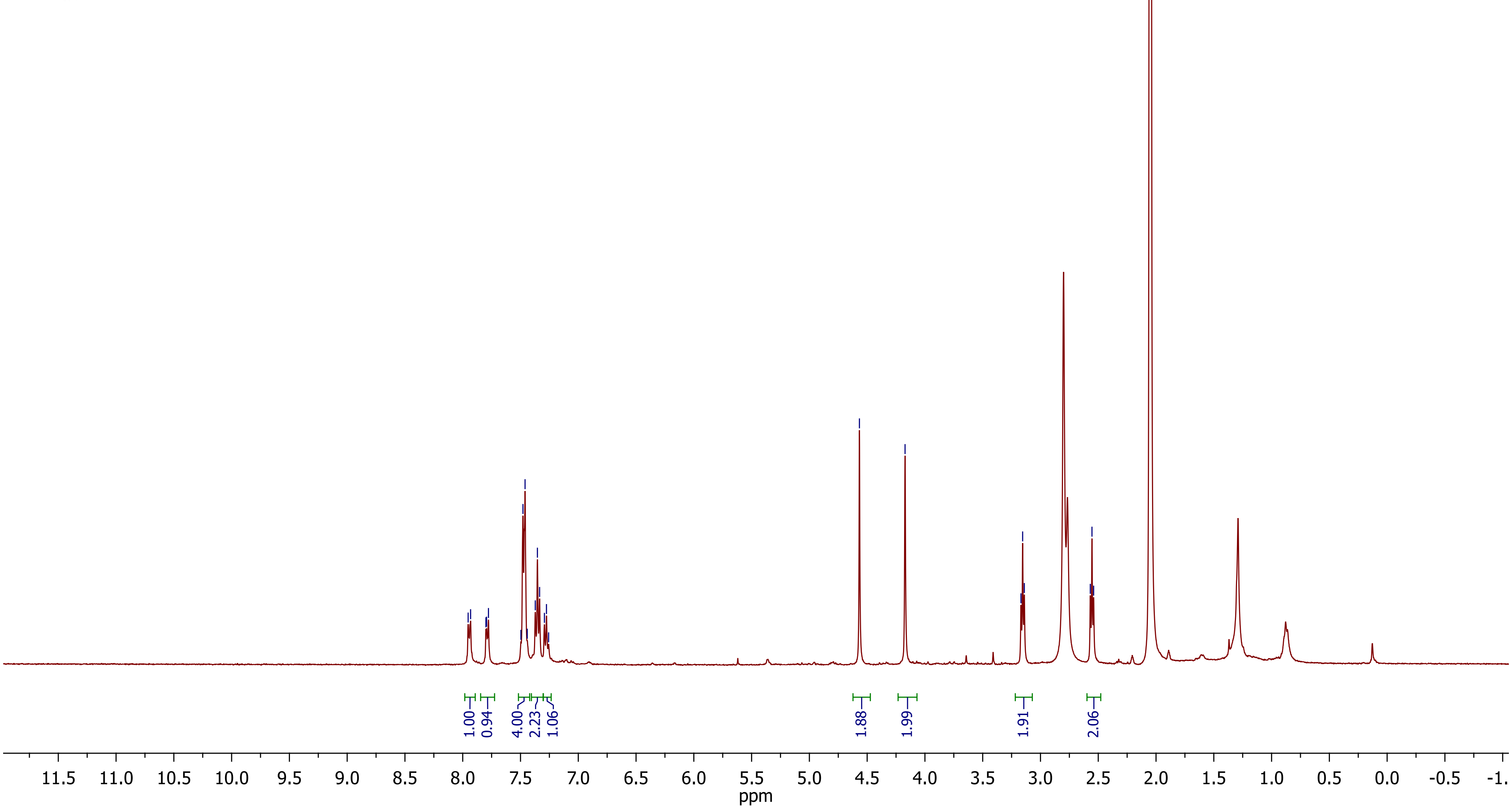

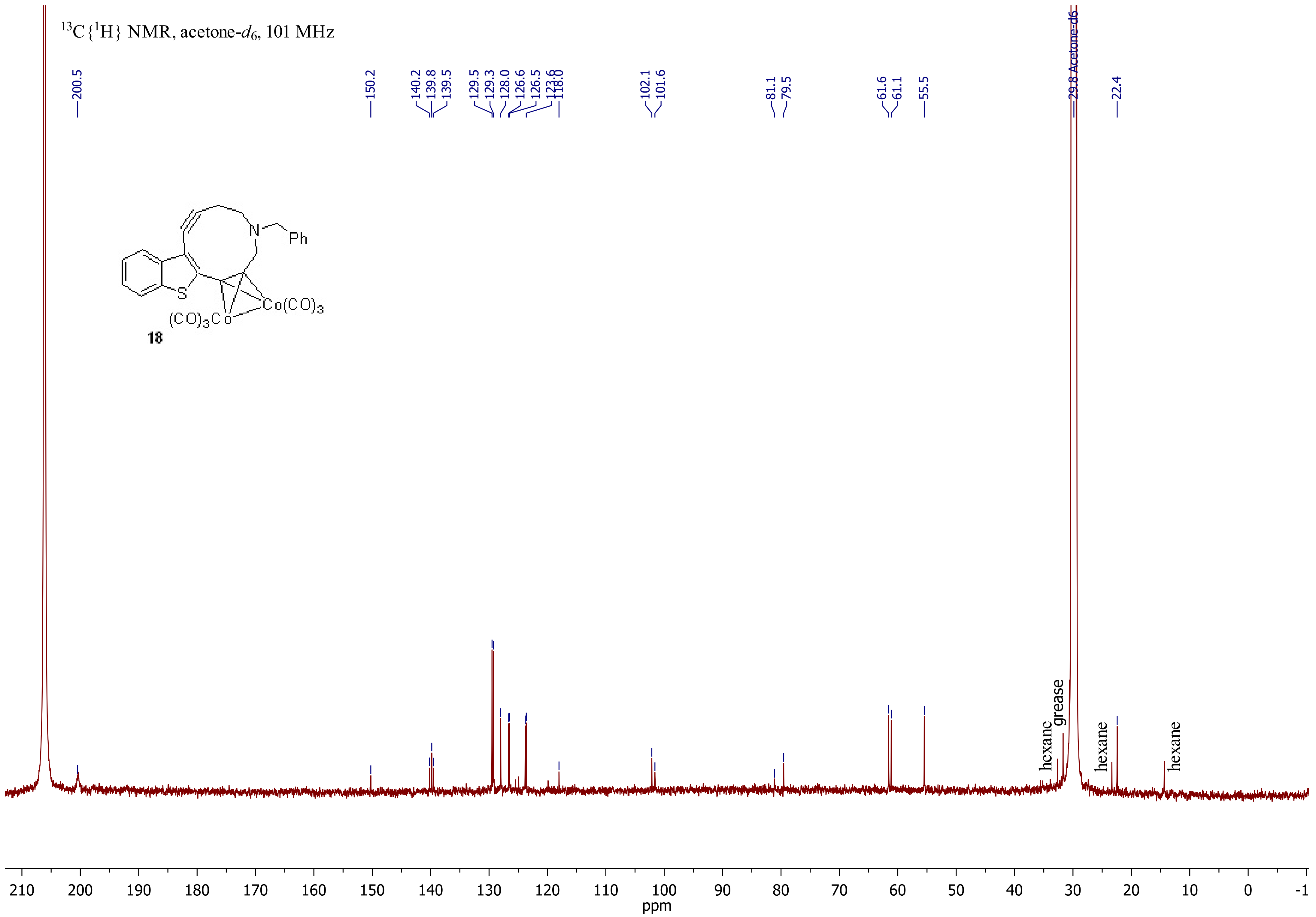
13C{1H} NMR, acetone-d6, 101 MHz
200.5
150.2
140.2
139.8
139.5
129.5
129.3
128.0
126.6
126.5
123.6
118.0
102.1
101.6
81.1
79.5
61.6
61.1
55.5
29.8 Acetone-d6
22.4
N
Ph
S
Co(CO)3
(CO)3Co
18
hexane
grease
hexane
hexane
ppm

^{1}H NMR, $CDCl_3$, 400 MHz

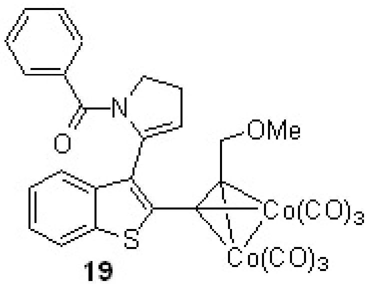

19

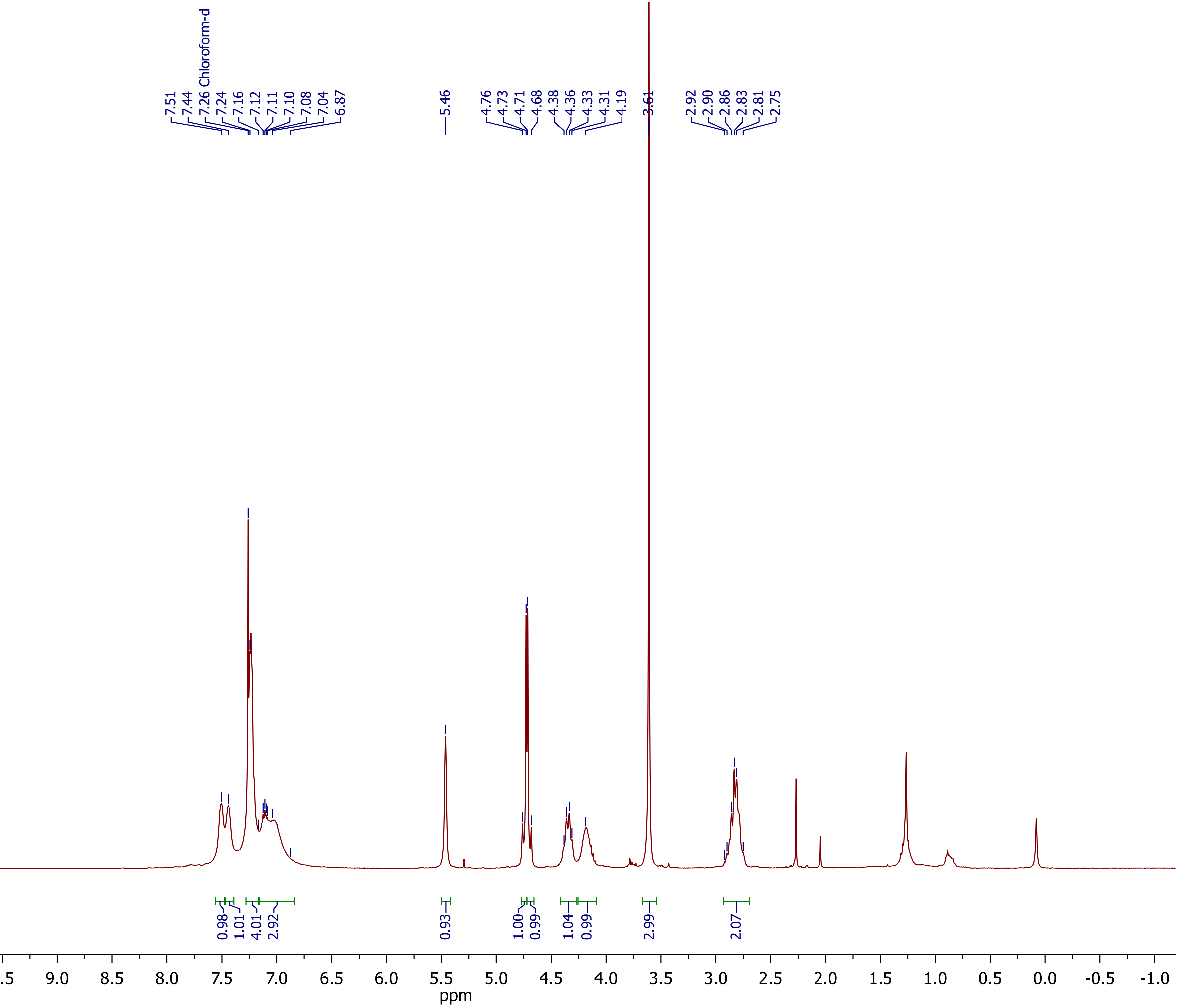

DEPT NMR, $CDCl_3$, 101 MHz

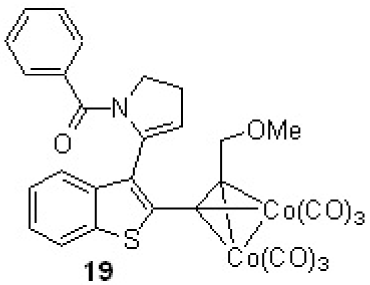

19

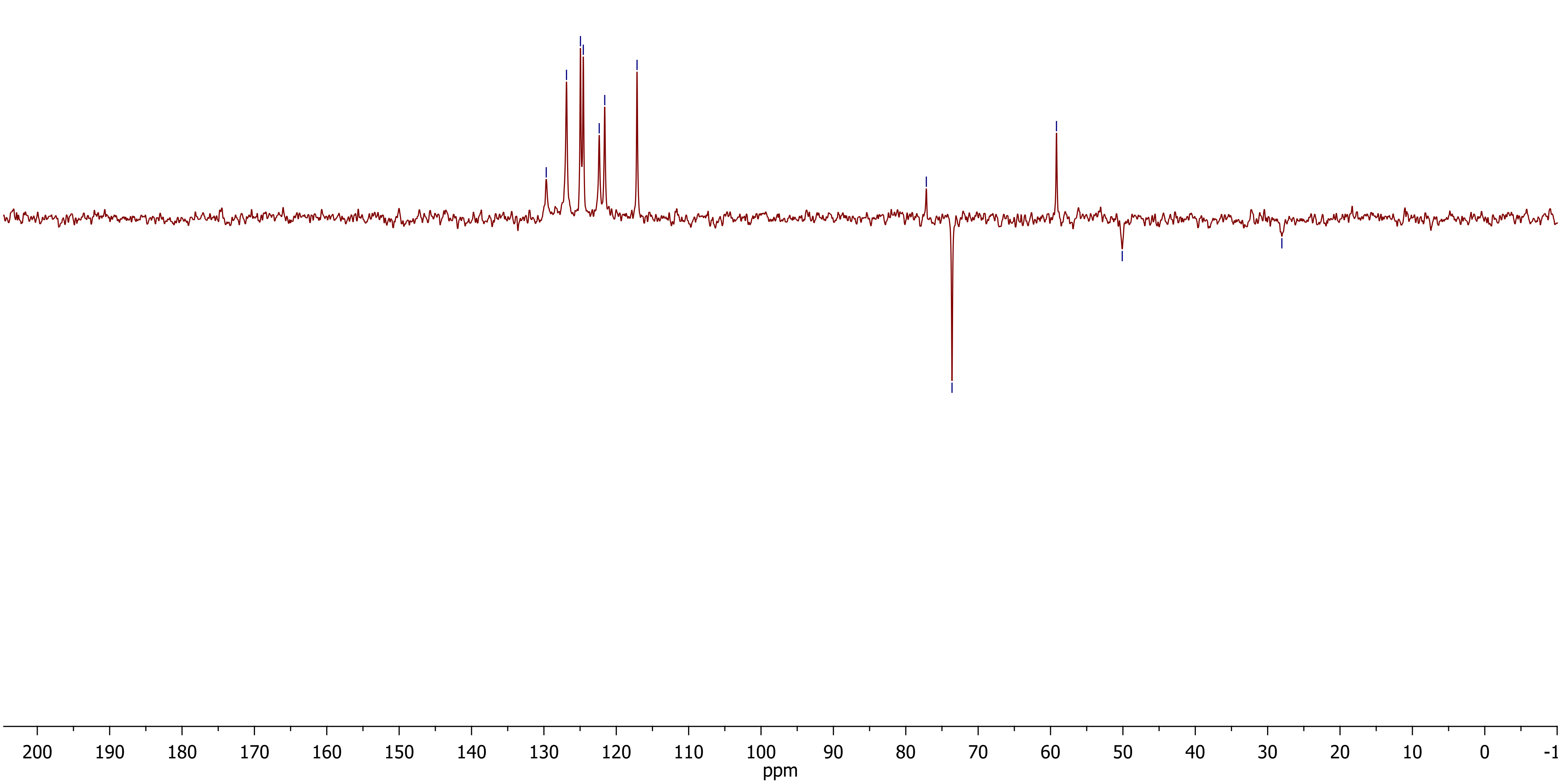

HSQC NMR, $CDCl_3$, 400 MHz, 101 MHz

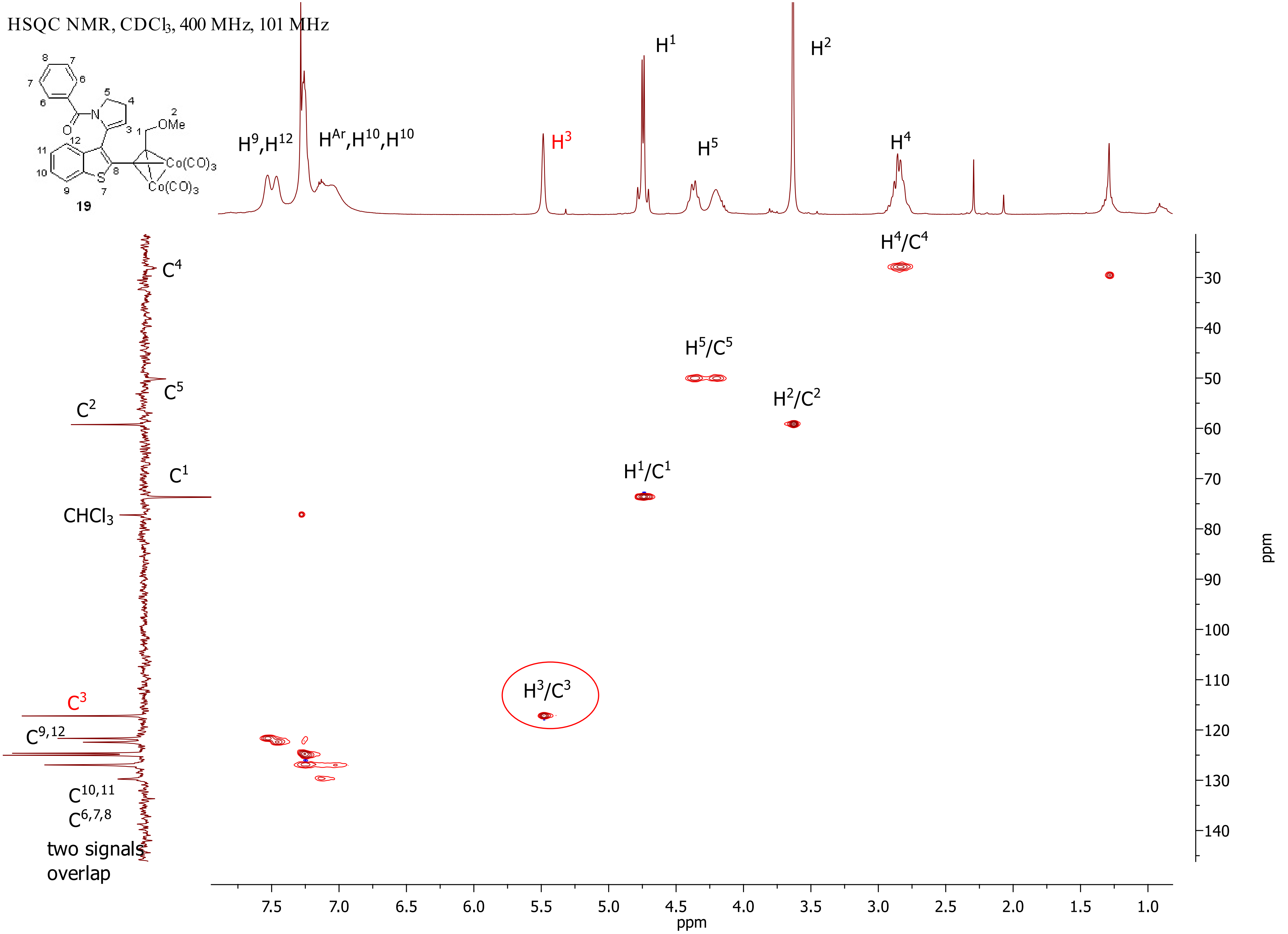

COSY NMR, $CDCl_3$, 400 MHz

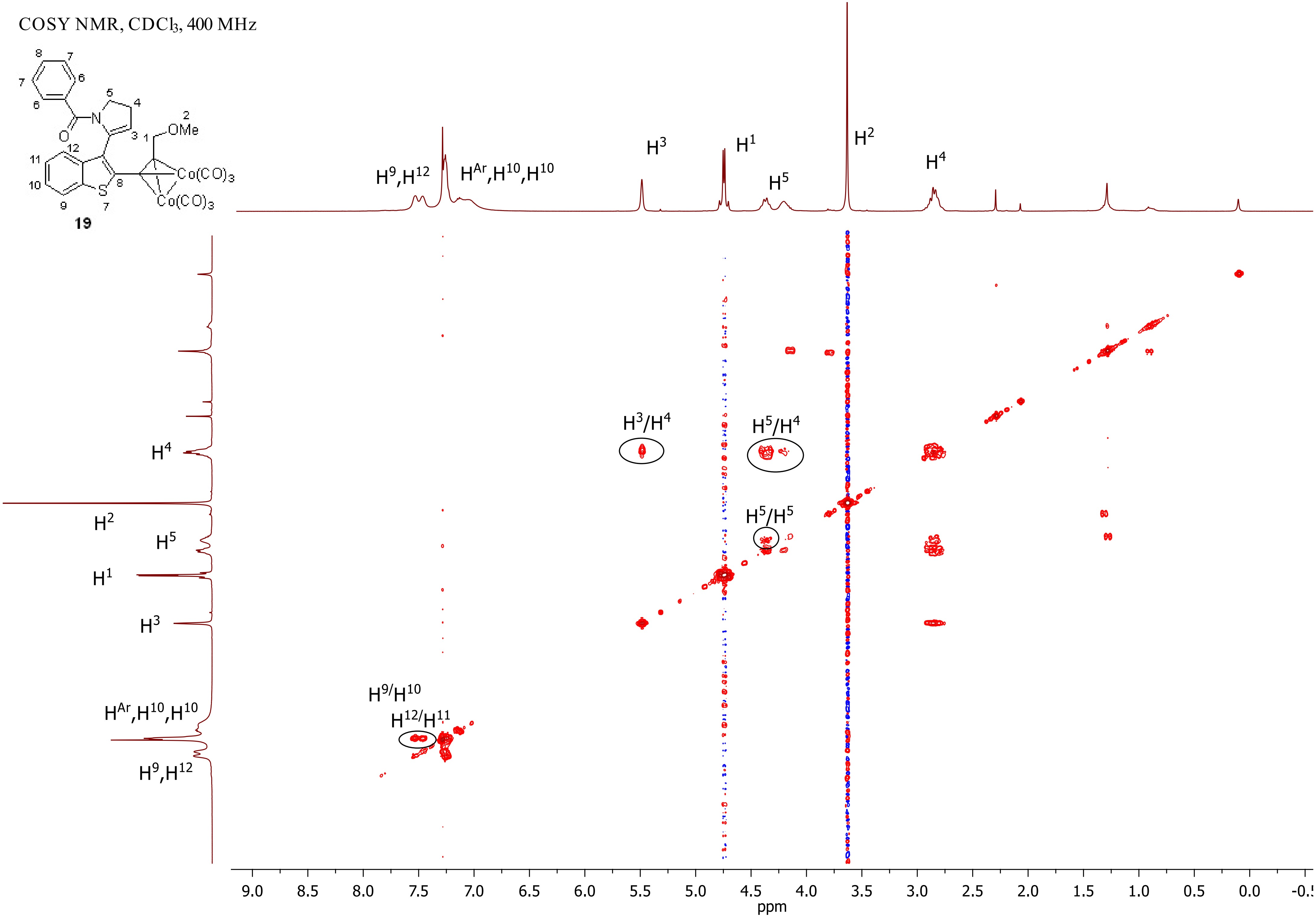

NOESY NMR, $CDCl_3$, 400 MHz

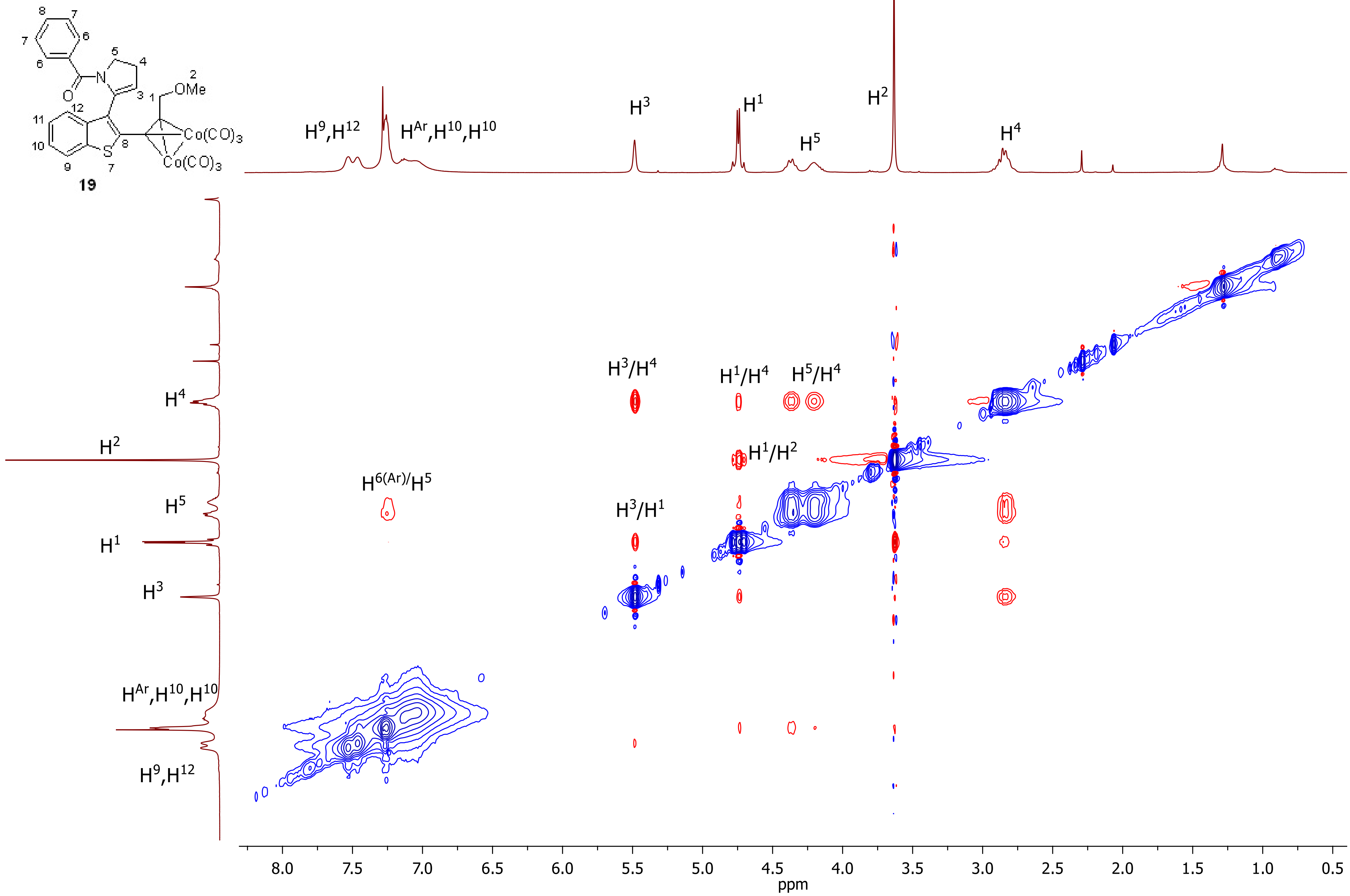

Mass Spectrum Report

Analysis Info

Analysis Name	D:\Data\2018\may\17\DNA450.d	Acquisition Date	17.05.2018 16:03:25
Method	tune_low.m	Operator	Bruker Customer
Sample Name	DNA450	Instrument / Ser#	micrOTOF 10223
Comment	MeOH		

Acquisition Parameter

Source Type	ESI	Ion Polarity	Positive	Set Nebulizer	0.4 Bar
Focus	Not active			Set Dry Heater	180 °C
Scan Begin	50 m/z	Set Capillary	4500 V	Set Dry Gas	4.0 l/min
Scan End	3000 m/z	Set End Plate Offset	-500 V	Set Divert Valve	Source

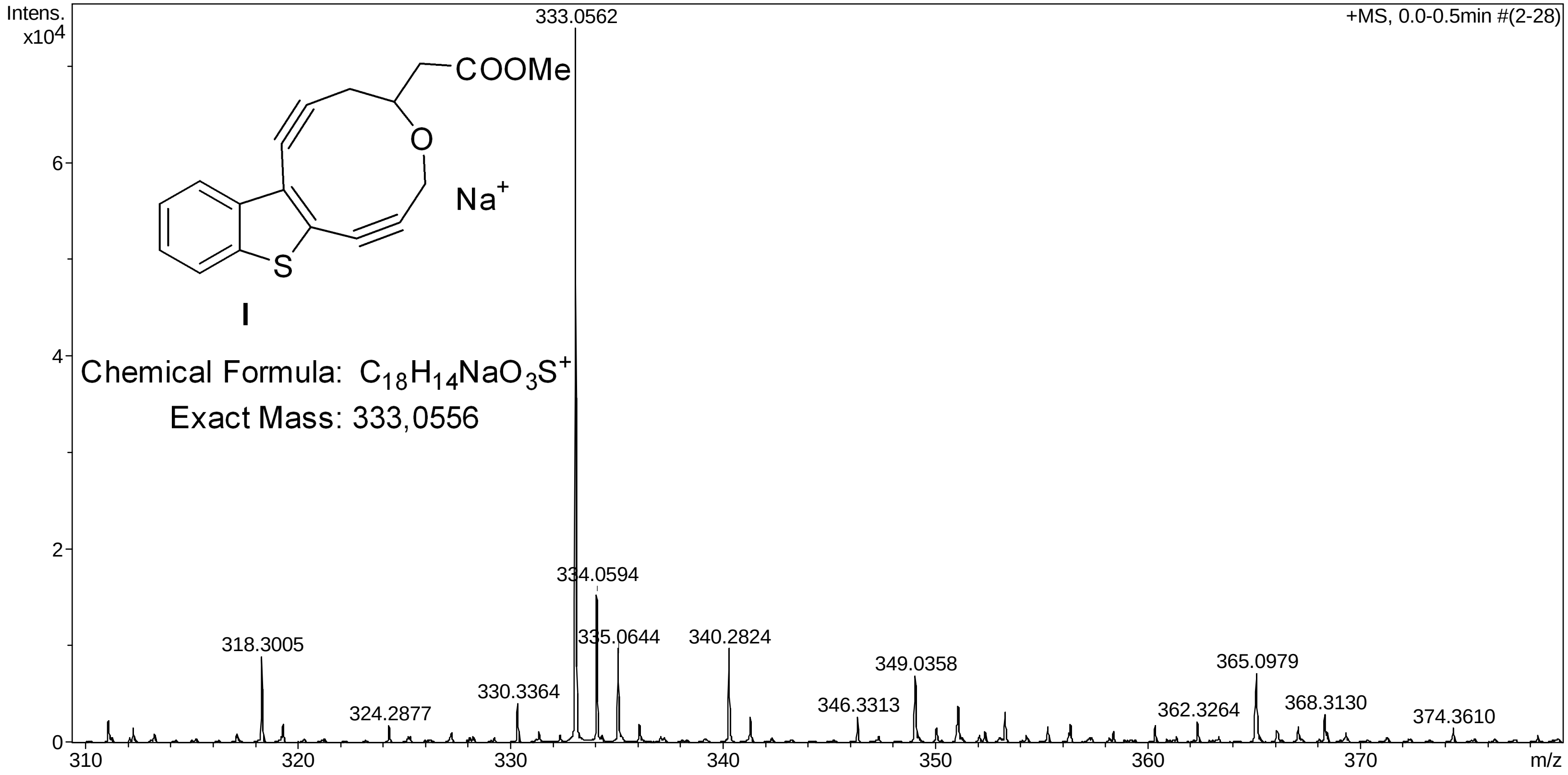

MASS SPECTRUM REPORT

Instrument Name: Shimadzu Nexera X2 LCMS-9030
Sample Name: **EKh_79**
Acquired: 30/05/2022
Interface: **ESI**
Nebulizing Gas Flow: 3,0 L/min
Heating Gas: On
Heating Gas Flow: 10,0 L/min

Interface heater: Off
Interface Temperature: 45 C
Drying Gas:
Drying Gas Flow: 10,0 L/min
DL Temperature: 120 C
Heat Block: 120 C

Saint-Petersburg State University Research Park

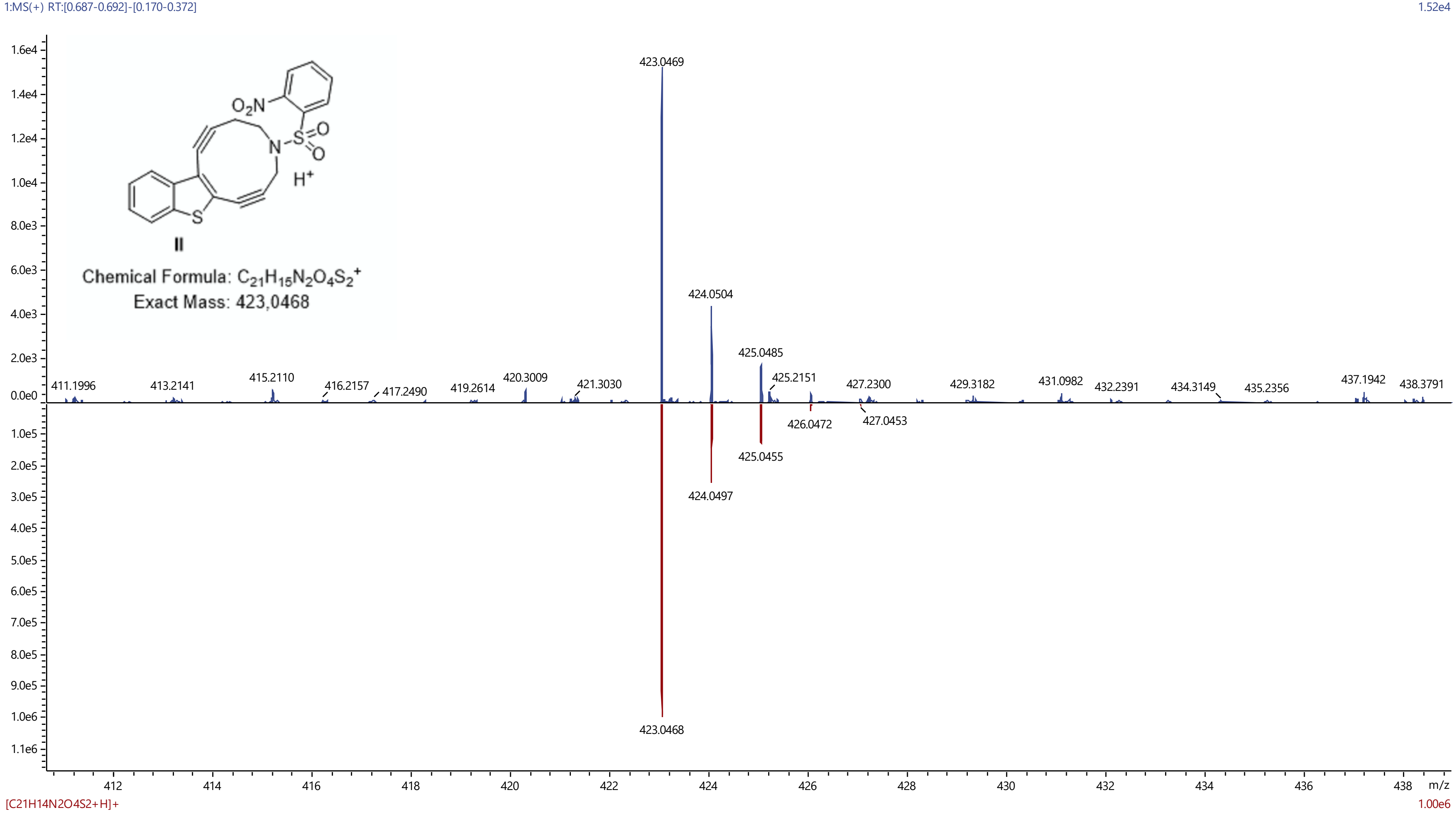

MASS SPECTRUM REPORT

Instrument Name: Shimadzu Nexera X2 LCMS-9030
Sample Name**: Ekh_83**
Acquired: 12/05/2022
Interface: **ESI**
Nebulizing Gas Flow: 3,0 L/min
Heating Gas: On
Heating Gas Flow: 10,0 L/min

Interface heater: Off
Interface Temperature: 45 C
Drying Gas:
Drying Gas Flow: 10,0 L/min
DL Temperature: 120 C
Heat Block: 120 C

Saint-Petersburg State University Research Park

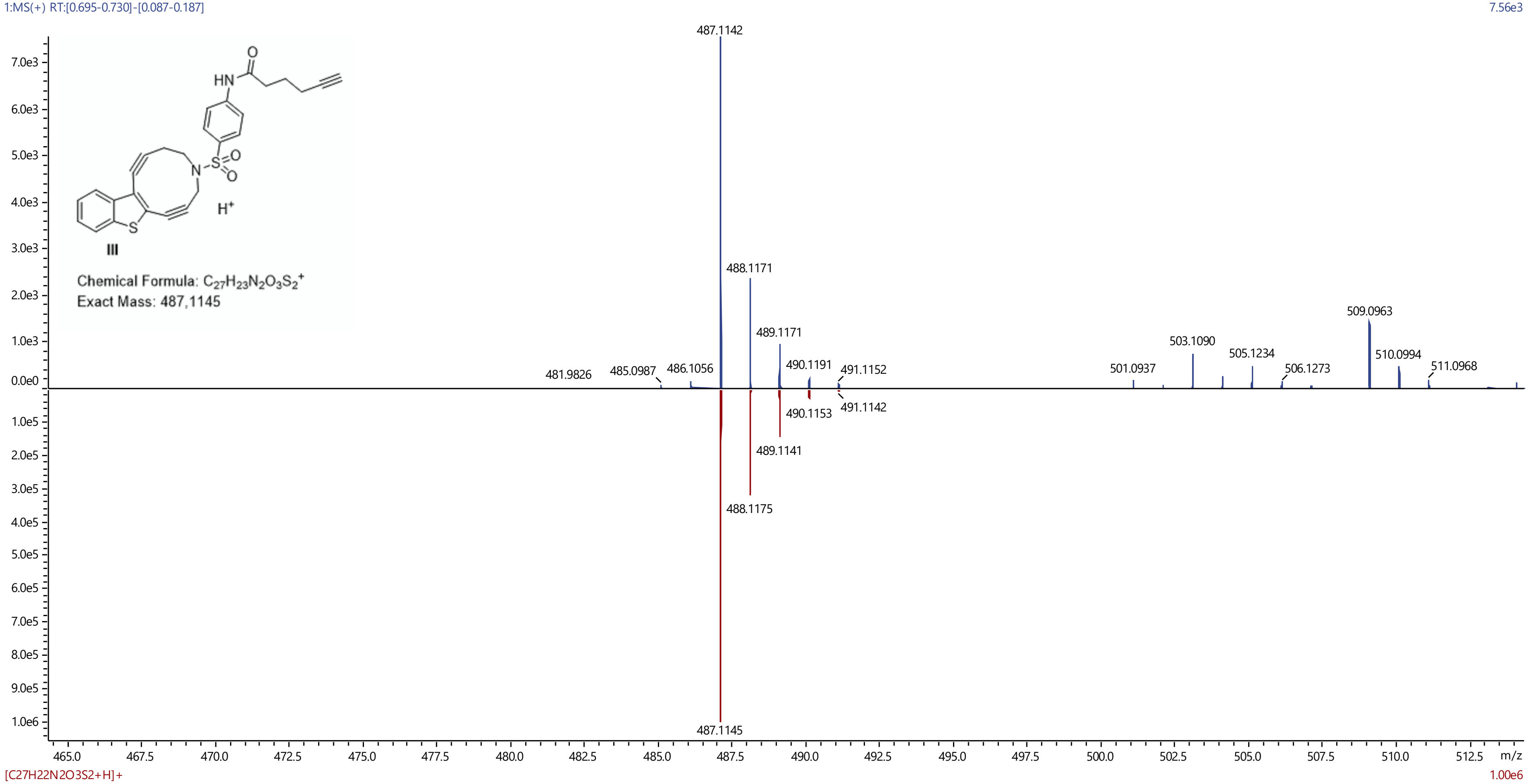

Display Report

Analysis Info

Analysis Name	D:\Data\Work\2018\January\24\DNA58.d	Acquisition Date	1/24/2018 1:29:20 PM
Method	tune_low_pos.m	Operator	BDAL@DE
Sample Name	DNA58	Instrument	maXis 62
Comment	MeOH		

Acquisition Parameter

Source Type	ESI	Ion Polarity	Positive	Set Nebulizer	1.0 Bar
Focus	Active	Set Capillary	4500 V	Set Dry Heater	180 °C
Scan Begin	50 m/z	Set End Plate Offset	-500 V	Set Dry Gas	4.0 l/min
Scan End	1200 m/z	Set Collision Cell RF	300.0 Vpp	Set Divert Valve	Source

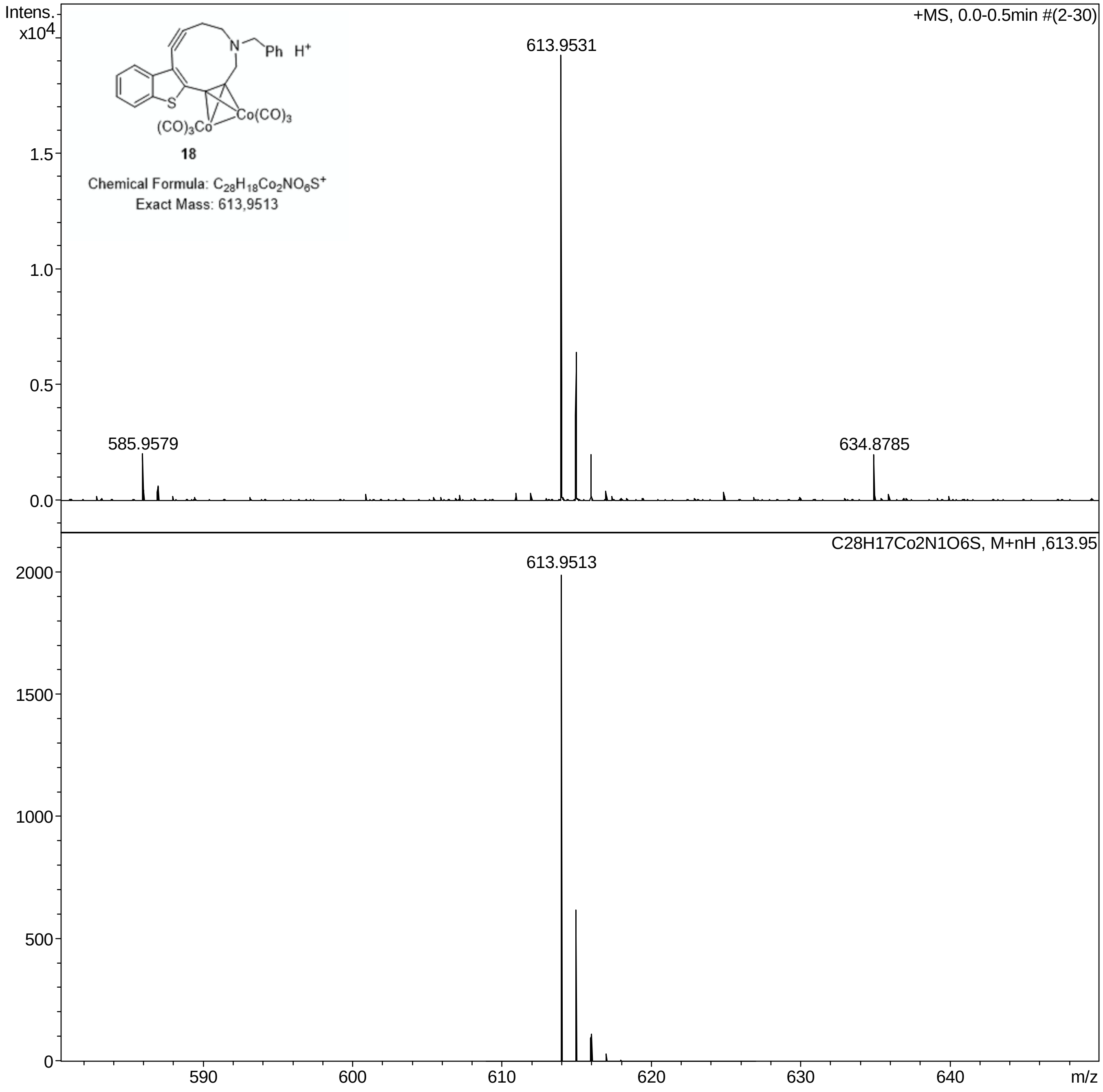

Create PDF files without this message by purchasing novaPDF printer (http://www.novapdf.com)

Mass Spectrum Report

Analysis Info

Analysis Name	D:\Data\2020\february\21\R90.d	Acquisition Date	21.02.2020 12:28:31
Method	tune_wide.m	Operator	Bruker Customer
Sample Name	R90	Instrument / Ser#	micrOTOF 10223
Comment	MeOH		

Acquisition Parameter

Source Type	ESI	Ion Polarity	Positive	Set Nebulizer	0.4 Bar
Focus	Not active			Set Dry Heater	180 °C
Scan Begin	50 m/z	Set Capillary	4500 V	Set Dry Gas	4.0 l/min
Scan End	3000 m/z	Set End Plate Offset	-500 V	Set Divert Valve	Source

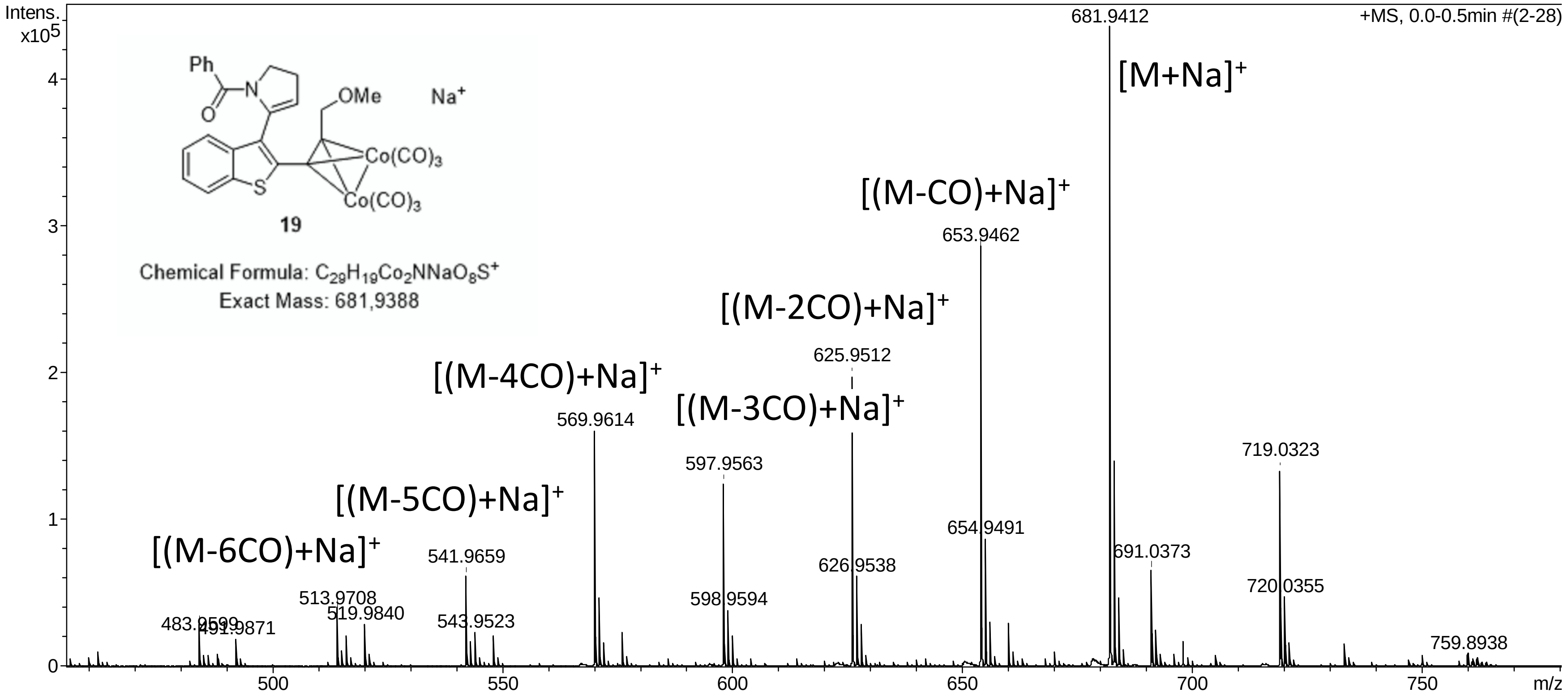

Supplement: Supplementary file 1 [file molecules-27-06071-s001.zip › molecules-1926601-supplementary.pdf]
